# Topology and Excited State Multiplicity as Controlling Factors in the Carbazole-Photosensitized CPD Formation and Repair

**DOI:** 10.1021/acs.joc.2c00942

**Published:** 2022-08-18

**Authors:** Gemma M. Rodríguez-Muñiz, Miguel Gomez-Mendoza, Paula Miro, Pilar García-Orduña, German Sastre, Miguel A. Miranda, M. Luisa Marin

**Affiliations:** † Instituto de Tecnología Química, Universitat Politècnica de València-Consejo Superior de Investigaciones Científicas, Avenida de los Naranjos s/n, 46022 Valencia, Spain; ‡ Dpto. Química Inorgánica, ISQCH-Instituto de Síntesis Química y Catálisis Homogénea, Facultad de Ciencias, CSIC-Universidad de Zaragoza, Pedro Cerbuna 12, 50009 Zaragoza, Spain

## Abstract

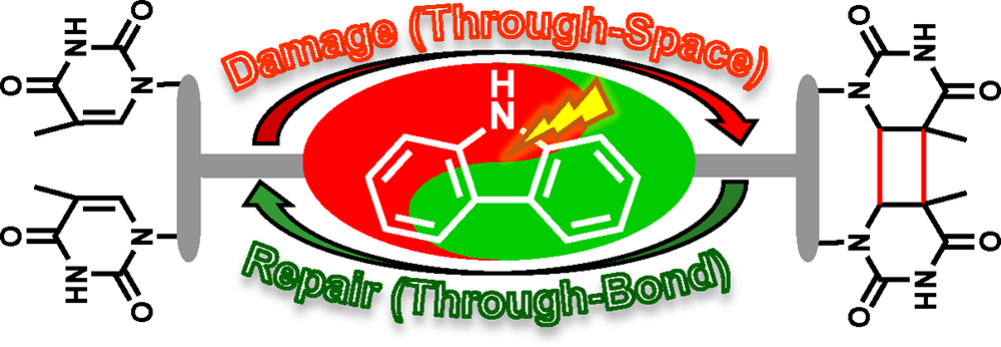

Photosensitized thymine<>thymine (Thy<>Thy) formation and repair can be mediated by carbazole (Cbz). The former occurs from the Cbz triplet excited state via energy transfer, while the latter takes place from the singlet excited state via electron transfer. Here, fundamental insight is provided into the role of the topology and excited state multiplicity, as factors governing the balance between both processes. This has been achieved upon designing and synthesizing different isomers of trifunctional systems containing one Cbz and two Thy units covalently linked to the rigid skeleton of the natural deoxycholic acid. The results shown here prove that the Cbz photosensitized dimerization is not counterbalanced by repair when the latter, instead of operating through-space, has to proceed through-bond.

## 1 Introduction

Solar light arriving at Earth is essential for humans, but at the same time, it is responsible for serious deleterious effects. Although UVB radiation represents only a minor sunlight component, it is associated to melanoma skin cancer, since it can be absorbed by the thymine (Thy) or cytosine (Cyt) nucleobases. As a consequence, formation of cyclopyrimidine dimers, (CPDs) such as Thy<>Thy,^1^ Thy<>Cyt,^2,3^ or Cyt<>Cyt, as well as pyrimidine (6–4) pyrimidone adducts and their related Dewar isomers, are observed.^4,5^ The Thy<>Thy dimers are the photoproducts obtained with higher yields (likely because Cyt exhibits the highest energy triplet among the DNA bases)^6^ and also the most biologically significant.^7−9^ In addition, the effects of UVA should not be disregarded, in particular when they can be mediated by photosensitizers absorbing in this region. A limited number of photosensitizers have been employed to investigate this DNA photodamage, including non-steroidal anti-inflammatory drugs, fluoroquinolones, ketones, pyridopsoralenes, or *p*-aminobenzoic acid derivatives.^10−12^ The reported quantum yields for photosensitized dimerization range from 10^–5^ to 10^–2^. Formation of Thy<>Thy dimers is thought to proceed through an initial triplet–triplet energy transfer step (TTET).^13,14^ Efficient TTET requires, in principle, a donor chromophore with a high intersystem crossing quantum yield, a triplet energy above that of Thy, and a long triplet lifetime. In general, Thy<>Thy dimer formation follows an exponential distance dependence as expected from a Dexter-type TTET mechanism.^15,16^ Nevertheless, alternative mechanisms involving the participation of triplet triplexes have been demonstrated to play a role in the photosensitized formation of Thy<>Thy mediated by benzophenone (Bzp).^17^ Moreover, recent examples have demonstrated the generation of ^3^Thy* at long (non-bonding) distance through-bond (TB), in intramolecular systems in which the photosensitizer and the nucleobase are separated by a rigid hydrocarbon bridge.^18^

In prokaryotes, yeast, and plants, CPDs are repaired by photolyases. They act through a light-dependent single-electron transfer mechanism^19,20^ with quantum yields for the repair of Thy<>Thy as high as 0.7–0.98.^21^ The redox-active flavin adenine dinucleotide (FAD) cofactor plays a pivotal role in the photorepair activity of photolyases, since its fully reduced and protonated form (FADH^–^) can be directly excited to reach its singlet excited state (^1^FADH^–*^) or, more efficiently, *via* energy transfer from an antenna chromophore present in the medium. Then, the excited ^1^FADH^–*^ transfers one electron to the CPD and leads to the dimer radical anion, inducing the spontaneous cleavage of the cyclobutane, finally giving rise to the restored pyrimidines. The limiting factor for the repair efficiency seems to be the back electron transfer from the dimer radical anion and the electron donor.^22^ Model compounds, which mimic the performance of the CPD-photolyase, have been reported to achieve the CPD photosensitized repair.^22−27^ Among them, the activity of the carbazole (Cbz) chromophore has been tested upon incorporation of an artificial Cbz-nucleoside into a DNA duplex^28^ or in covalently linked Cbz-thymine dimer compounds.^29^ This activity relies on the lifetime (*ca.* 19 ns) and redox potential (E*_Cbz_^**·**+^/_Cbz_ = −2.44 V *vs* SCE)^29^ of the Cbz singlet excited state (in the order of the values reported for flavin derivatives),^30^ making it able to produce the e^–^ transfer to the Thy<>Thy (*E*_Thy<>Thy_/_Thy<>Thy_^**·**–^ = −2.2 V *vs* SCE).^29^

Surprisingly, carbazoles can also mediate photosensitized CPD formation in DNA, although the efficiency of this process is lower than expected from the Cbz photophysical properties.^31^ As a matter of fact, Cbz can be excited selectively in the presence of Thy, and also, Cbz exhibits a moderate intersystem crossing quantum yield (0.36),^32^ a relatively high triplet energy (70.2 kcal mol^–1^),^32^ a ππ* triplet configuration^33^ (free from the problems associated with the competitive hydrogen abstraction by benzophenone),^34^ and a reasonably long-living triplet excited state. Moreover, how far the energy migrates in DNA to eventually produce photodamage is still a matter of concern.^35,36^

**Scheme 1:**
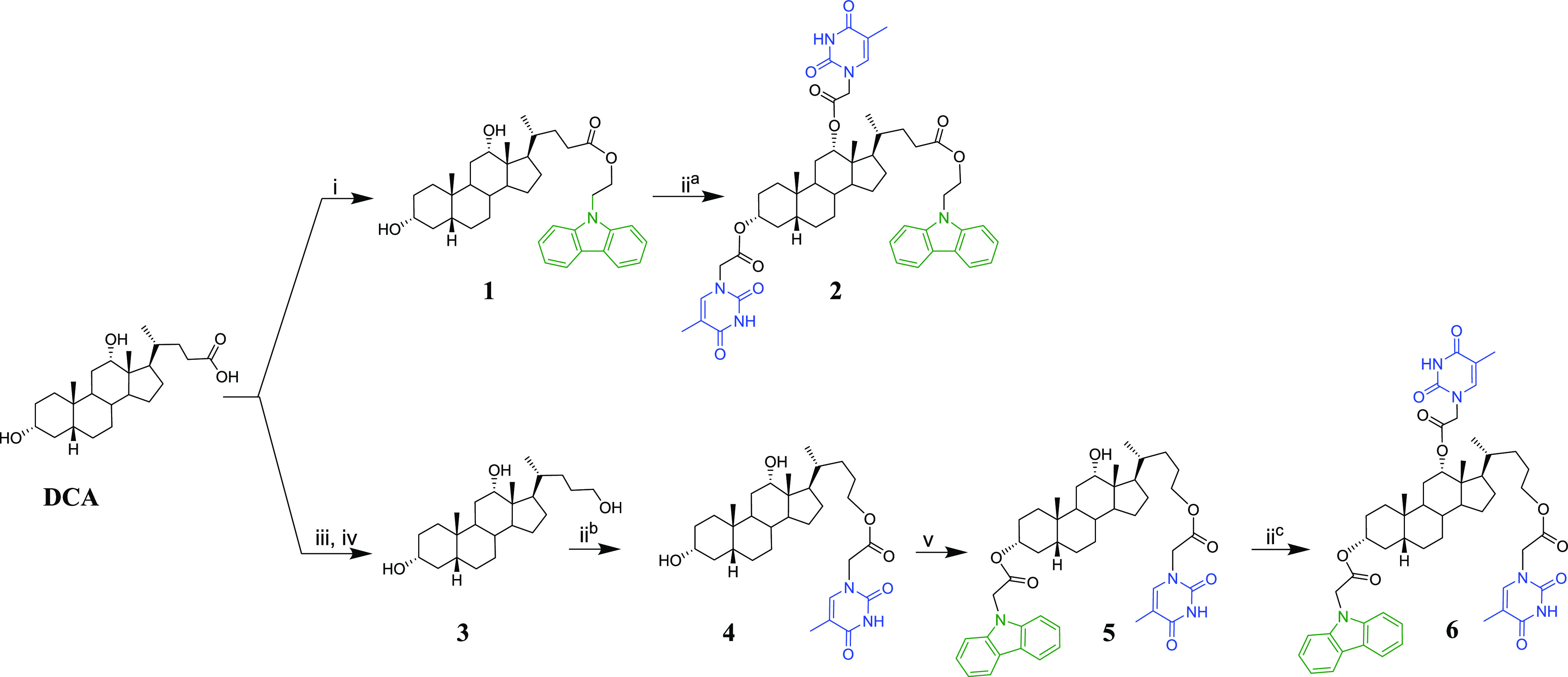
Reagents and Conditions: (i) Cbz-CH_2_CH_2_OH, TBTU, DIEA, and DMF (67%); (ii) Thy-CH_2_COOH, Et_3_N, 2,4,6-Trichlorobenzoyl Chloride, 4-DMAP, and THF (77%)^a^, (56%)^b^, and (60%)^c^; (iii) Benzyl Bromide, DBU, and DMF (57%); (iv) LiAlH_4_ and Refluxing THF (87%); (v) Cbz-CH_2_CO_2_H, TBTU, DIEA, and DMF (43%)

With this background, our aim was to control the balance between DNA damage and repair by the Cbz chromophore. In order to achieve this goal, we have designed appropriate trifunctional intramolecular systems (see Scheme 1). Here, the through-space (TS) TTET mediated by an intramolecular Cbz would result in the formation of Thy<>Thy, in such a way that photosensitized repair should necessarily happen TB. For this purpose, two Thy units and a Cbz will be anchored to the rigid skeleton provided by deoxycholic acid (DCA), preparing different diastereoisomers to evaluate the influence of the topology on the involved processes. The predominance of TTET for Thy<>Thy formation or the e^–^ transfer for Thy<>Thy repair will be modulated through the absence or presence of oxygen in the atmosphere of the reaction media, which will favor the prevalence of the triplet or the singlet excited states of Cbz.

## 2 Results and Discussion

### 2.1 Synthesis

Two new dyads derived from DCA have been synthesized (Scheme 1), containing the Cbz chromophore at the lateral chain and the Thy units at 3α + 12α (**2**) or the Cbz at 3α and two Thy moieties at 12α + the lateral chain (**6**). The developed synthetic strategy started with esterification of DCA with Cbz-CH_2_CH_2_OH to yield **1**. Then, in the presence of an excess of Thy-CH_2_CO_2_H, the positions 3α and 12α were esterified providing **2**. To prepare the derivative with the two Thy moieties at 12α and at the lateral chain, initially, the carboxyl group at DCA was reduced to the corresponding alcohol (**3**), and the Thy at the lateral chain was covalently attached using Thy-CH_2_CO_2_H to give **4**. The following step was the introduction of the chromophore at 3α to yield **5**. Subsequent treatment with ThyCH_2_CO_2_H provided **6**. In summary, new derivatives in which different combinations of Thy units and distances to the chromophore have been designed, synthesized, and fully characterized (^1^H and ^13^C NMR and exact mass) to investigate the influence of the topology on the photosensitized formation of Thy<>Thy dimers and eventually in their photosensitized repair.

### 2.2 Photosensitized Thy<>Thy Dimer Formation

**Figure 1:**
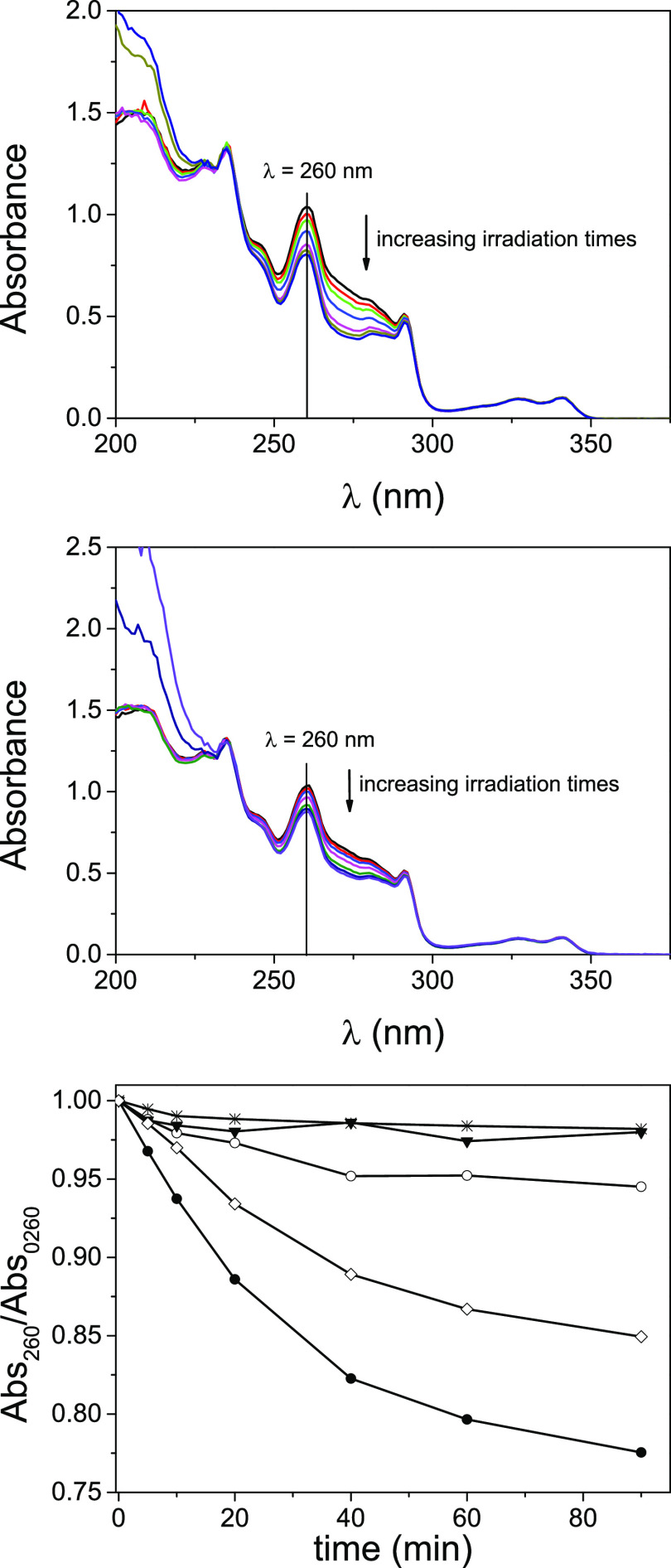
Top: UV–vis spectra of **2** recorded at different irradiation times. Middle: UV–vis spectra of **6** recorded at different irradiation times. Bottom: Photoreaction kinetics of Thy (as ThyCH_2_CO_2_H) (black inverted triangle), Cbz (as Cbz-CH_2_CH_2_OH) (*), the intermolecular 2Thy:1Cbz mixture (open circle), and the intramolecular systems **2** (black circle) and **6** (diamond). All reactions were performed upon irradiation at 350 nm, in deaerated 4CH_3_CN:1H_2_O.

Initially, diluted anaerobic solutions (4.4 × 10^–5^ M in 4CH_3_CN:1H_2_O) of **2** or **6** were submitted to steady-state photolysis. Irradiation was performed at λ_max_ = 350 nm and monitored after different irradiation times attending at the changes in the spectra at 260 nm, where the Thy chromophore has a maximum (Figure 1 top for **2**, middle for **6**, and Figure S5.1 for the control experiments under aerobic conditions). The controls Thy (as ThyCH_2_CO_2_H), Cbz (as Cbz-CH_2_CH_2_OH), and the intermolecular 1Cbz:2Thy mixture showed a slight decrease in the absorbance at 260 nm (Figure 1 and Figure S5.1 bottom); nevertheless, the intramolecular systems were clearly more reactive, although their reaction pattern was slightly different.

**Figure 2:**
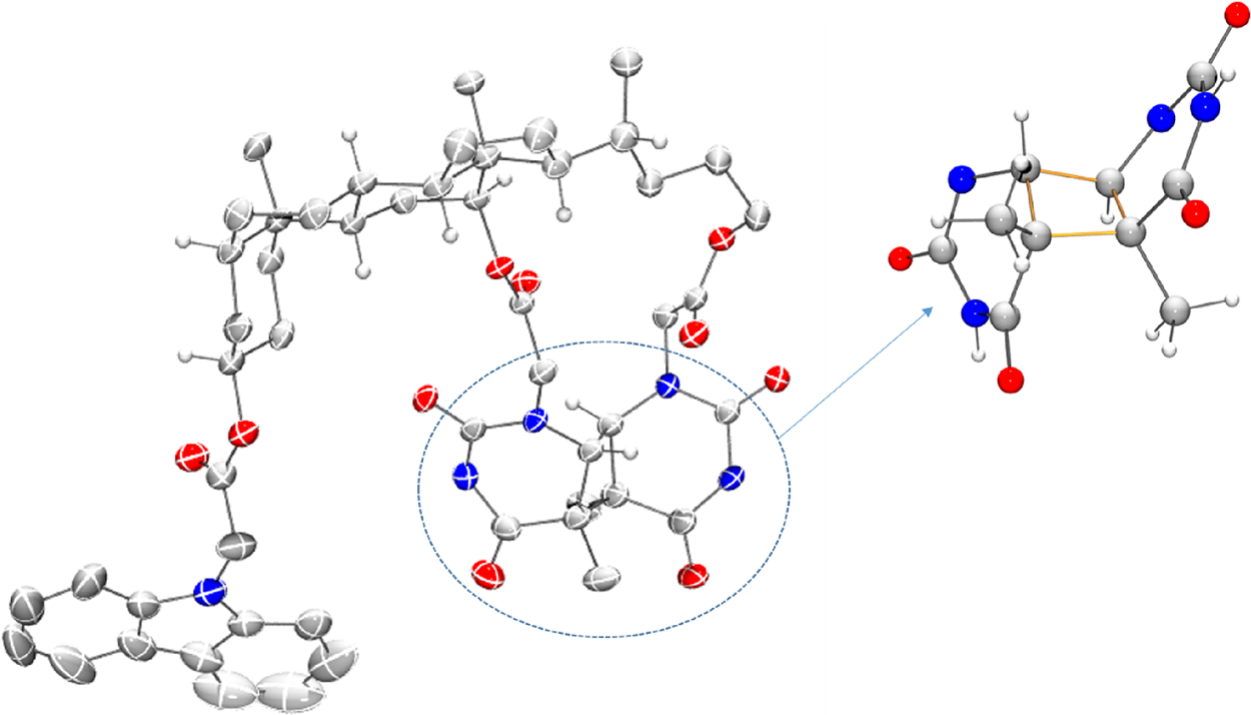
X-ray crystal structure (thermal ellipsoids drawn at the 50% probability level) of the Thy<>Thy **8** resulting from irradiation (λ_max_ = 350 nm) of **6** in CH_3_CN under N_2_, and the detail of the cyclobutane fragment. CCDC 2159900 contains the supplementary crystallographic data for this paper. These data are provided free of charge by The Cambridge Crystallographic Data Centre.

**Scheme 2:**
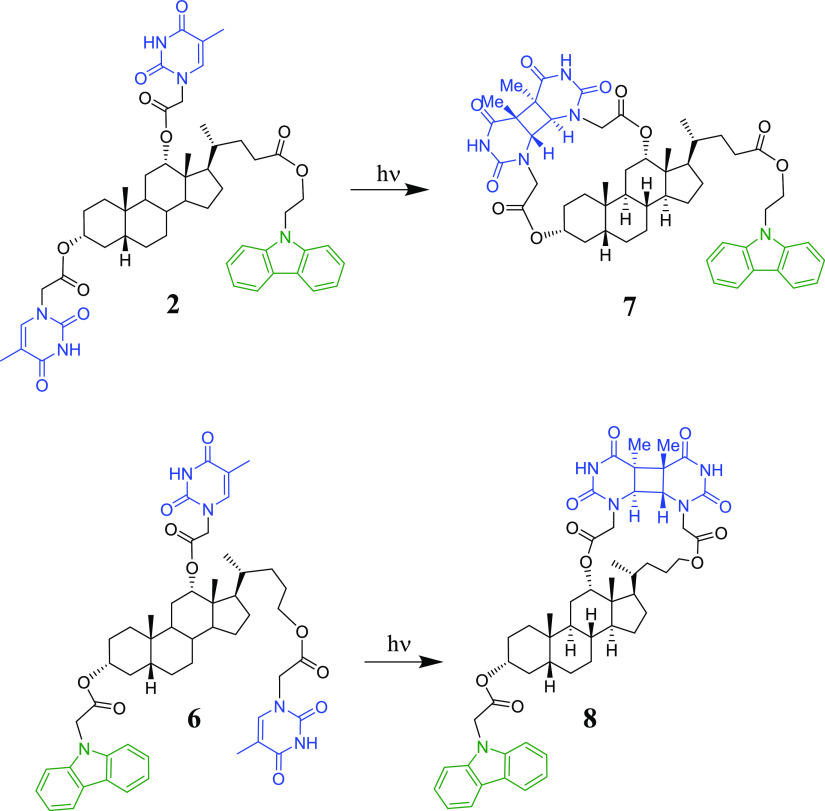
Irradiation (λ_max_ = 350 nm) in Deaerated CH_3_CN of **2** (Top) to Give **7** (>99%) and **6** (Bottom) to Give **8** (68%)

For preparative purposes, more concentrated deaerated solutions of the two dyads (**2** and **6**) in acetonitrile (8.3 × 10^–4^ M and 1.7 × 10^–3^ M, respectively) were independently irradiated (λ_max_ = 350 nm), and only one Thy<>Thy dimer was isolated in each case, in 99 and 68% yields, respectively (Scheme 2). These photoproducts were characterized by ^1^H and ^13^C NMR spectroscopy, together with an exact mass. More specifically, for the case of **7**, upon photosensitized [2 + 2] cycloaddition, the olefinic protons of the two Thy units at 6.96 and 6.98 ppm moved to the cyclobutane protons at 4.13 ppm and *ca*. 4.57 ppm. The corresponding ^13^C-NMR signals moved from 140.7 and 140.5 ppm to 66.4 and 64.6 ppm in the case of the CHs and from 111.0 and 110.5 to 46.0 and 45.9 ppm for the quaternary carbons. Nevertheless, to unambiguously determine the stereochemistry of photoproduct **7** as the one shown in Scheme 2, the ester in the lateral chain was hydrolyzed using titanium(IV) isopropoxide and *in situ* converted into the benzyl ester (**9**) (see Section S4 in the Supporting Information).^37^ Full characterization of compound **9** resulted to be coincident with the 3α,12α-Thy<>Thy-DCABn, previously reported by our group.^17^ Analogously, in the case of **8**, the olefinic protons of the two Thys at 5.53 and 6.99 ppm moved to 4.02 and 4.11 ppm upon formation of the cyclobutane ring. The ^13^C NMR signals corresponding to the characteristic double bond of the Thys moved from 140.2 and 140.0 ppm to 66.3 and 65.9 ppm for the CHs and from 111.7 and 110.6 to 46.2 and 45.5 ppm for the quaternary carbons. We found difficulties in the NOEDIFF experiments due to the NOE zero zone fulfilled by these molecules as a result of their high molecular mass (917.46 g mol^–1^). Nevertheless, the photoproduct was found to be a trans-syn Thy<>Thy, the structure of which was unambiguously established by crystal data (Figure 2, see also the video in the Supporting Information, section S3). A new compound analogous to **6** but without Cbz (**11**) was synthesized starting from **4** to evaluate the role of Cbz in the formation of Thy<>Thy upon 350 nm irradiation (see Sections S4 and S5). Analog **11** resulted to be unreactive upon prolonged irradiation times at 350 nm, under a deaerated atmosphere, confirming the active role of Cbz in the Thy<>Thy formation. Moreover, a compound analogous to **2** without Cbz has already been described and its irradiation only produced the Thy<>Thy dimer in the presence of the absorbing benzophenone.^17^

### 2.3 Photophysics of the TTET in the Photosensitized Thy<>Thy Dimer Formation

**Figure 3:**
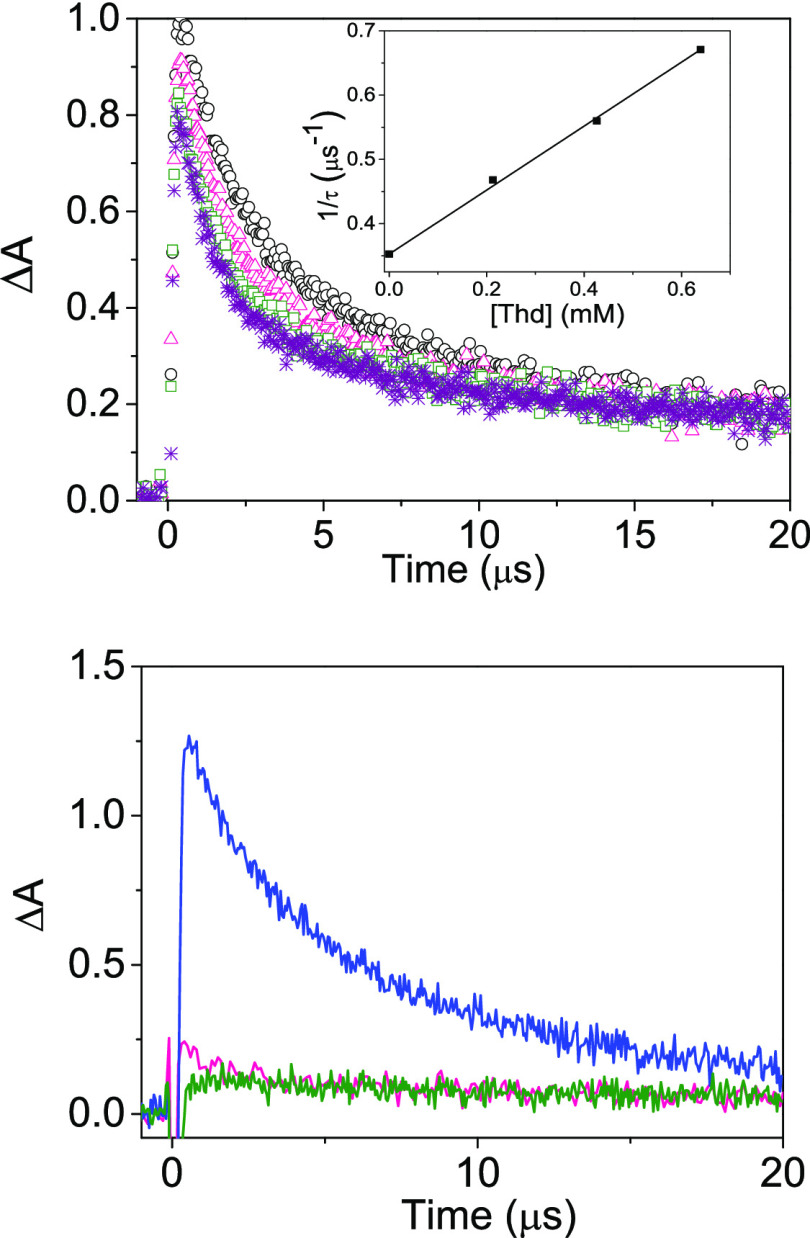
LFP decays obtained upon selective excitation of Cbz at 308 nm and monitored at 420 nm in deaerated 4CH_3_CN:1H_2_O. Top: Cbz (as Cbz-CH_2_CH_2_OH) (black circle) and the intermolecular mixtures, 1Thd:1Cbz (pink triangle), 2Thd:1Cbz (green square), and 3Thd:1Cbz (violet star) in deaerated 4CH_3_CN:1H_2_O. Inset: Stern–Volmer plot for the quenching of ^3^Cbz* by Thd. Bottom: Traces recorded at 420 nm upon excitation of Cbz at 308 nm for **5** (blue), **2** (pink), and **6** (green).

The feasibility of the intermolecular TTET from the triplet of Cbz to Thy was investigated by LFP.^32,38^ The transient absorption spectrum obtained after laser pulse excitation (λ_exc_ = 308 nm) of CbzCH_2_CO_2_H showed a maximum at 420 nm. Thus, the decay of the characteristic ^3^Cbz* obtained upon excitation at 308 nm was recorded upon the addition of one, two, and three equivalents of thymidine (Thd) (to achieve the required concentration), and these data were fitted to a first-order exponential equation (Figure 3, top). The corresponding lifetimes were fitted to a Stern–Volmer relationship, and the value for the intermolecular quenching constant was obtained from the slope of the linear fitting (*k*_q*T*_ = 4.9 × 10^8^ M^–1^ s^–1^). This low value for the quenching constant indicates that intermolecular TTET is efficient. Next, we investigated the TB *vs* TS nature of the TTET from Cbz to the Thy units in the dyads (Figure 3, bottom). This was done by comparing the lifetime of the signals recorded at 420 nm upon selective excitation of Cbz at 308 nm in **5**, **2**, and **6**. The decay corresponding to **5** was fitted to a first-order exponential equation, and the determined lifetime was 4.7 μs; by contrast, the triplet lifetime of **2** and **6** could not be accurately determined. In fact, in both cases, the amplitude of the signal observed just after the laser pulse was very low compared to the one of **5**, which could be attributed to a very efficient quenching of the singlet excited state (see below), together with very efficient TS-TTET, giving rise to the ^3^Thy* that has a very low extinction coefficient at 420 nm.^18^

### 2.4 Photosensitized Thy<>Thy Dimer Repair

**Figure 4:**
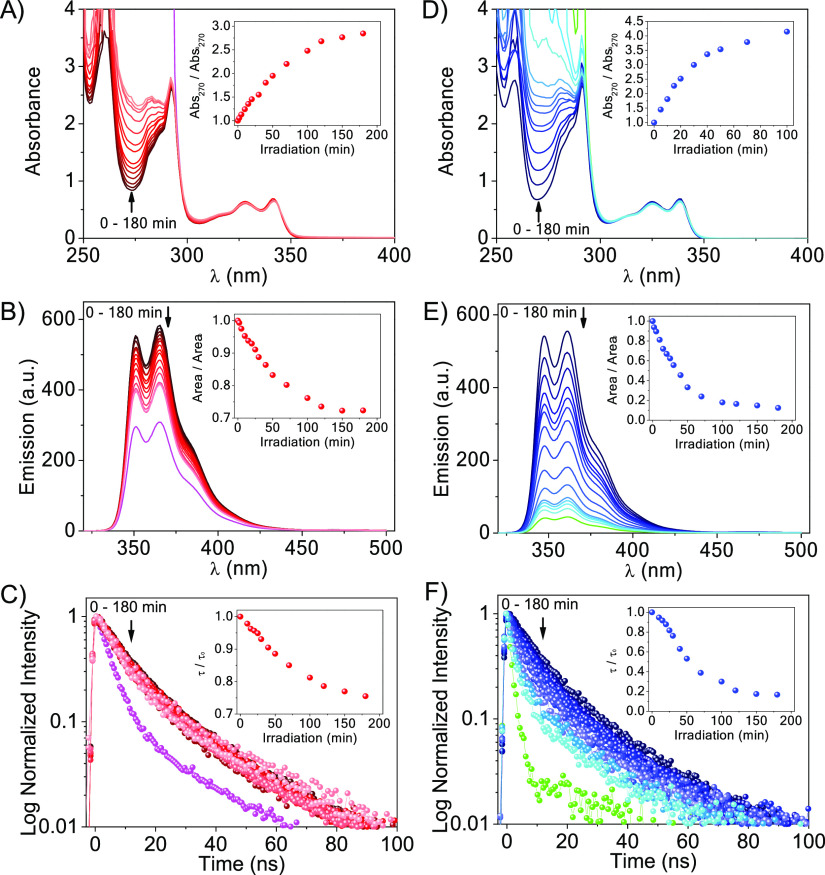
Kinetics of the evolution of **7** (left) and **8** (right) upon increasing irradiation times (λ_exc_ = 350 nm), at 0.2 mM in aerated 4CH_3_CN:1H_2_O. (A, D) Changes in the absorbance spectra (inset: relative absorbance changes); (B, E) changes in the steady-state emission spectra, λ_exc_ = 340 nm (inset: relative emission changes); (C, F) changes in the time-resolved emission, λ_exc_ = 340 nm (inset: relative lifetime changes). Pink (in A–C) and green (in D–F) traces correspond to **2** and **6**, respectively.

**Figure 5:**
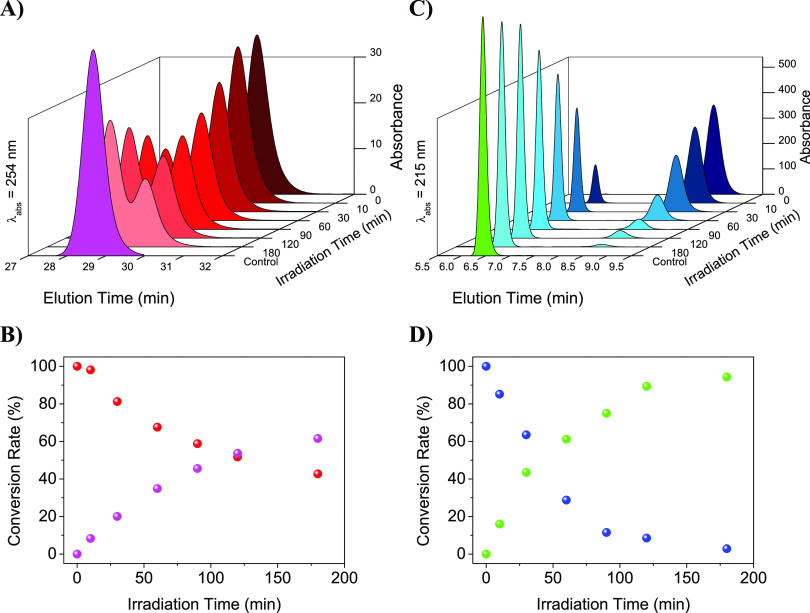
HPLC Chromatograms (top) and conversion rate (bottom) for **7** (left) or **8** (right) upon increasing irradiation times (λ_exc_ = 350 nm), at 0.2 mM in aerated 4CH_3_CN:1H_2_O. The chromatograms corresponding to **2** (pink trace, left) and **6** (green trace, right) are shown for comparison.

The photosensitized dimer repair was investigated upon selective irradiation of the Cbz chromophore (λ_max_ = 350 nm) in the two dimers **7** and **8**, by monitoring changes of UV–visible spectra, steady-state and time-resolved fluorescence spectroscopy, and HPLC analysis (Figures 4 and 5, top and bottom for **7** and **8**, respectively). Furthermore, to avoid subsequent formation of Thy<>Thy dimers, the prevalence of the Cbz singlet excited state (^1^Cbz*) was favored over the ^3^Cbz* by performing the experiments under air, although the Thy<>Thy competes with O_2_ for the ^1^Cbz* (see Figure S6.2 for quenching by O_2_, *k*_q*S*_ = 1.7 × 10^10^ M^–1^ s^–1^).

**Figure 6:**
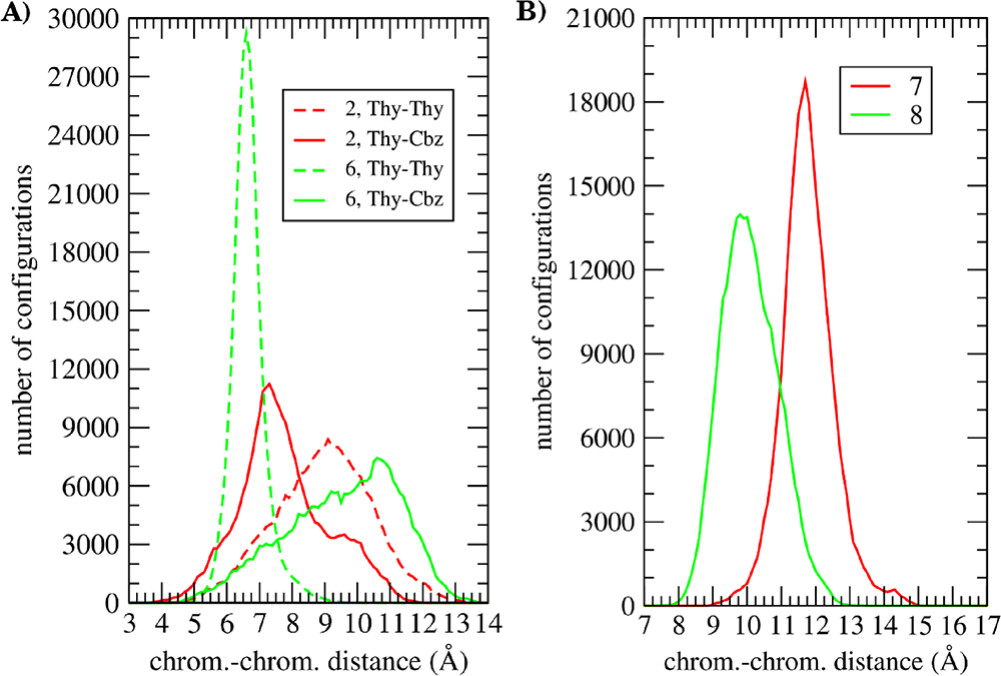
Left: Histogram of chromophore–chromophore distances of **2** and **6**. Right: Histogram of chromophore–chromophore Thy-Cbz distances of **7** and **8**. Results were in all cases obtained from molecular dynamics during 3 ns at 298 K in a 4:1 acetonitrile:water solvent. Each plot has been calculated using 300,000 configurations.

When aerated solutions of **7** and **8** were independently irradiated (λ_max_ = 350 nm), a remarkable increase in the absorbance at ∼260 nm was observed in both cases (Figure 4A,D, respectively). This increase could safely be attributed to the opening of the cyclobutane ring, giving rise to the two free Thy units. These changes were accompanied by a decrease in the fluorescence emission (steady-state and time-resolved, Figure 4B,C,E,F), which indicates that the free intramolecular Thy units have higher quenching capability of the singlet excited state of carbazole than the Thy<>Thy moieties. In fact, the efficiency of the TS intramolecular quenching of ^1^Cbz* by Thy at 12α can be determined from the lifetimes of ^1^Cbz* in **2** (pink trace in Figure 4C) and **6** (green trace in Figure 4F), and the lifetime of ^1^Cbz* under air (Figure S6.2). From the corresponding values of 4.8 ns for **2**, 1.7 ns for **6**, and 11.4 ns for ^1^Cbz*, the intramolecular TS quenching values in **2** and **6** are *k*_Sq_ = 1.2 × 10^8^ s^–1^ and 5.0 × 10^8^ s^–1^, respectively, much higher than the intermolecular quenching of ^1^Cbz* by Thy at the employed concentration (*k*_q*S*_ × [Thy] *ca*. 2.1 × 10^4^ s^–1^, see Figure S6.3).

This is not surprising since quenching of ^1^Cbz* by Thy<>Thy is likely happening TB since the probability of the three units being together TS is very low,^17^ while as soon as the Thy<>Thy are repaired, quenching of the Thy unit at 12α happens TS. Furthermore, the topology of the dimers plays again a role in the quenching of ^1^Cbz*, and therefore, in the efficiency of the photosensitized repair, with **8** being more reactive than **7**.

A further piece of evidence for the photosensitized Thy<>Thy repair was obtained by monitoring in parallel the evolution of the irradiation by HPLC (Figure 5A,C). Interestingly, in both cases, irradiation of **7** or **8** led to the opening of the cyclobutane ring; however, while for **8** the conversion was practically quantitative (Figure 5D), for **7**, it seems that a photoequilibrium was obtained, likely due to the concomitant photosensitized dimer formation (Figure 5B).

### 2.5 Computational Results

**Figure 7:**
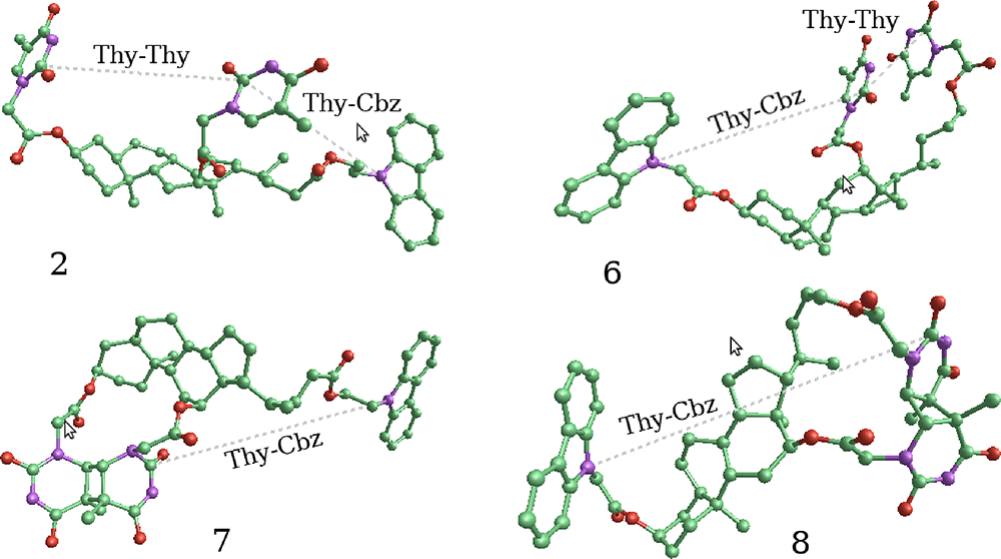
Configuration of molecules **2**, **6**, **7**, and **8** showing the distances between chromophore groups (dotted lines).

The participation of a TS mechanism in the formation of ^3^Thy* *vs* TB mechanism in the photosensitized repair was further investigated upon determining the chromophore–chromophore distances in compounds **2**, **6**, **7**, and **8** by using molecular dynamics at 298 K (see Section S7 and geometries file in the Supporting Information). Since in a previous study^18^ the effect of solvent was demonstrated to be crucial, here, the simulations include explicit solvent molecules, that is, a 4:1 mixture of acetonitrile:water. For each compound, 300,000 configurations were produced and their chromophore–chromophore distances employed to prepare the histograms are shown in Figure 6, and the configuration of the analyzed molecules is presented in Figure 7. The conformational analysis of **2** and **6** shows that the distances Cbz-Thy are lower than 10 Å (*ca.* 90% frequencies) in the case of **2**, while for **6**, only *ca*. 50% of dyads show distances <10 Å (Figure 6 left). These results support the TS-TTET proposed mechanism in the photosensitized dimerization and are in agreement with the higher reactivity observed for **2***vs***6** (see Figure 1). Moreover, the chromophore–chromophore (Thy<>Thy-Cbz) distances <10 Å in **7** and **8** have frequencies lower than 16% (Figure 6 right), in agreement with the likely TB mechanism operating for the photosensitized repair. Moreover, the higher speed of the photosensitized TB-repair found in the case of **8** compared to **7** (Figure 5) could be the result of a more favored overlap between the LUMO of the CBz* and the σ* of the spacer bonds.

Overall, opening of Thy<>Thy in **8** in aerated 4CH_3_CN:1H_2_O results quantitatively into **6**, due to the high distance between Thy-Cbz in **6** that prevents the subsequent TS-TTET in an aerated atmosphere. Conversely, opening of Thy<>Thy in **7** results into **2**, in which the low distance Cbz-Thy allows an efficient TS-TTET even under an aerated atmosphere. As a result of the opposite trends, different equilibrium compositions are found as observed in Figure 5, upon 180 min irradiation.

### 2.6 Mechanistic Proposal

**Scheme 3:**
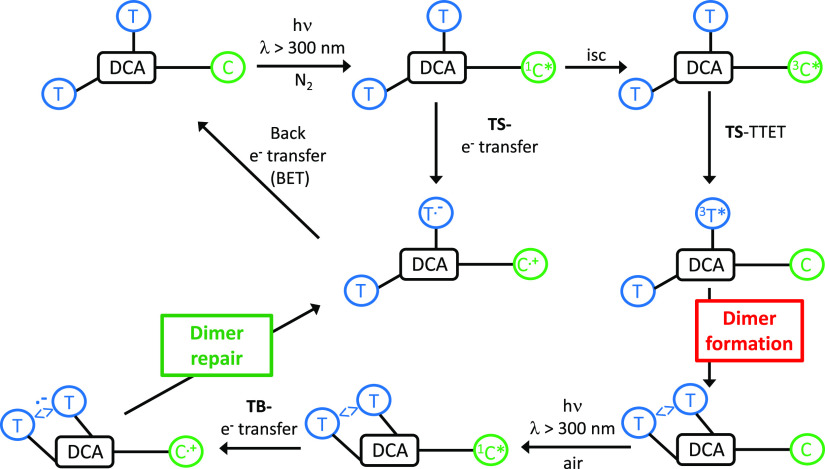
Postulated Mechanism to Explain Intramolecular Photosensitized Dimer Formation (TS Energy Transfer) and Repair (TB Electron Transfer)

We have demonstrated that Cbz covalently attached to the skeleton of DCA together with two Thy units can act as an efficient photosensitizer to produce Thy<>Thy dimers that could be repaired depending on the reaction conditions (Scheme 3). Thus, Cbz can be selectively excited, in the presence of Thy, to its singlet excited state, which is efficiently quenched by O_2_ and by TS electron transfer to the Thy unit in 12α. The thermodynamics of this electron transfer is favorable (*E*_Thy/Thy_^**·**–^ = −1.34 V,^38^*E*_Cbz_^**·**+^_/Cbz_ = 1.12 V *vs* SCE,^29^ and *E*1_Cbz*_ = 3.63 eV).^39^ Nevertheless, this pathway would be an energy-wasting channel, which regenerates the initial dyad upon back electron transfer. Still, the ^3^Cbz* is reached upon intersystem crossing. In the absence of oxygen, this excited species lives longer; thus, it is quenched by the Thy at 12α to give ^3^Thy* (TS-TTET is much more efficiently than TB-TTET). The ^3^Thy* gives rise to the Thy<>Thy upon [2 + 2] cycloaddition with the second intramolecular unit. The yield of the Thy<>Thy is enhanced in a deaerated atmosphere and is dependent on the topology of the three units.

Photosensitized repair starts with selective irradiation of Cbz in the presence of the Thy<>Thy. To enhance the prospects of the e^–^ transfer from the singlet, the experiments were performed under air. The electron transfer to the Thy<>Thy necessarily happens TB, since the probability of the three units being together is quite low. Once the e^–^ transfer has happened, opening of the dimer regenerates the initial systems. The formation of Thy<>Thy is disfavored in the presence of air. The calculated distances between chromophore units support the postulated mechanism.

## 3 Conclusions

In rigid bile acid-derived systems, TS triplet energy transfer from ^3^Cbz* to Thy gives rise to photosensitized Thy<>Thy formation. In general, when photorepair can also occur TS, the efficiency of this process *via* electron transfer from ^1^Cbz* to CPDs converts Thy<>Thy formation into a residual DNA photodamage. Conversely, we have demonstrated that if geometrical constraints prevent the dimer and the Cbz units from being close enough to each other for the electron transfer to happen TS, the repair should happen TB. This is a much less efficient mechanism, which results in enhanced prospects of Thy<>Thy photodamage.

## 4 Experimental Section

### 4.1 Chemicals

Deoxycholic acid (DCA), 9-carbazoleacetic acid (Cbz-CH_2_-COOH), 9*H*-carbazol-9-ethanol (Cbz-(CH_2_)_2_-OH), thymine 1-acetic acid (Thy-CH_2_-CO_2_H), benzyl bromide, 1,8-diazabicyclo[5.4.0]undec-7-ene (DBU), *N*,*N*-diisopropylethylamine (DIEA), 4-dimethylaminopyridine (4-DMAP), lithium aluminum hydride (LiAlH_4_), *O*-(benzotriazol-1-yl)-*N*,*N*,*N*,*N*′-tetramethyluronium tetrafluoroborate (TBTU), 2,4,6-trichlorobenzoyl chloride, benzyl alcohol, titanium(IV) isopropoxide, triethylamine, acetonitrile, dimethylformamide (DMF), and tetrahydrofuran (THF) were purchased from Sigma-Aldrich. Experimental procedures and methods for characterization are reported in the Supporting Information. Structural assignments were made with additional information from gCOSY, gHSQC, and gHMBC experiments. The assignment of hydrogen and carbon signals was based on a combination of 1D and 2D NMR experiments (^1^H; ^13^C; ^1^H,^1^H COSY; and ^1^H,^13^C HSQC).

### 4.2 Synthesis of **1**

To a stirred solution of Cbz-(CH_2_)_2_-OH (0.50 g, 2.25 mmol) and TBTU (0.87 g, 2.7 mmol) in anhydrous DMF (5 mL), **DCA** (0.93 g, 2.36 mmol) in anhydrous DMF (4 mL) followed by DIEA (1.17 mL, 6.75 mmol) were added dropwise, and then, the reaction mixture was allowed to react overnight at room temperature. Afterward, it was poured into brine and extracted with CH_2_Cl_2_; the combined organic layers were washed with brine, dried over MgSO_4_, and concentrated under reduced pressure. Purification by column chromatography (SiO_2_, EtOAc:hexane, 1:3) gave **1** as a white solid (0.93 g, 67%); ^1^H NMR (300 MHz, CDCl_3_) δ (ppm) 8.10 (d, *J* = 7.8 Hz, 2H, arom), 7.40–7.52 (m, 4H, arom), 7.20–7.32 (m, 2H, arom), 4.56 (m, 2H, CH_2_), 4.47 (m, 2H, CH_2_), 3.93 (*br* s, 1H, 12β-H), 3.61 (m, 1H, 3β-H), 0.91 (s, 3H, CH_3_), 0.87 (d, *J* = 6.3 Hz, 3H, 21-CH_3_), 0.80–2.29 (complex signal, 26H), 0.62 (s, 3H, CH_3_); ^13^C{1H} NMR (75 MHz, CDCl_3_) δ (ppm) 174.2 (C), 140.5 (2xC), 125.9 (2xCH), 123.1 (2xC), 120.4 (2xCH), 119.3 (2xCH), 108.7 (2xCH), 73.2 (CH), 71.8 (CH), 62.1 (CH_2_), 48.3 (CH), 47.3 (CH), 46.5 (C), 42.2 (CH), 41.8 (CH_2_), 36.5 (CH_2_), 36.1 (CH), 35.3 (CH_2_), 35.1 (CH), 34.2 (C), 33.7 (CH), 31.2 (CH_2_), 30.7 (CH_2_), 30.6 (CH_2_), 28.7 (CH_2_), 27.5 (CH_2_), 27.2 (CH_2_), 26.2 (CH_2_), 23.7 (CH_2_), 23.2 (CH_3_), 17.3 (CH_3_), 12.8 (CH_3_); HRMS (ESI) *m*/*z*: [M + H] + calcd for C_38_H_52_NO_4_ 586.3896; found, 586.3881.

### 4.3 Synthesis of **2**

A stirred suspension of Thy-CH_2_-COOH (0.42 g, 2.31 mmol) in anhydrous THF (10 mL) was treated with Et_3_N (0.64 mL) and 2,4,6-trichlorobenzoyl chloride (0.43 mL, 2.77 mmol), and the resulting mixture was allowed to react for 1.5 h. Then, a solution of 4-DMAP (0.11 g, 0.91 mmol) and **1** (0.45 g, 0.77 mmol) in anhydrous THF (10 mL) was added and stirred overnight. Afterward, the reaction mixture was poured into brine, extracted with CH_2_Cl_2_, and the combined extracts were washed with brine, dried over MgSO_4_, and concentrated under vacuum. Purification by column chromatography (SiO_2_, EtOAc:hexane, 9:1) gave **2** as a yellow oil (0.54 g, 77%); ^1^H NMR (300 MHz, CDCl_3_) δ (ppm) 10.36 (s, 1H, Thy-NH), 10.19 (s, 1H, Thy-NH), 8.10 (d, *J* = 7.8 Hz, 2H, arom), 7.39–7.56 (m, 4H, arom), 7.22–7.32 (m, 2H, arom), 6.98 (s, 1H, Thy-CH), 6.96 (s, 1H, Thy-CH), 5.11 (*br* s, 1H, 12β-H), 4.72 (m, 1H, 3β-H), 4.28–4.65 (m, 8H, 2xThy-CH_2_ + 2xCH_2_), 1.90 (*br* s, 3H, Thy-CH3), 1.86 (*br* s, 3H, Thy-CH_3_), 0.88 (s, 3H, CH_3_), 0.82–2.25 (complex signal, 26H), 0.72 (d, *J* = 5.7 Hz, 3H, 21-CH_3_), 0.65 (s, 3H, CH_3_); ^13^C{1H} NMR (75 MHz, CDCl_3_) δ (ppm) 173.9 (C), 167.2 (C), 166.5 (C), 164.7 (C), 164.5 (C), 151.5 (C), 150.8 (C), 140.7 (CH), 140.5 (CH), 140.3 (2xC), 125.8 (2xCH), 122.9 (2xC), 120.3 (2xCH), 119.2 (2xCH), 111.0 (C), 110.5 (C), 108.6 (2xCH), 77.7 (CH), 76.4 (CH), 62.1 (CH_2_), 49.2 (CH + 2xCH_2_), 47.2 (CH), 45.0 (C), 41.6 (CH_2_ + CH), 35.4 (CH), 34.5 (CH_2_), 34.5 (CH), 34.1 (CH), 34.0 (C), 31.7 (CH_2_), 30.9 (CH_2_), 30.4 (CH_2_), 27.1 (CH_2_), 26.7 (CH_2_), 25.9 (2xCH_2_), 25.2 (CH_2_), 23.3 (CH_2_), 22.8 (CH_3_), 17.5 (CH_3_), 12.3 (2xCH_3_), 12.0 (CH_3_); HRMS (ESI) *m*/*z*: [M + Na]^+^ calcd for C_52_H_63_N_5_O_10_Na 940.4473; found, 940.4497.

### 4.4 Synthesis of **3**

A stirred solution of **DCA** was converted into **DCA-Bn** following the procedure previously described in the literature.^8^ Then, a stirred suspension of LiAlH_4_ (0.33 g, 9.13 mmol) in anhydrous THF (8.5 mL) was cooled to −10 °C, treated with a solution of **DCA-Bn** (1.54 g, 3.19 mmol) in anhydrous THF (6 mL), and then the reaction mixture was refluxed overnight (70 °C). Afterward, the reaction was quenched with saturated aqueous NH_4_Cl solution (5 mL), redissolved with EtOAc, poured into aqueous HCl 1 M, and extracted with EtOAc. The combined organic layers were washed with brine, dried over MgSO_4_, and concentrated. Purification by column chromatography (SiO_2_, EtOAc) gave **3** as a colorless solid (1.05 g, 87%); ^1^H NMR (300 MHz, CDCl_3_) δ (ppm) 3.99 (*br* s, 1H, 12β-H), 3.61 (m, 3H, 3β-H + CH_2_), 0.98 (d, *J* = 6.3 Hz, 3H, 21-CH_3_), 0.93–1.89 (complex signal, 26H), 0.91 (s, 3H, CH_3_), 0.68 (s, 3H, CH_3_); ^13^C{1H} NMR (75 MHz, CDCl_3_) δ (ppm) 72.4 (CH), 71.0 (CH), 62.7 (CH_2_), 47.5 (CH), 46.8 (CH), 45.7 (C), 41.3 (CH), 35.6 (CH_2_), 35.2 (CH), 34.5 (CH), 34.4 (CH_2_), 33.3 (C), 32.9 (CH), 30.9 (CH_2_), 29.7 (CH_2_), 28.7 (CH_2_), 27.8 (CH_2_), 26.7 (CH_2_), 26.3 (CH_2_), 25.3 (CH_2_), 22.8 (CH_2_), 22.3 (CH_3_), 16.9 (CH_3_), 11.9 (CH_3_); HRMS (ESI) *m*/*z*: [M + H]^+^ calcd for C_24_H_43_O_3_ 379.3212; found, 379.3214.

### 4.5 Synthesis of **4**

TBTU (0.45 g, 1.41 mmol) and **3** (0.44 g, 1.17 mmol) were dissolved in anhydrous DMF (3 mL). Then, a solution of Thy-CH_2_CO_2_H (0.226 g, 1.23 mmol) in anhydrous DMF (2 mL) was added, followed by DIEA (0.61 mL, 3.51 mmol), and the resulting reaction mixture was allowed to react at rt for 7 h. Then, it was poured into brine and extracted with CH_2_Cl_2_. The combined organic extracts were washed with brine, dried over MgSO_4_, and concentrated under reduced pressure. Purification by column chromatography (SiO_2_, CH_2_Cl_2_:MeOH, 5:0.2) gave **4** as a yellowish solid (0.353 g, 56%); ^1^H NMR (300 MHz, CDCl_3_) δ (ppm) 10.01 (*br* s, 1H, Thy-NH), 6.96 (s, 1H, Thy-CH), 4.41 (s, 2H, Thy-CH_2_), 4.10 (m, 2H, CH_2_), 3.94 (*br* s, 1H, 12β-H), 3.57 (m, 1H, 3β-H), 2.76 (*br* s, 1H, OH), 2.52 (*br* s, 1H, OH), 1.88 (d, *J* = 1.2 Hz, 3H, Thy-CH_3_), 0.92 (d, *J* = 6.6 Hz, 3H, 21-CH_3_), 0.86 (s, 3H, CH_3_), 0.82–1.90 (complex signal, 26H), 0.63 (s, 3H, CH_3_); ^13^C{1H} NMR (75 MHz, CDCl_3_) δ (ppm) 167.8 (C), 164.6 (C), 151.2 (C), 140.4 (CH), 111.2 (C), 73.2 (CH), 71.7 (CH), 66.7 (CH_2_), 48.8 (CH_2_), 48.3 (CH), 47.3 (CH), 46.5 (C), 42.1 (CH), 36.3 (CH_2_), 36.0 (CH), 35.4 (CH_2_), 35.3 (CH), 34.2 (C), 33.5 (CH), 31.8 (CH_2_), 30.3 (CH_2_), 28.6 (CH_2_), 27.7 (CH_2_), 27.2 (CH_2_), 26.2 (CH_2_), 25.2 (CH_2_), 23.8 (CH_2_), 23.1 (CH_3_), 17.5 (CH_3_), 12.8 (CH_3_), 12.4 (CH_3_); HRMS (ESI) *m*/*z*: [M + H]^+^ calcd for C_31_H_49_N_2_O_6_ 545.3591; found, 545.3599.

### 4.6 Synthesis of **5**

To a stirred solution of **4** (0.55 g, 1.01 mmol) and TBTU (0.40 g, 1.21 mmol) in anhydrous DMF (2 mL), a solution of Cbz-CH_2_-COOH (0.25 g, 1.11 mmol) in anhydrous DMF (3 mL) followed by DIEA (0.53 mL, 3.03 mmol) were added dropwise, and then, the reaction mixture was allowed to react overnight at rt. Afterward, it was poured into brine and extracted with CH_2_Cl_2_; the combined organic layers were washed with brine, dried over MgSO_4_, and concentrated under reduced pressure. Purification by column chromatography (SiO_2_, EtOAc:hexane, 1:1) gave **5** as a white solid (0.33 g, 43%); ^1^H NMR (300 MHz, CDCl_3_) δ (ppm) 8.98 (s, 1H, Thy-NH), 8.09 (d, *J* = 7.8 Hz, 2H, arom), 7.42–7.52 (m, 2H, arom), 7.20–7.35 (m, 4H, arom), 6.88 (s, 1H, Thy-CH), 4.96 (s, 2H, Cbz-CH_2_), 4.79 (*br* s, 1H, 3β-H), 4.39 (s, 2H, Thy-CH_2_), 4.16 (m, 2H, CH_2_), 3.97 (*br* s, 1H, 12β-H), 1.91 (d, *J* = 1.2 Hz, 3H, Thy-CH_3_), 0.96 (d, *J* = 6.3 Hz, 3H, 21-CH_3_), 0.90 (s, 3H, CH_3_), 0.79–1.97 (complex signal, 26H), 0.67 (s, 3H, CH_3_); ^13^C{1H} NMR (75 MHz, CDCl_3_) δ (ppm) 168.2 (C), 167.7 (C), 164.1 (C), 150.9 (C), 140.8 (2xC), 140.3 (CH), 126.1 (2xCH), 123.4 (2xC), 120.6 (2xCH), 119.7 (2xCH), 111.4 (C), 108.6 (2xCH), 76.2 (CH), 73.3 (CH), 66.8 (CH_2_), 48.8 (CH_2_), 48.4 (CH), 47.6 (CH), 46.6 (C), 45.2 (CH_2_), 42.0 (CH), 36.1 (CH), 35.3 (CH), 34.9 (CH_2_), 34.3 (C), 33.8 (CH), 32.2 (CH_2_), 31.9 (CH_2_), 28.8 (CH_2_), 27.7 (CH_2_), 27.0 (CH_2_), 26.7 (CH_2_), 26.1 (CH_2_), 25.3 (CH_2_), 23.8 (CH_2_), 23.2 (CH_3_), 17.7 (CH_3_), 12.9 (CH_3_), 12.5 (CH_3_); HRMS (ESI) *m*/*z*: [M + H]^+^ calcd for C_45_H_58_N_3_O_7_ 752.4300; found, 752.4308.

### 4.7 Synthesis of **6**

A stirred suspension of Thy-CH_2_-COOH (0.50 g, 2.72 mmol) in anhydrous THF (15 mL) was treated with Et_3_N (0.76 mL) and 2,4,6-trichlorobenzoyl chloride (0.51 mL, 3.29 mmol), and the resulting mixture was allowed to react for 90 min. Then, a solution of 4-DMAP (0.064 g, 0.53 mmol) and **5** (0.34 g, 0.45 mmol) in anhydrous THF (11 mL) was added and stirred overnight. Afterward, the reaction mixture was poured into brine, extracted with CH_2_Cl_2_, and the combined extracts were washed with brine, dried over MgSO_4_, and concentrated under vacuum. Purification by column chromatography (SiO_2_, EtOAc:hexane, 1:1) gave **6** as a colorless oil (0.25 g, 60%); ^1^H NMR (300 MHz, CDCl_3_) δ (ppm) 11.07 (s, 1H, Thy-NH), 11.02 (s, 1H, Thy-NH), 8.00 (d, *J* = 7.8 Hz, 2H, arom), 7.42–7.55 (m, 2H, arom), 7.33–7.39 (d, *J* = 8.4 Hz, 2H, arom), 7.21–7.30 (m, 2H, arom), 6.99 (*br* d, *J* = 1.2 Hz, 1H, Thy-CH), 5.53 (*br* d, *J* = 1.2 Hz, 1H, Thy-CH), 5.12 (d, *J* = 17.7 Hz, 1H, Cbz-CH_2_), 4.97 (d, *J* = 17.7 Hz, 1H, Cbz-CH_2_), 4.96 (*br* s, 1H, 12β-H), 4.90 (m, 1H, 3β-H), 4.55 (d, *J* = 16.8 Hz, 1H, Thy-CH_2_), 4.49 (d, *J* = 17.4 Hz, 1H, Thy-CH_2_), 4.23 (d, *J* = 16.8 Hz, 1H, Thy-CH_2_), 4.19 (m, 2H, CH_2_), 2.96 (d, *J* = 17.4 Hz, 1H, Thy-CH_2_), 1.95 (d, *J* = 1.2 Hz, 3H, Thy-CH_3_), 1.12 (*br* s, 3H, Thy-CH_3_), 0.83 (s, 3H, CH_3_), 0.71 (d, *J* = 4.8 Hz, 3H, 21-CH_3_), 0.59 (s, 3H, CH_3_), 0.50–1.79 (complex signal, 26H); ^13^C{1H} NMR (75 MHz, CDCl_3_) δ (ppm) 168.3 (C), 167.4 (C), 165.9 (C), 165.0 (C), 164.5 (C), 152.4 (C), 151.4 (C), 140.4 (2xC), 140.2 (CH), 140.0 (CH), 126.3 (2xCH), 123.0 (2xC), 120.5 (2xCH), 120.2 (2xCH), 111.7 (C), 110.6 (C), 108.3 (2xCH), 77.9 (CH), 75.8 (CH), 66.7 (CH_2_), 50.3 (CH_2_), 50.0 (CH_2_), 49.9 (CH), 47.9 (CH), 45.5 (CH_2_), 44.9 (C), 41.4 (CH), 35.8 (CH), 35.1 (CH), 34.5 (CH_2_), 33.7 (CH), 33.5 (C), 32.0 (CH_2_), 31.3 (CH_2_), 27.2 (CH_2_), 26.5 (CH_2_), 26.4 (CH_2_), 26.3 (CH_2_), 26.0 (CH_2_), 24.4 (CH_2_), 23.1 (CH_2_), 22.5 (CH_3_), 17.4 (CH_3_), 12.5 (CH_3_), 12.2 (CH_3_), 12.0 (CH_3_); HRMS (ESI) *m*/*z*: [M + H]^+^ calcd for C_52_H_64_N_5_O_10_ 918.4653; found, 918.4673.

### 4.8 Photosensitized Preparation of **7**

A solution of **2** (0.267 g, 0.27 mmol) in CH_3_CN (350 mL), placed in a Pyrex round-bottom flask, was purged with N_2_ and irradiated in a photoreactor using 8 lamps (λ_max_ = 350 nm) for 4 h. Then, the solvent was concentrated under vacuum and product **7** was obtained pure without further purification. ^1^H NMR (400 MHz, C_5_D_5_N) δ (ppm) 13.24 (*br* s, 1H, Thy-NH), 13.18 (*br* s, 1H, Thy-NH), 8.27 (d, *J* = 7.6 Hz, 2H, arom), 7.55–7.65 (m, 4H, arom), 7.34–7.40 (m, 2H, arom), 5.34 (s, 1H, 12β-H), 5.08 (m, 1H, 3β-H), 4.95 (m, 1H, Thy-CH_2_), 4.84 (d, *J* = 16.0 Hz, 1H, Thy-CH_2_), 4.55–4.61 (m, 3H, CH_2_ + Thy<>Thy-CH), 4.40–4.54 (m, 2H, CH_2_), 4.27 (*br* d, *J* = 16.0 Hz, 1H, Thy-CH_2_), 4.13 (d, *J* = 2.8 Hz, 1H, Thy<>Thy-CH), 4.06 (d, *J* = 16.0 Hz, 1H, Thy-CH_2_), 2.14 (s, 3H, Thy-CH_3_), 1.87 (s, 3H, Thy-CH_3_), 0.99 (d, *J* = 6.0 Hz, 3H), 0.78–2.40 (complex signal, 26H), 0.77 (s, 3H), 0.60 (s, 3H); ^13^C{1H} NMR (100 MHz, C_5_D_5_N) δ (ppm) 173.9 (C), 170.9 (C), 170.8 (C), 168.5 (C), 168.2 (C), 152.7 (C), 152.6 (C), 141.5 (2xC), 126.8 (2xCH), 123.9 (2xC), 121.2 (2xCH), 120.1 (2xCH), 110.0 (2xCH), 78.7 (CH), 73.7 (CH), 66.4 (CH), 64.6 (CH), 62.9 (CH_2_), 52.9 (CH), 52.4 (CH_2_), 50.5 (CH_2_), 48.3 (CH), 46.0 (C), 45.9 (C), 45.7 (C), 42.5 (CH_2_), 40.4 (CH), 35.9 (CH), 35.0 (CH), 34.5 (CH_2_), 33.7 (CH), 32.9 (C), 31.9 (CH_2_), 31.0 (CH_2_), 30.2 (CH_2_), 27.8 (CH_2_), 26.3 (2xCH_2_), 25.6 (CH_2_), 24.3 (CH_2_), 23.7 (CH_3_), 23.6 (CH_2_), 22.8 (CH_3_), 21.9 (CH_3_), 17.5 (CH_3_), 12.9 (CH_3_); HRMS (ESI) *m*/*z*: [M + H]^+^ calcd for C_52_H_64_N_5_O_10_ 918.4653; found, 918.4662.

### 4.9 Photosensitized Preparation of **8**

A solution of **6** (0.155 g, 0.17 mmol) in CH_3_CN (200 mL), placed in a Pyrex round-bottom flask, was purged with N_2_ and irradiated in a photoreactor using 8 lamps (λ_max_ = 350 nm) for 16 h. Then, the solvent was concentrated under vacuum and the crude was purified by column chromatography (SiO_2_, EtOAc:hexane, 3:2 to give **8** (0.105 g, 68%); ^1^H NMR (300 MHz, CDCl_3_) δ (ppm) 8.04–8.10 (m, 3H, arom + Thy-NH), 7.50 (s, 1H, Thy-NH), 7.39–7.46 (m, 2H, arom), 7.32 (d, *J* = 8.1 Hz, 2H, arom), 7.18–7.26 (m, 2H, arom), 5.09 (*br* s, 1H, 12β-H), 4.99 (d, *J* = 17.4 Hz, 1H, Cbz-CH_2_), 4.92 (d, *J* = 17.4 Hz, 1H, Cbz-CH_2_), 4.76 (m, 1H, 3β-H), 4.90 (m, 1H), 4.33 (d, *J* = 16.8 Hz, 1H, Thy-CH_2_), 4.20 (d, *J* = 16.2 Hz, 1H, Thy-CH_2_), 4.11 (d, *J* = 6.3 Hz, 1H, Thy<>Thy-CH), 4.07 (d, *J* = 16.8 Hz, 1H, Thy-CH_2_), 4.02 (d, *J* = 6.3 Hz, 1H, Thy<>Thy-CH), 3.88 (d, *J* = 16.2 Hz, 1H, Thy-CH_2_), 3.82 (m, 1H), 1.55 (s, 3H, Thy-CH_3_), 1.50 (s, 3H, Thy-CH_3_), 0.89 (s, 3H, CH_3_), 0.86 (s, 3H, CH_3_), 0.82–1.91 (complex signal, 26H), 0.78 (s, 3H, CH_3_); ^13^C{1H} NMR (75 MHz, CDCl_3_) δ (ppm) 171.2 (C), 170.8 (C), 169.4 (2xC), 168.3 (C), 151.3 (C), 150.3 (C), 140.8 (2xC), 125.9 (2xCH), 123.3 (2xC), 120.5 (2xCH), 119.6 (2xCH), 108.6 (2xCH), 78.2 (CH), 76.7 (CH), 66.6 (CH_2_), 66.3 (CH), 65.9 (CH), 50.9 (CH_2_), 50.3 (CH_2_), 49.2 (CH), 46.8 (C), 46.7 (CH), 46.2 (C), 45.5 (C), 45.3 (CH_2_), 42.2 (CH), 36.5 (CH), 35.6 (CH), 35.0 (CH_2_), 34.8 (CH), 34.6 (C), 33.7 (CH_2_), 32.7 (CH_2_), 27.4 (CH_2_), 27.0 (CH_2_), 26.6 (CH_2_), 26.4 (CH_2_), 25.9 (CH_2_), 25.6 (CH_2_), 23.5 (CH_2_), 23.4 (CH_3_), 22.3 (CH_3_), 22.2 (CH_3_), 16.4 (CH_3_), 14.4 (CH_3_); HRMS (ESI) *m*/*z*: [M + H]^+^ calcd for C_52_H_64_N_5_O_10_ 918.4653; found, 918.4662.

## Supporting Information Available

^1^H NMR, and ^13^C{1H} NMR and bidimensional spectra, X-ray details, additional photophysical experiments, computational methods (PDF)
Geometries: Coordinates of all species calculated in Figure 7 (MOV)
Videos of the crystal structure of **8** and computational structures of molecules **2**, **6**, **7**, and **8** (ZIP)

## Supplementary Material

jo2c00942_si_001.pdf

jo2c00942_si_002.mov
