# Supplementary material for: Topology and Excited State Multiplicity as Controlling Factors in the Carbazole-Photosensitized CPD Formation and Repair
Source: J Org Chem. 2022 Aug 18;87(17):11433–42. doi: 10.1021/acs.joc.2c00942 (PMC9447287; doi:10.1021/acs.joc.2c00942)
Supplement: Supplementary file 1 — jo2c00942_si_001.pdf [file jo2c00942_si_001.pdf]

Supporting Information

Topology and Excited State Multiplicity as Controlling Factors in the Carbazole-photosensitized CPDs Formation and Repair

Gemma M. Rodriguez-Muñiz, [a] Miguel Gomez-Mendoza,[a,b] Paula Miro,[a] Pilar García-Orduña,[c] German Sastre,[a] Miguel A. Miranda*[a] and M. Luisa Marin*[a]

[a] Instituto de Tecnología Química, Universitat Politècnica de València-Consejo Superior de Investigaciones Científicas, Avenida de los Naranjos s/n, 46022 Valencia, Spain.
[b] Present address: Photoactivated Processes Unit, IMDEA Energy Institute, Avda Ramon de la Sagra 3, 28935 Mostoles, Madrid, Spain.
[c] Dpto. Química Inorgánica, ISQCH-Instituto de Síntesis Química y Catálisis Homogénea, Facultad de Ciencias, CSIC-Universidad de Zaragoza, Pedro Cerbuna 12, 50009 Zaragoza, Spain.

*Corresponding authors

E-mail addresses: marmarin@qim.upv.es; mmiranda@qim.upv.es

Table of contents

S1. Experimental procedures S3

S2. Characterization S5

S2.1. ^{1}H, $^{13}C\{1H\}$, $^{13}C\{1H\}$ DEPT-135 and ^{1}H-^{13}C HSQC NMR of **1** S5

S2.2. ^{1}H, $^{13}C\{1H\}$, $^{13}C\{1H\}$ DEPT-135 and ^{1}H-^{13}C HSQC NMR of **2** S8

S2.3. ^{1}H and $^{13}C\{1H\}$ NMR of **3** S11

S2.4. ^{1}H, $^{13}C\{1H\}$, $^{13}C\{1H\}$ DEPT-135 and ^{1}H-^{13}C HSQC NMR of **4** S13

S2.5. ^{1}H, $^{13}C\{1H\}$, $^{13}C\{1H\}$ DEPT-135 and ^{1}H-^{13}C HSQC NMR of **5** S16

S2.6. ^{1}H, $^{13}C\{1H\}$, $^{13}C\{1H\}$ DEPT-135 and ^{1}H-^{13}C HSQC NMR of **6** S19

S2.7. ^{1}H, $^{13}C\{1H\}$, $^{13}C\{1H\}$ DEPT-135, ^{1}H-^{13}C HSQC, ^{1}H-^{1}H COSY and ^{1}H-^{1}H NOESY NMR of **7** S22

S2.8. ^{1}H, $^{13}C\{1H\}$, $^{13}C\{1H\}$ DEPT-135, ^{1}H-^{13}C HSQC, ^{1}H-^{1}H COSY and ^{1}H-^{1}H NOESY NMR of **8** S26

S3. X Rays S30

S3.1. Experimental S30

S3.2. References S30

S3.3. Characterization S31

S4. Synthesis S34

S4.1. Synthesis and characterization of **9** S34

S4.2. ^{1}H, $^{13}C\{1H\}$, $^{13}C\{1H\}$ DEPT-135, ^{1}H-^{13}C HSQC, ^{1}H-^{1}H COSY and ^{1}H-^{1}H NOESY NMR of **9** S35

S4.3. Synthesis and characterization of **10** S39

S4.4. ^{1}H and $^{13}C\{1H\}$ NMR of **10** S40

S4.5. Synthesis and characterization of **11** S42

S4.6. ^{1}H and $^{13}C\{1H\}$ NMR of **11** S43

S5. Control experiments in the photosensitized Thy<>Thy dimers formation S45

S6. Control experiments in the photosensitized Thy<>Thy repair S47

S7. Computational methodology S49

S7.1. References S51

S1. Experimental procedures

Nuclear Magnetic Resonance (NMR)

A Bruker 400 MHz and a Bruker 300 MHz spectrometers were used for the NMR experiments. The signal of the solvent, chloroform or pyridine was used as a reference for the determination of the chemical shifts (δ) in ppm.

UPLC-MS-MS

Chromatography was performed on an ACQUITY UPLC system (Waters Corp.) containing a conditioned autosampler at 4 °C. The separation was carried out on an ACQUITY UPLC BEH C18 column (50 mm × 2.1 mm i.d., 1.7 μm) at the temperature of 40 °C. For the HRMS measurements, the Waters ACQUITY™ XevoQToF Spectrometer (Waters Corp.) was connected to the UPLC system via an electrospray ionization (ESI) interface. The ESI source was operated in positive or negative ionization mode depending on the experiment with the capillary voltage at 3.0 kV. The temperature of the source and desolvation was set at 120 °C and 500 °C, respectively. All data collected in Centroid mode were acquired using Masslynx™ software (Waters Corp.). Leucine-enkephalin was used as the lock mass generating an [M+H]+ ion (m/z 556.2771) at a concentration of 500 pg/mL and flow rate of 20 μL/min to ensure accuracy during the MS analysis.

Photophysical equipment

A Shimadzu UV-2101PC spectrophotometer was employed for the UV/Vis absorption spectra.

Steady-state and time-resolved fluorescence experiments were performed with a Photon Technology International (PTI) LPS-220B spectrofluorometer and with a EasyLife V spectrofluorometer from OBB, respectively. In the case of time-resolved fluorescence, the excitation source was equipped with a pulsed LED (λ_{exc}=340 nm); residual excitation signal was filtered in emission by using a cut-off filter (50% transmission at 375 nm). Monoexponential decay functions that use a deconvolution procedure to separate them from the lamp pulse profile provided the fitted kinetic traces.

An excimer laser of XeCl (LEXTRA 50 Lambda Physik Laser Technik) was employed for the laser flash photolysis (LFP) experiments carry out at the excitation wavelength of 308 nm (single pulses were ca. 10 ns duration, and energy was 15 mJ/pulse). A pulsed Nd: YAG SL404G-10 Spectron Laser Systems was employed to carry out the LFP experiments at the excitation wavelength of 266 nm (~10 ns duration and energy lower than xx mJ pulse^{-1}). The laser flash photolysis system is formed by the pulsed laser, a pulsed Lo255 Oriel Xenon lamp, a 77200 Oriel monochromator, an Oriel photomultiplier tube (PMT) housing, a 70705 PMT power supply and a TDS-640A Tektronix oscilloscope. A customized Luzchem Research LFP-111 system was employed to collect and process the data.

Dimers formation steady-state kinetics

Irradiation of solutions of **2** or **6** in $4CH_3CN:1H_2O$ at the concentration of 4.4 x 10^{-5} M under N_2 atmosphere were performed in the Luzchem photoreactor with lamps centred at 350 nm with a pyrex filter, and monitored by UV-Vis kinetic analysis.

Photorepair steady-state kinetics

Aerated solutions (2 x 10^{-4} M in $4CH_3CN:1H_2O$) of **7** or **8** were irradiated using the Luzchem photoreactor equipped with four lamps centred at 350 nm with a pyrex filter and continuous stirring. Kinetics were monitored by UV-Vis, steady-state and time resolved fluorescence and HPLC analysis.

The HPLC was an Agilent 1100 Series model with quaternary pump G1311A, photodiode detector VWD G1314A, standard liquid autosampler G1313A and degasser G1322A. A Mediterranea Sea 18 column (25 cm × 0.46 cm, 5 μm particle size) was employed. The kinetics of the irradiation of **7** or **8** was achieved with isocratic elution using 30% H_2O (pH=3) and 70% CH_3CN or 10% H_2O (pH=3) and 90% CH_3CN, respectively, as the mobile phase, working at 1.5 mL/min. The absorption was detected at 254 and 215 nm, respectively. The injection volume was 30 μL.

Fluorescence quenching experiments

The concentration of commercial Cbz-CH_2CH_2OH solution for the fluorescence experiments was 50 μM at the excitation wavelength (λ_{exc} = 340 nm). In a typical quenching experiment, the appropriate volumes of a freshly prepared ThyCH_2CO_2H solution were added to the Cbz-CH_2CH_2OH in aerated solutions (up to 35 mM).

Laser flash photolysis experiments

Transient spectra were recorded upon laser flash photolysis excitation at 308 nm, using N_2-purged $4CH_3CN:1H_2O$ solutions (9 x 10^{-5} M). The decays were recorded at 420 nm. Photophysical measurements were run at room temperature, using quartz cells of 1 cm optical path length.

S2. Characterization

S2.1. ^{1}H, ^{13}C{1H}, ^{13}C{1H} DEPT-135 and ^{1}H-^{13}C HSQC NMR of **1**

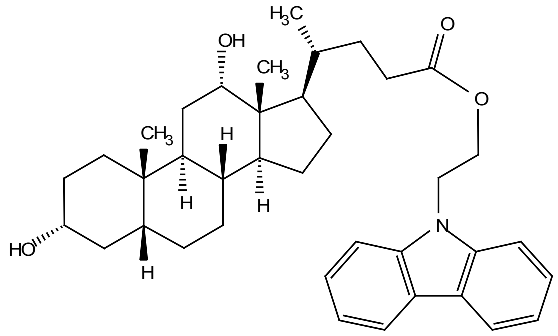

^{1}H NMR (300 MHz, CDCl$_3$)

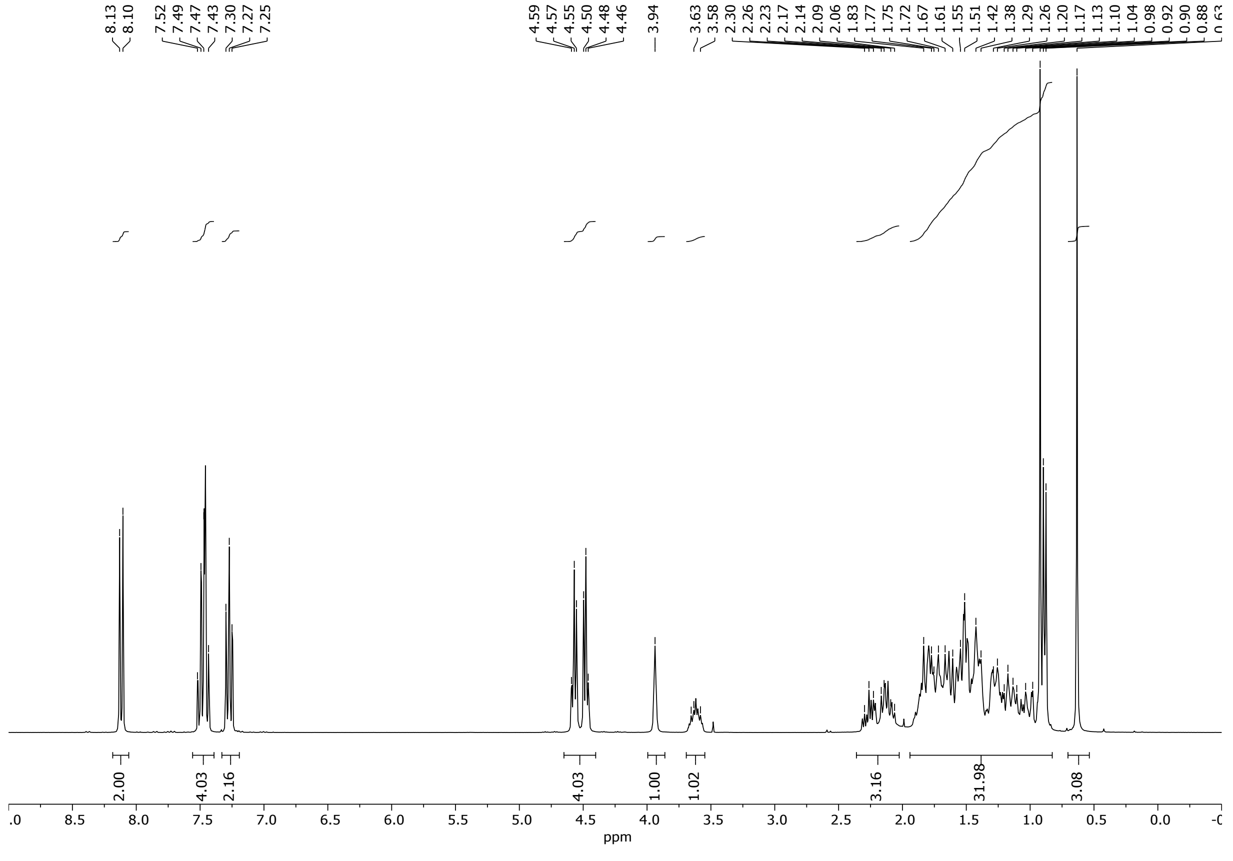

$^{13}C\{1H\}$ NMR (75 MHz, $CDCl_3$)

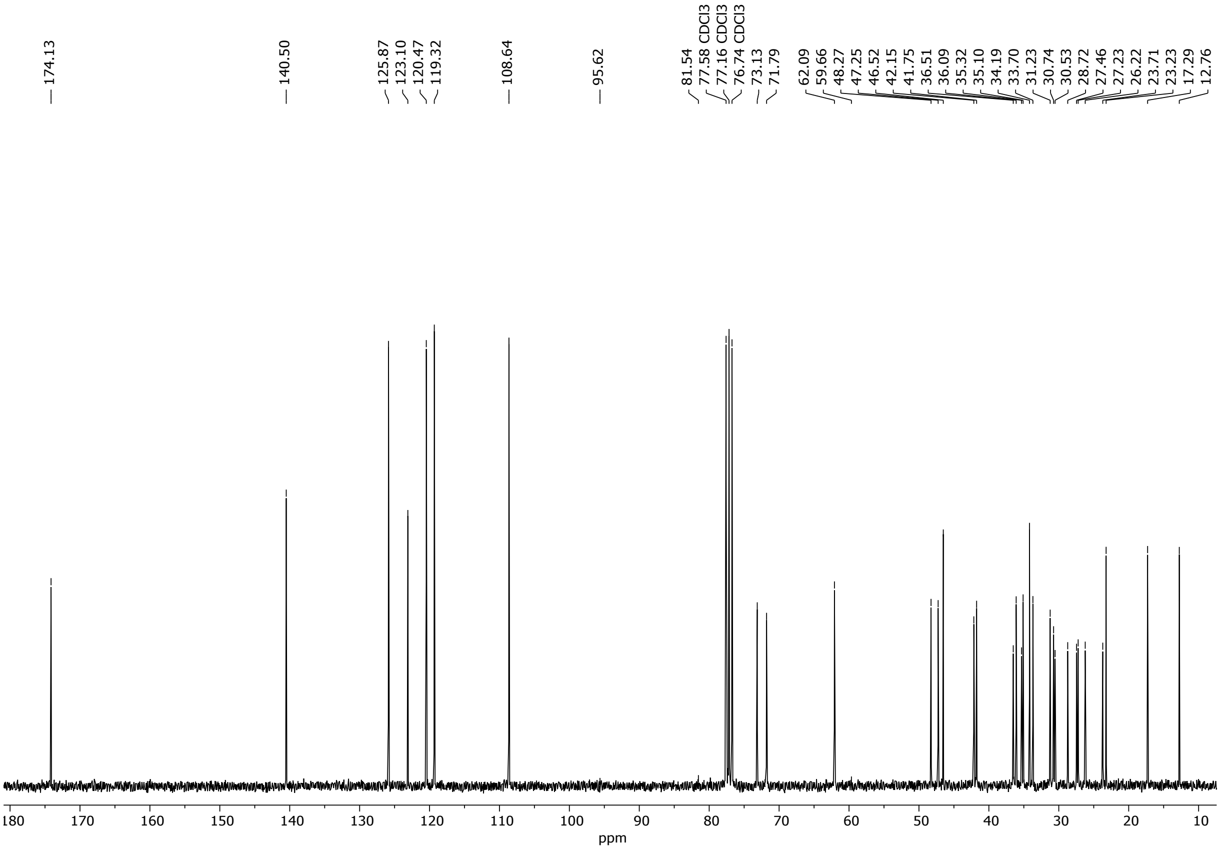

$^{13}C\{1H\}$ DEPT-135 NMR (75 MHz, $CDCl_3$)

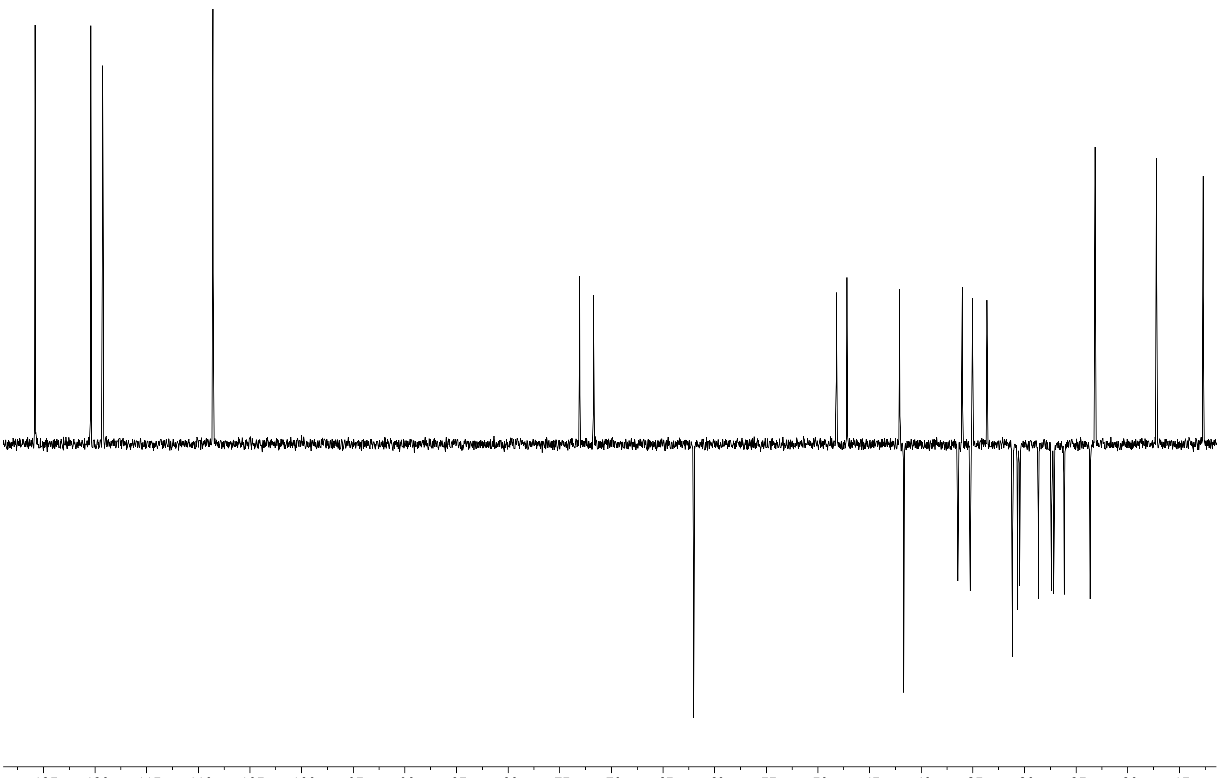

^{1}H-^{13}C HSQC NMR ($CDCl_3$)

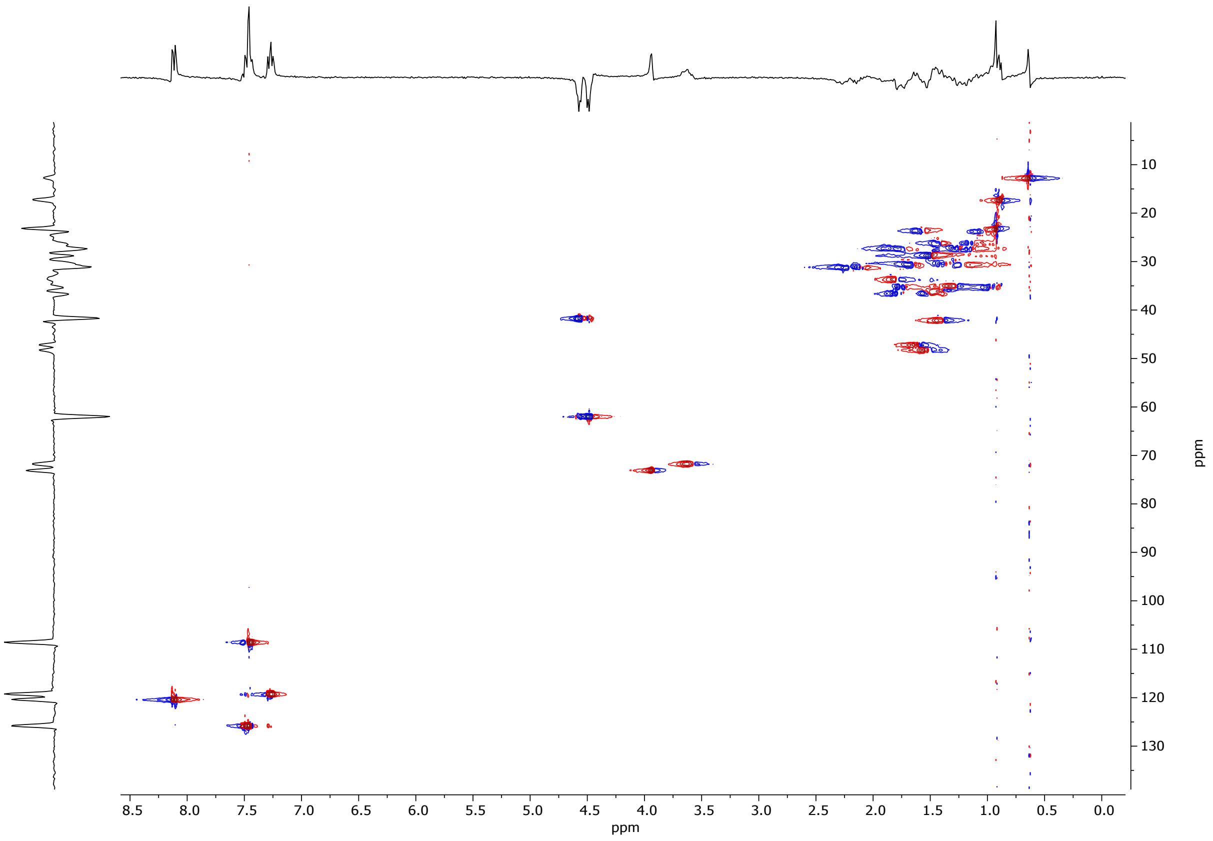

S2.2. ^{1}H, ^{13}C{1H}, ^{13}C{1H} DEPT-135 and ^{1}H-^{13}C HSQC NMR of **2**

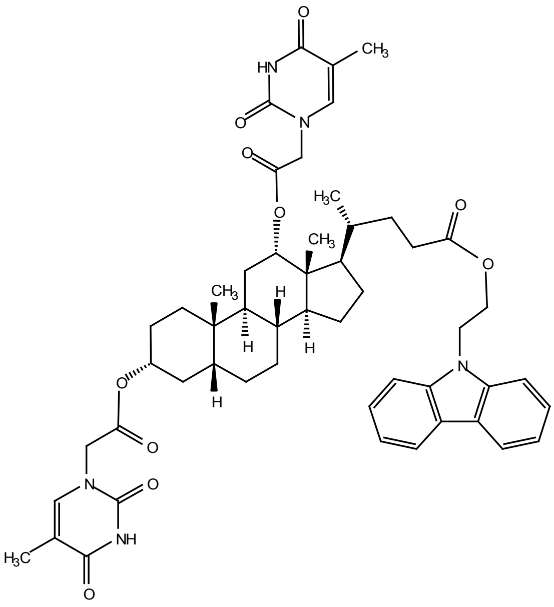

^{1}H NMR (300 MHz, $CDCl_3$)

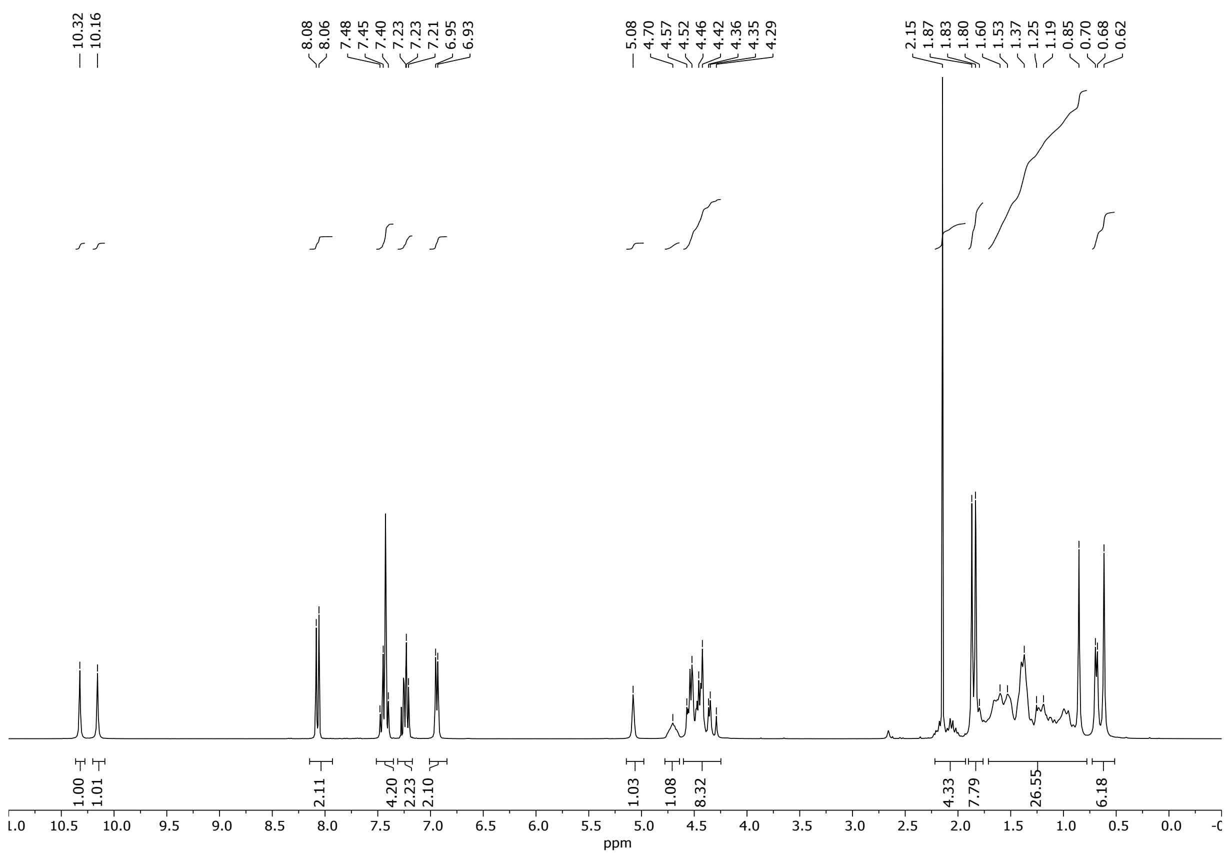

$^{13}C\{1H\}$ NMR (75 MHz, $CDCl_3$)

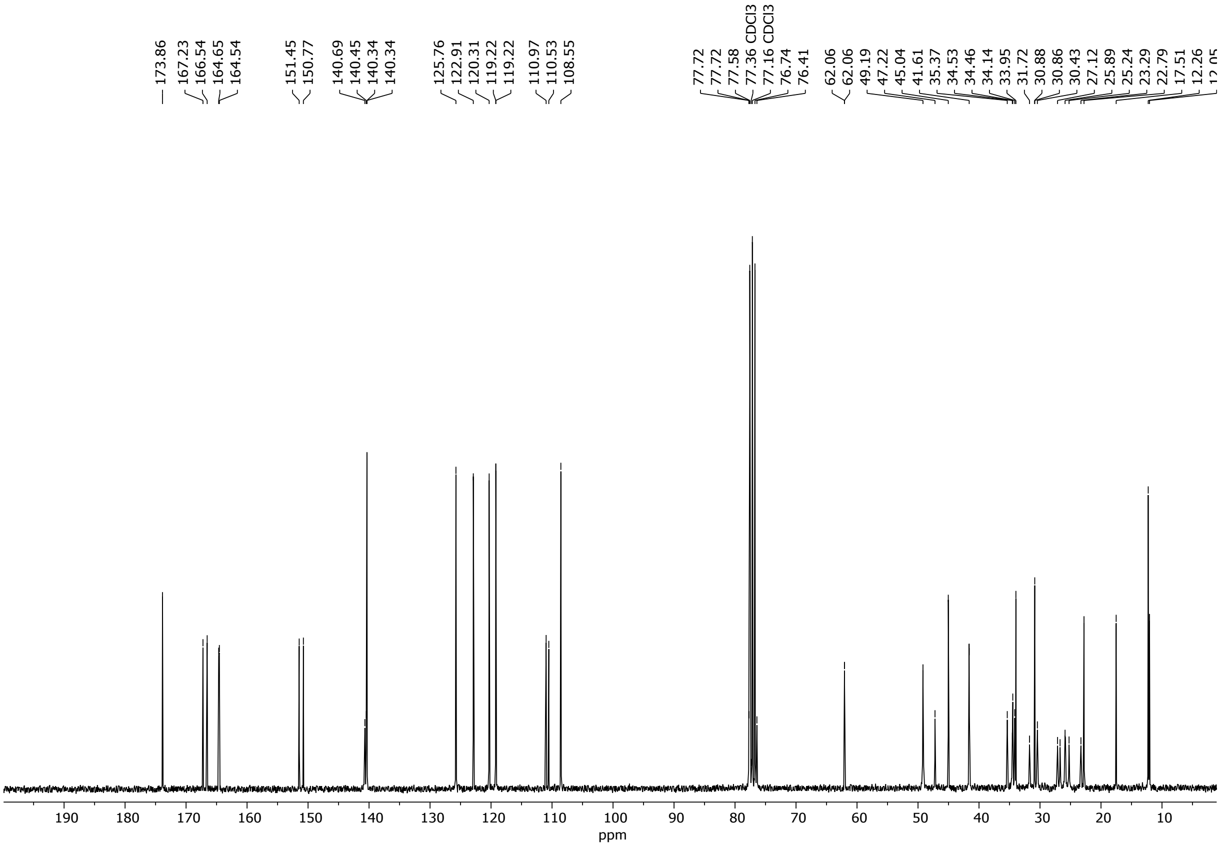

$^{13}C\{1H\}$ DEPT-135 (75 MHz, $CDCl_3$)

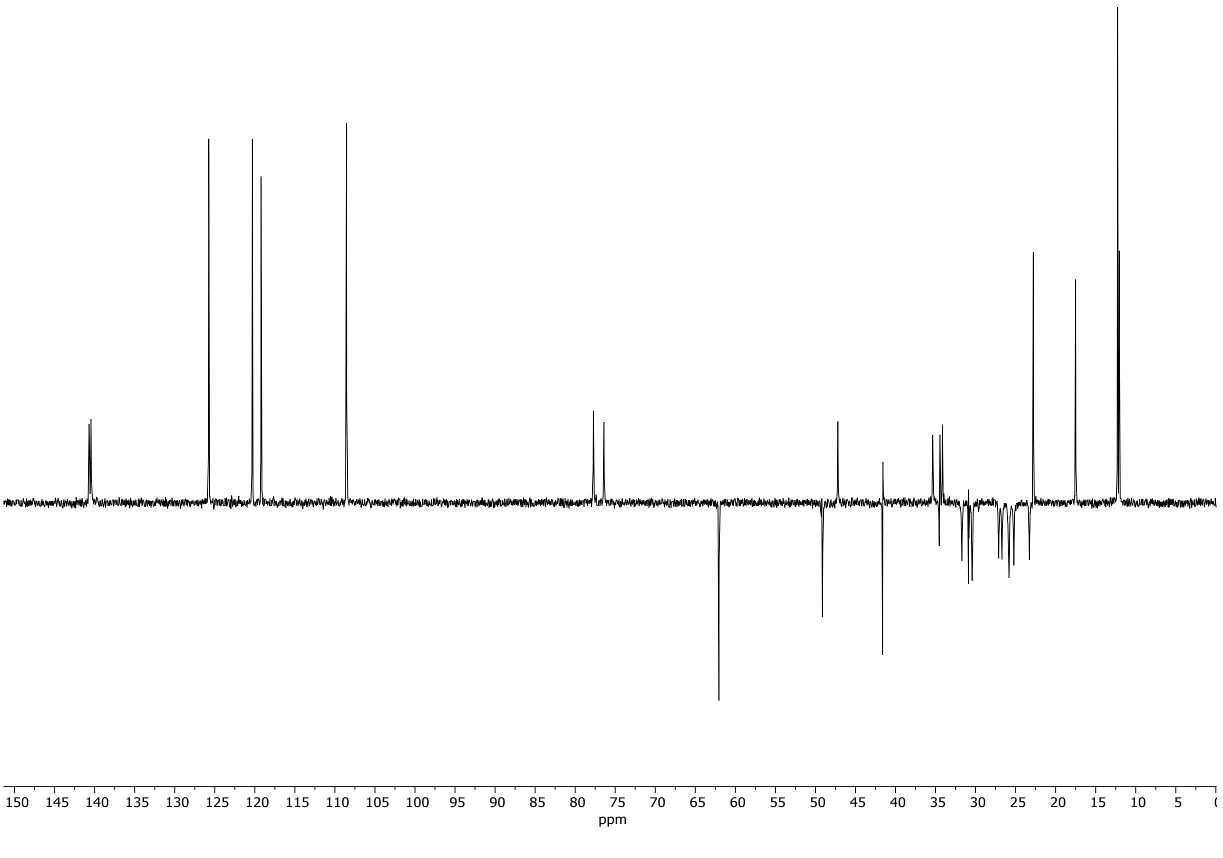

^{1}H-^{13}C HSQC ($CDCl_3$)

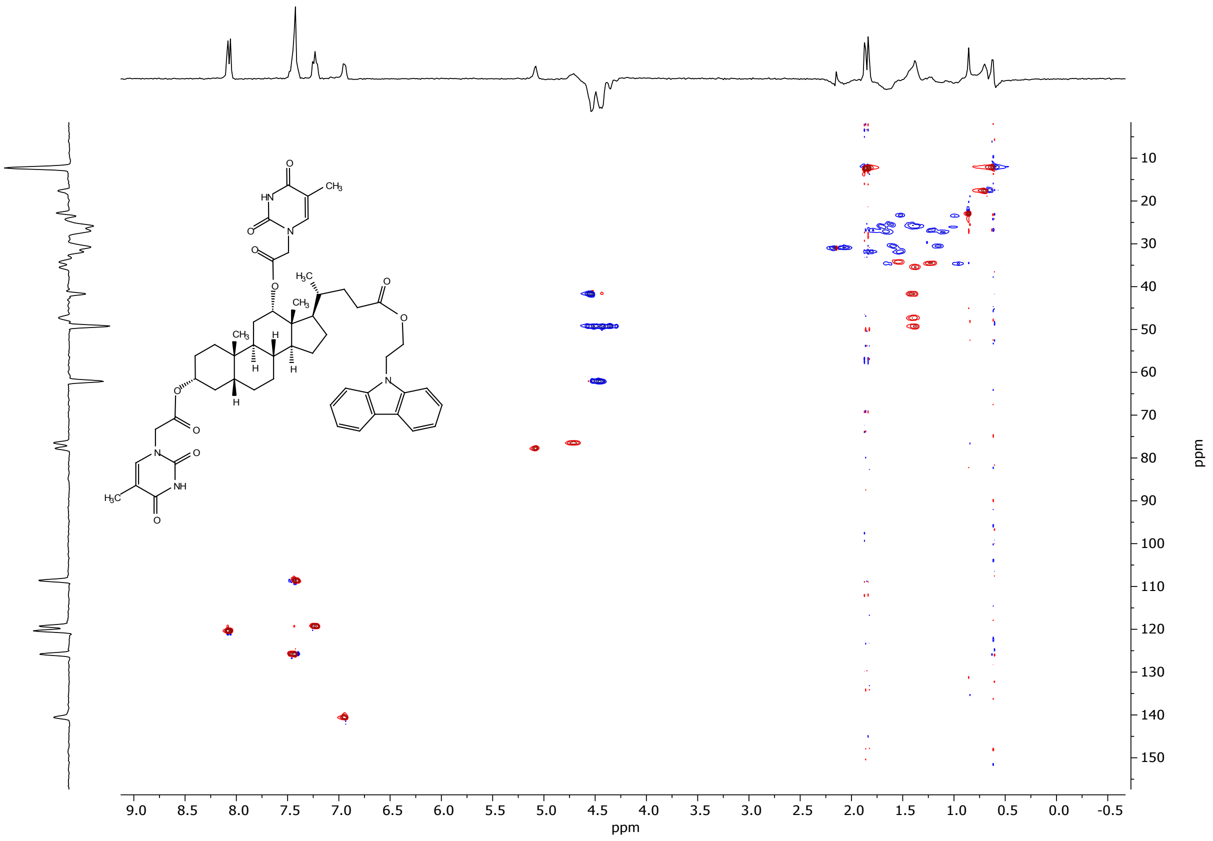

S2.3. ^{1}H and $^{13}C\{1H\}$ NMR of **3**

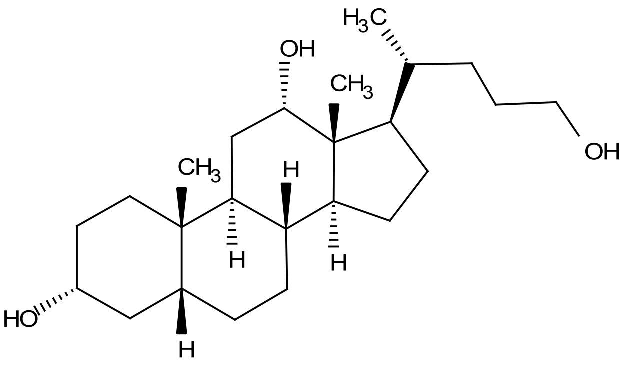

^{1}H NMR (300 MHz, $CDCl_3$)

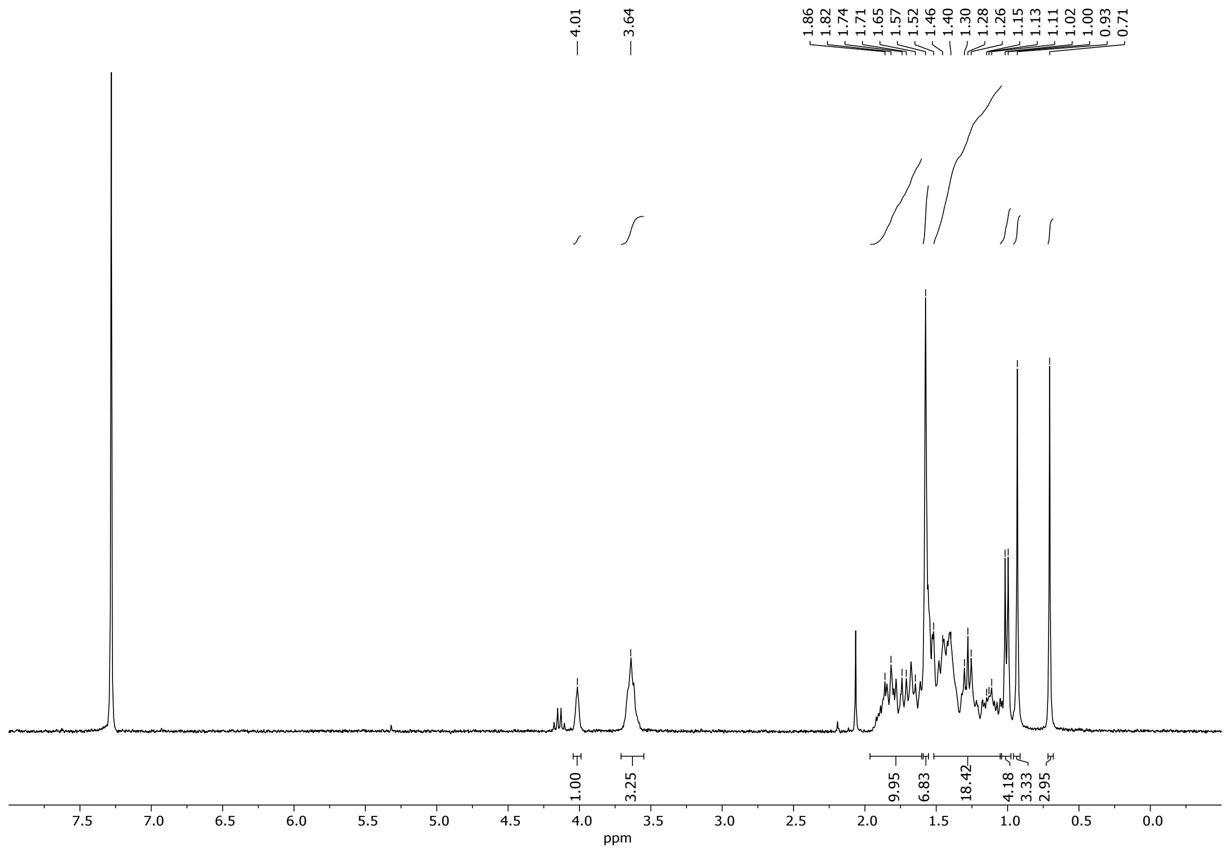

$^{13}C\{1H\}$ NMR (75 MHz, $CDCl_3$)

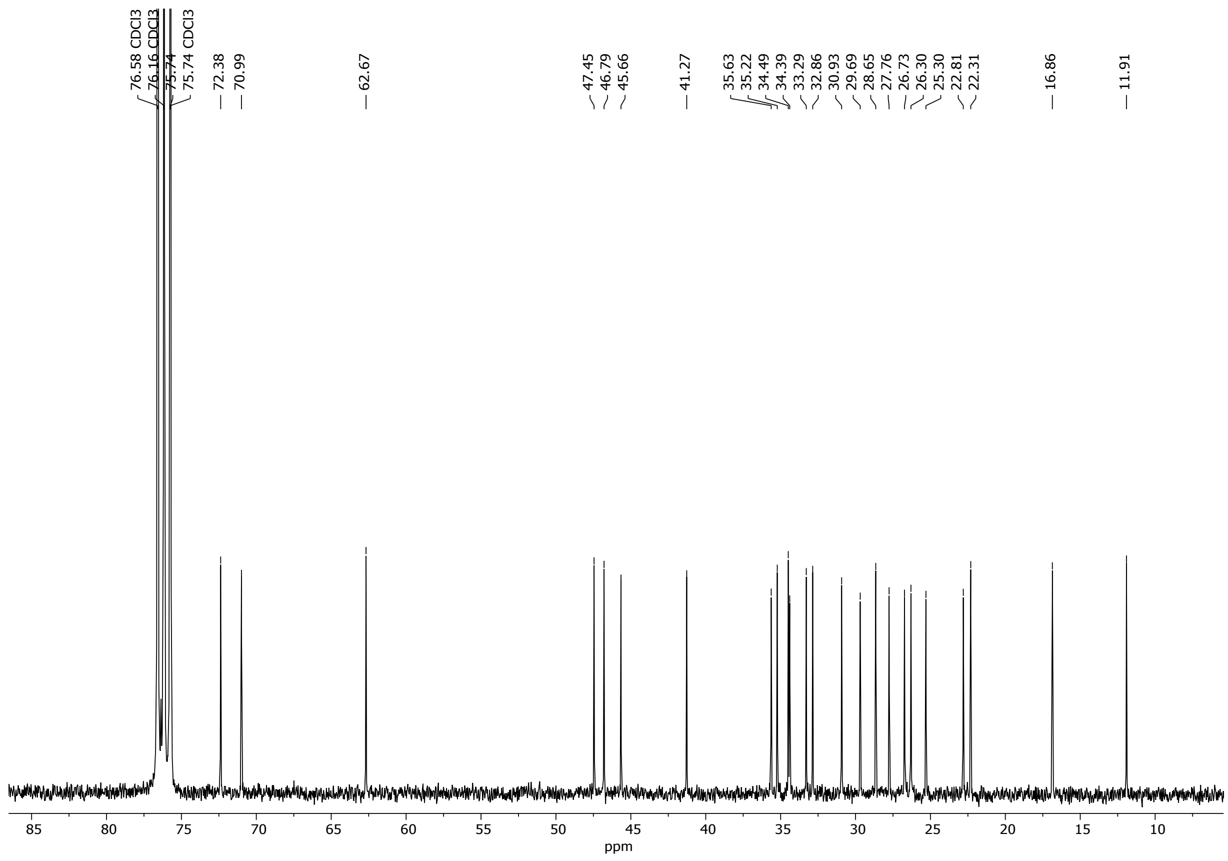

S2.4. ^{1}H, $^{13}C\{1H\}$, $^{13}C\{1H\}$ DEPT-135 and ^{1}H-^{13}C HSQC NMR of **4**

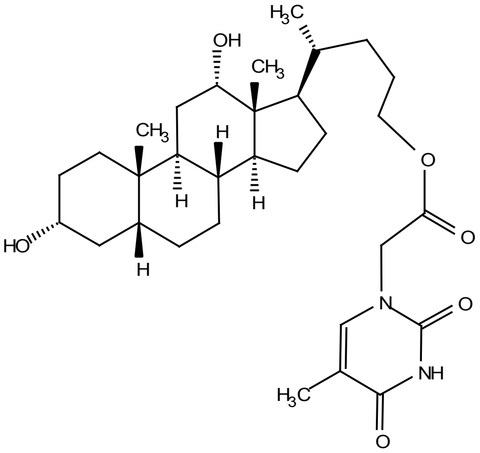

^{1}H NMR (300 MHz, $CDCl_3$)

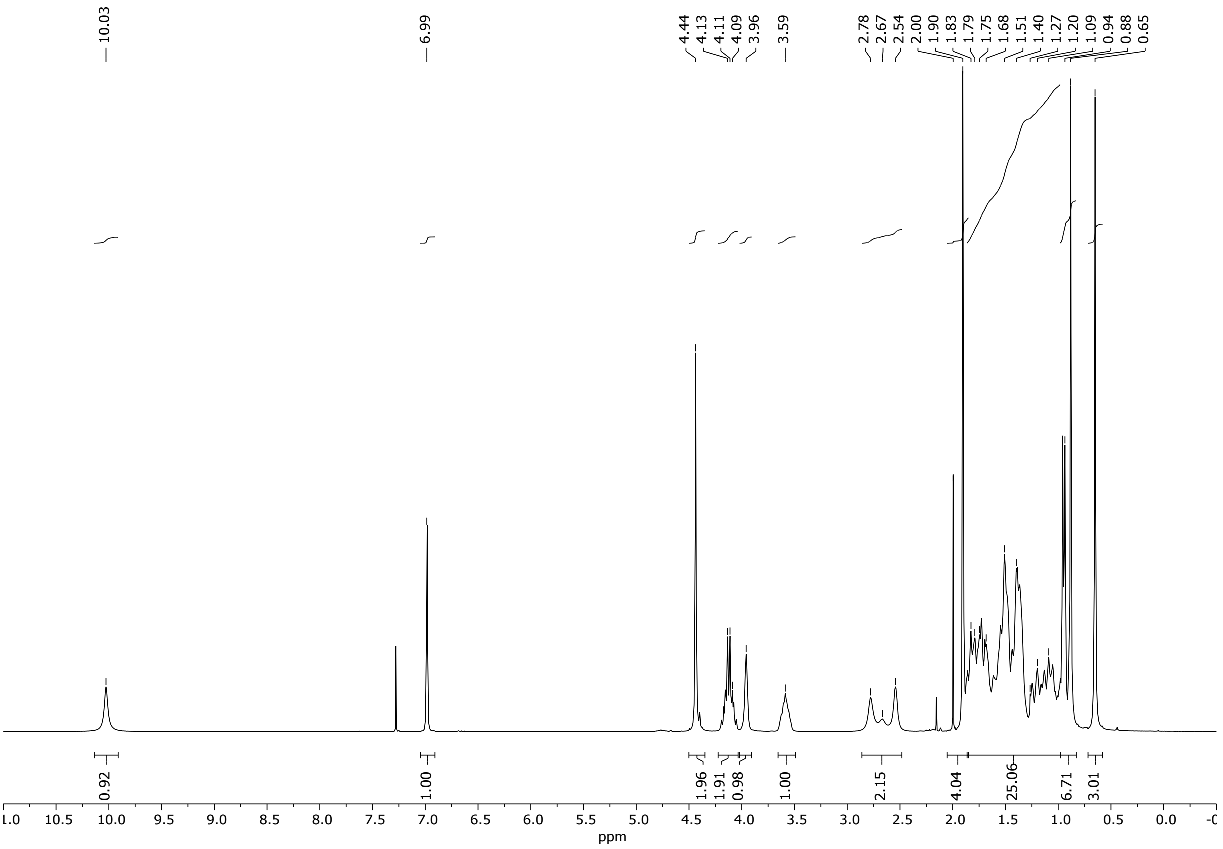

$^{13}C\{1H\}$ NMR (75 MHz, $CDCl_3$)

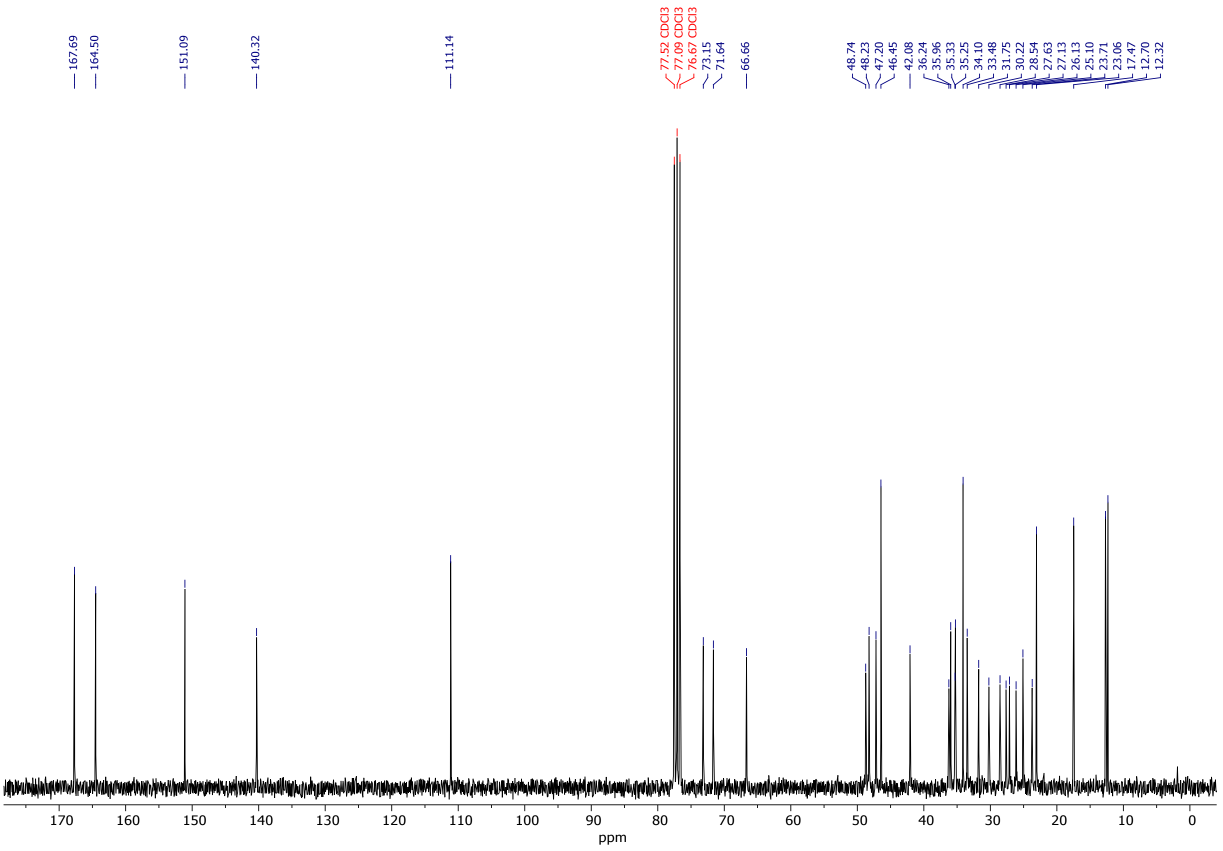

$^{13}C\{1H\}$ DEPT-135 (75 MHz, $CDCl_3$)

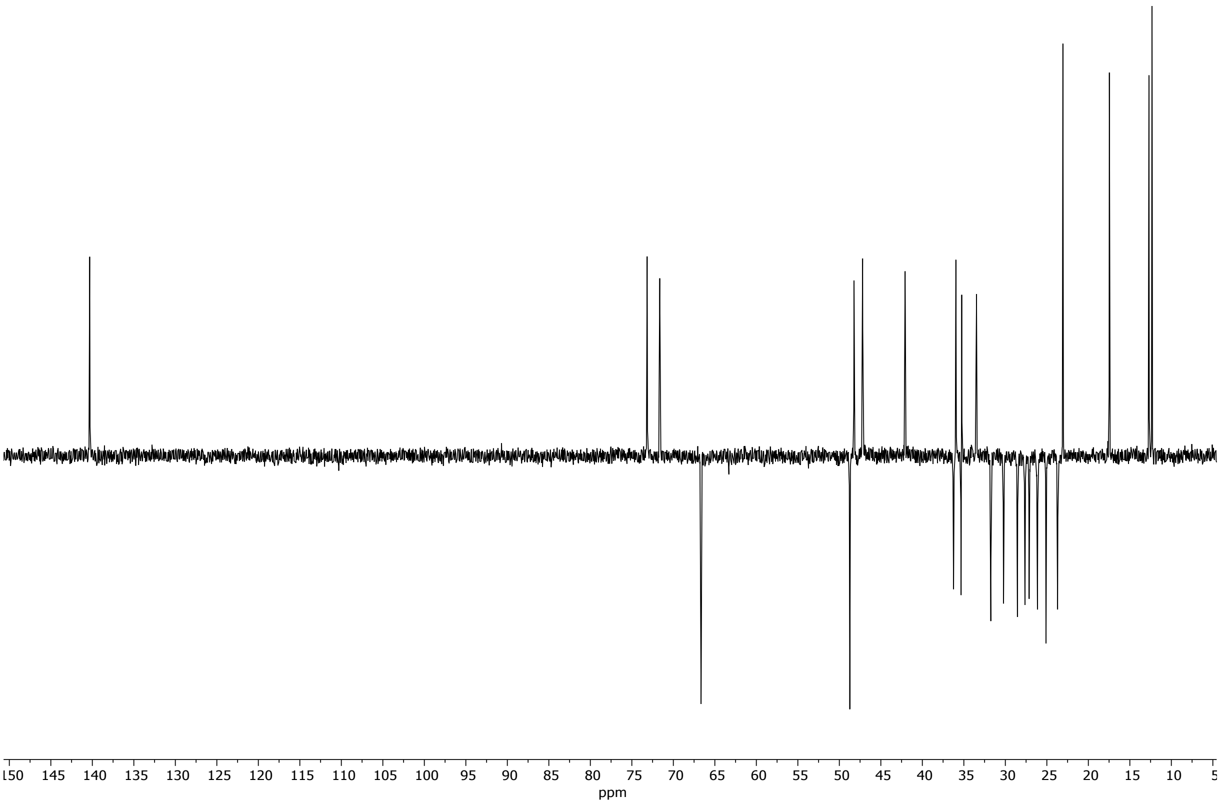

^{1}H-^{13}C HSQC ($CDCl_3$)

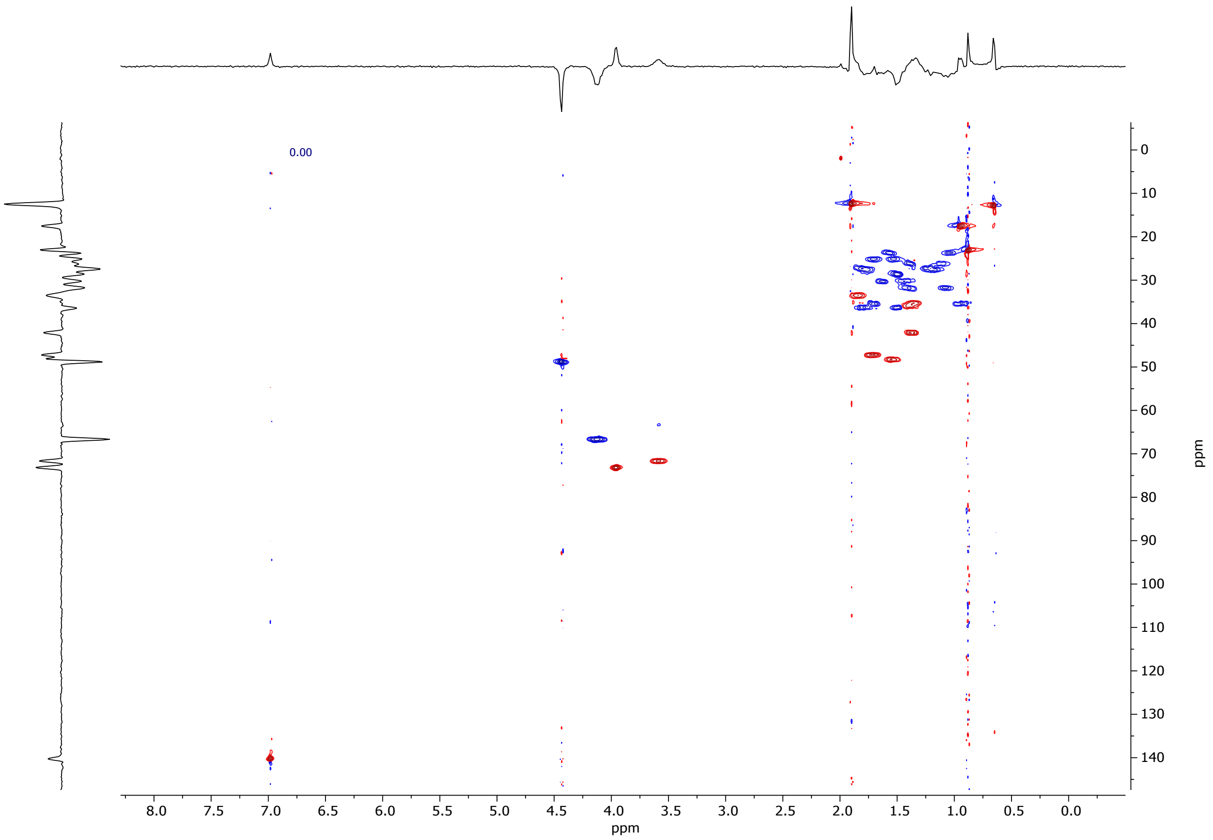

S2.5. ^{1}H, $^{13}C\{1H\}$, $^{13}C\{1H\}$ DEPT-135 and ^{1}H-^{13}C HSQC NMR of **5**

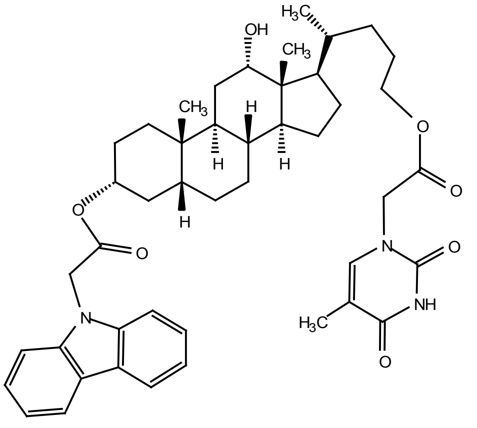

^{1}H NMR (300 MHz, $CDCl_3$)

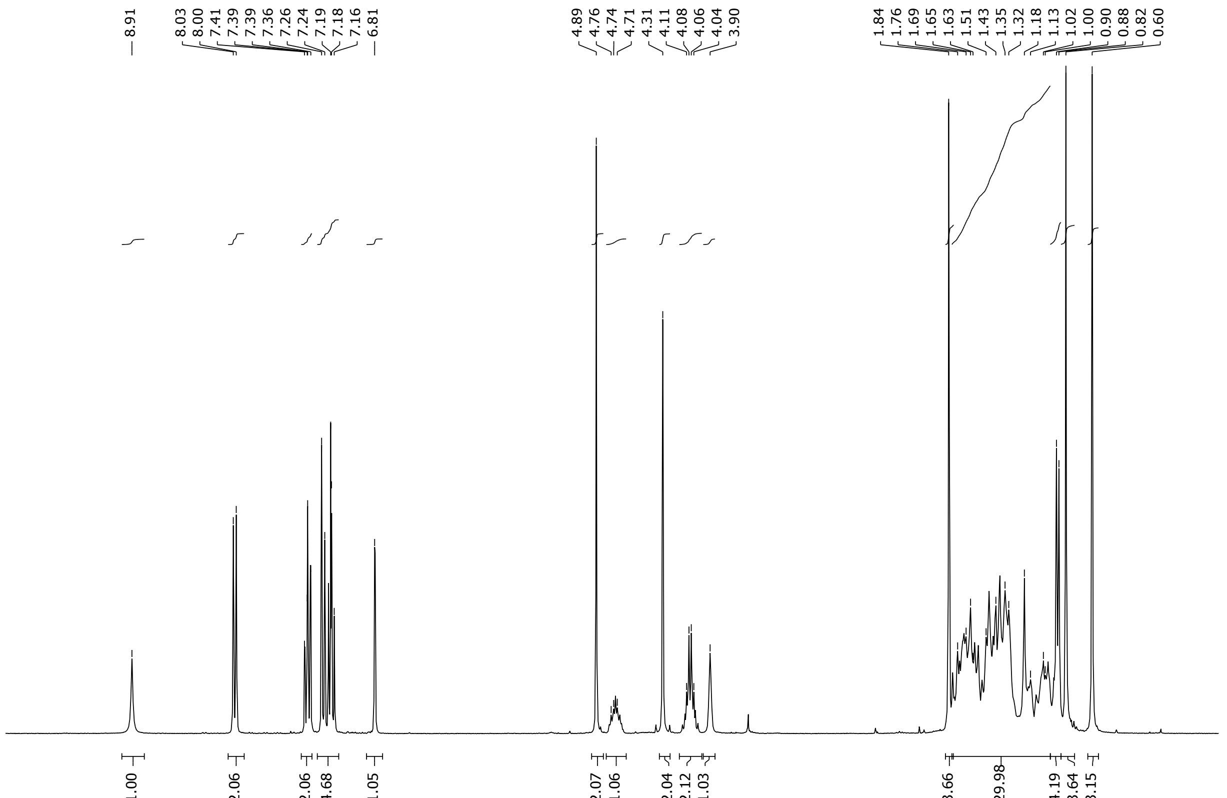

$^{13}C\{1H\}$ NMR (75 MHz, $CDCl_3$)

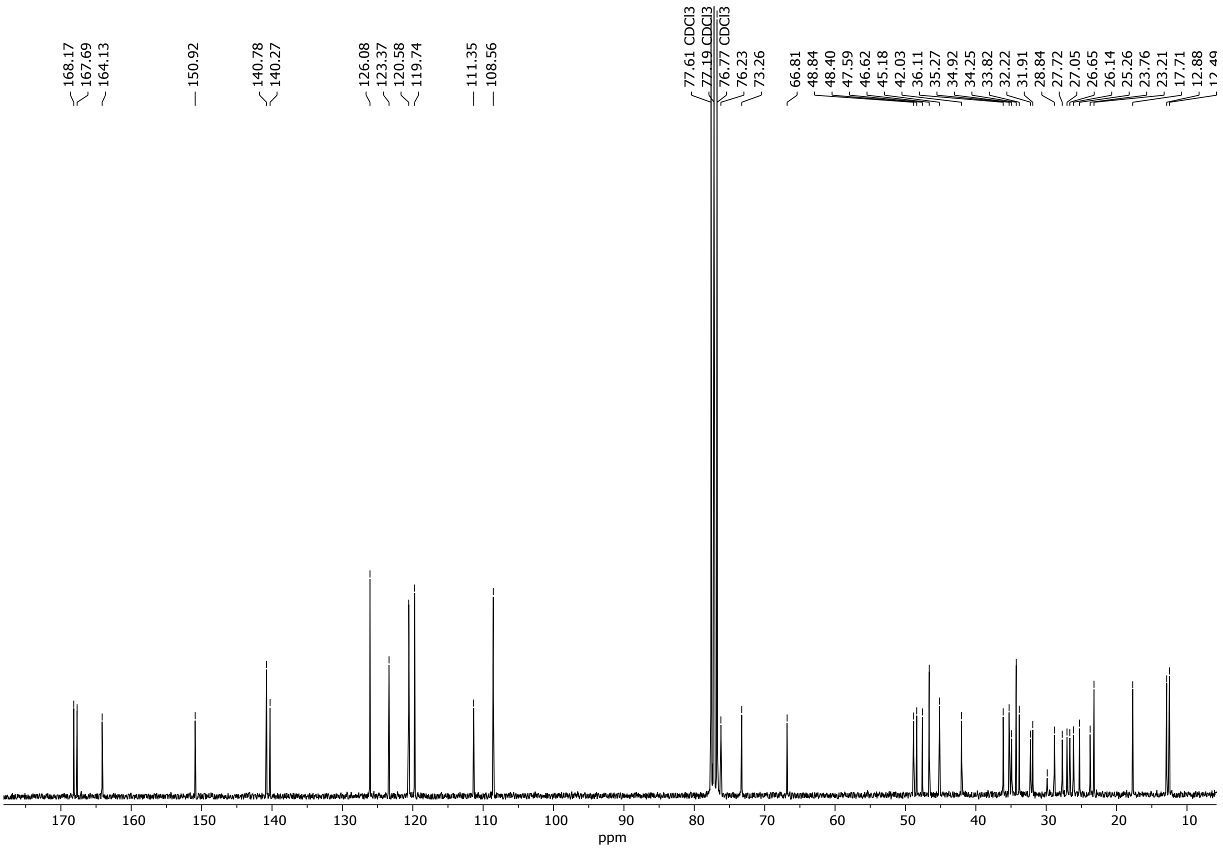

$^{13}C\{1H\}$ DEPT-135 (75 MHz, $CDCl_3$)

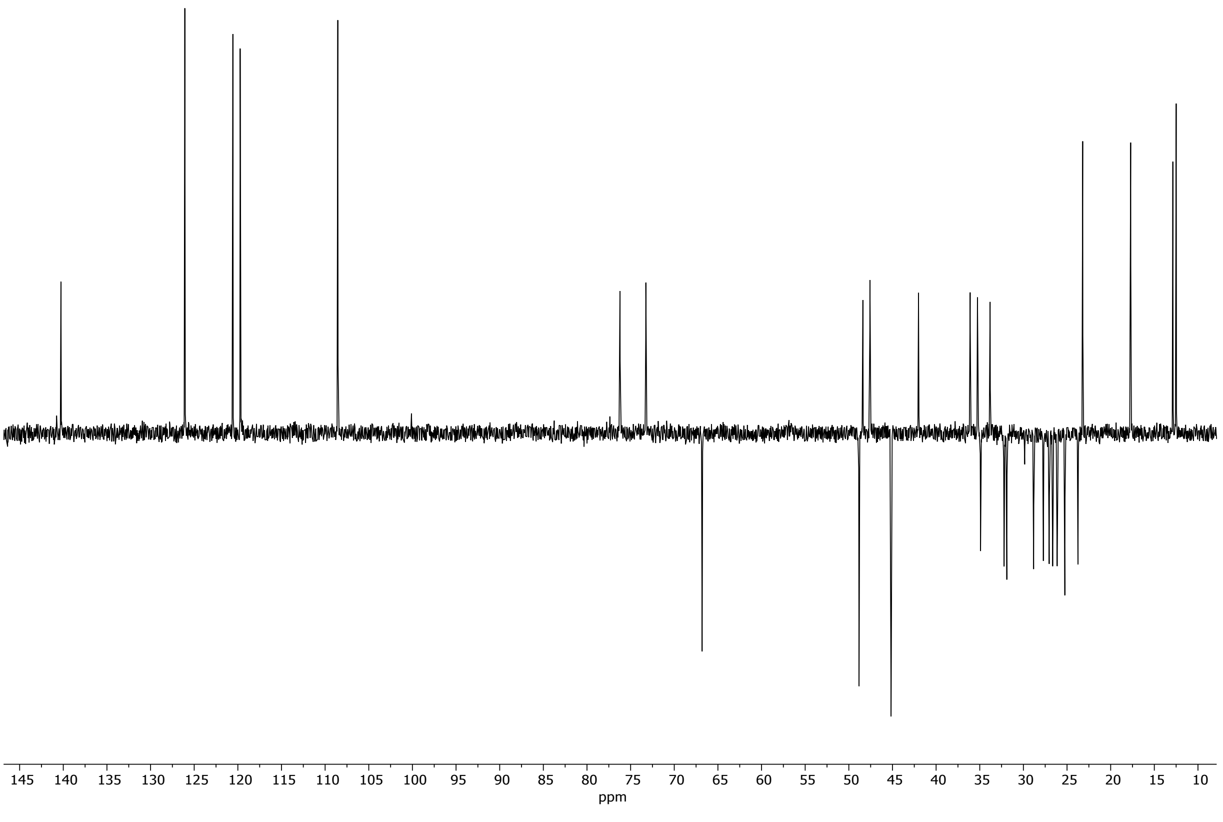

^{1}H-^{13}C HSQC ($CDCl_3$)

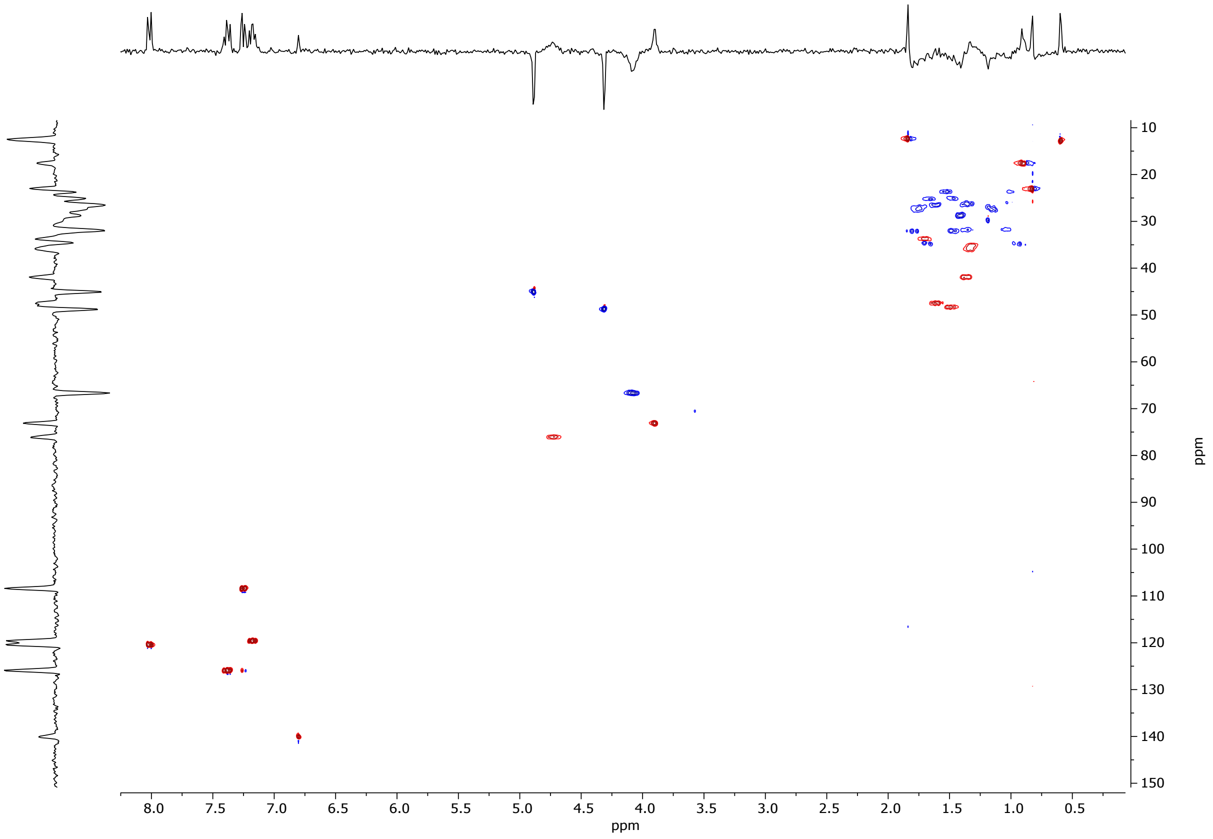

S2.6. ^{1}H, $^{13}C\{1H\}$, $^{13}C\{1H\}$ DEPT-135 and ^{1}H-^{13}C HSQC NMR of **6**

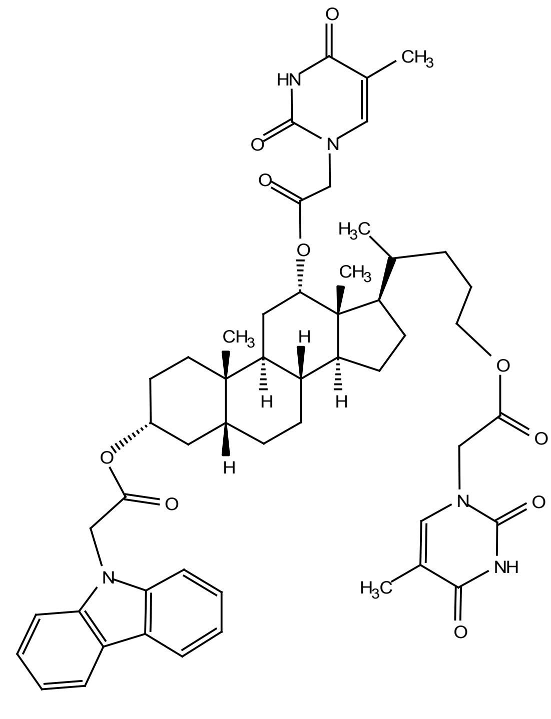

^{1}H NMR (300 MHz, $CDCl_3$)

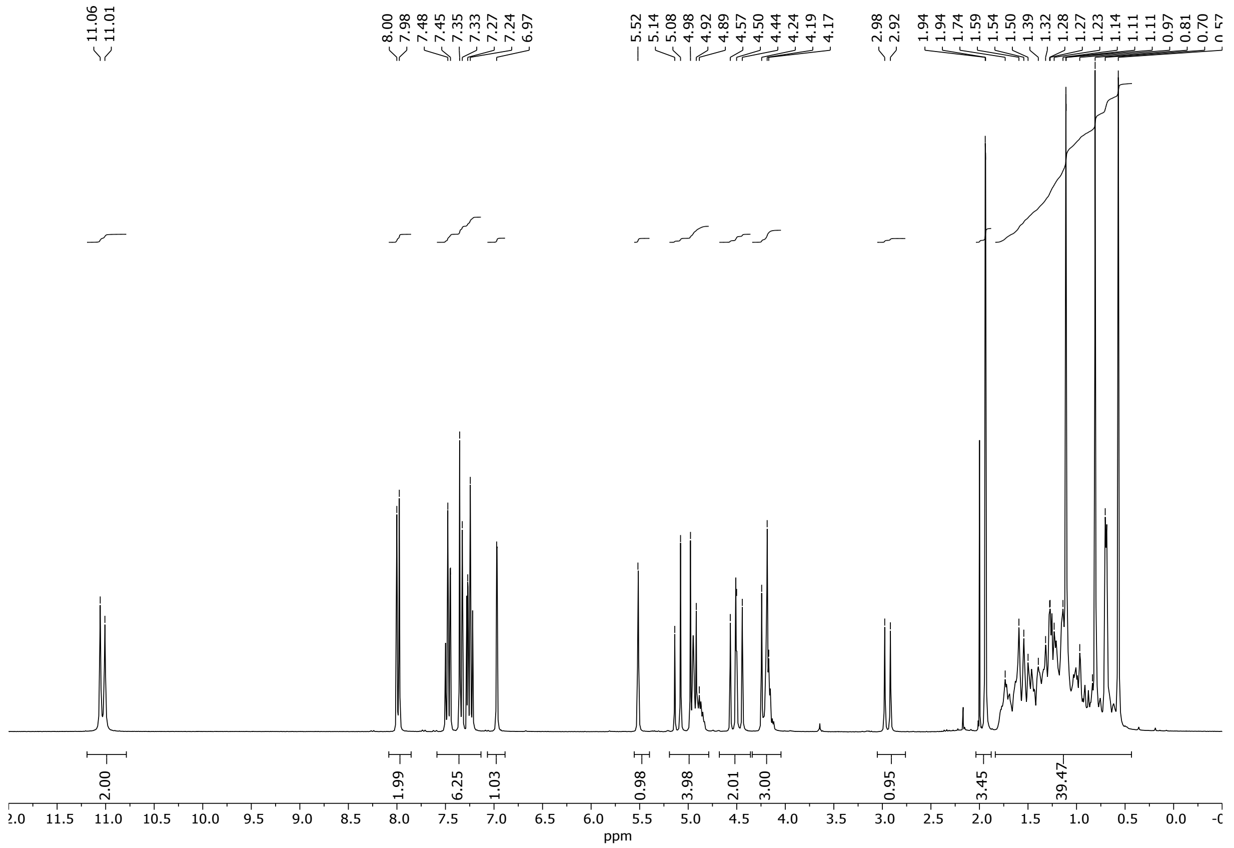

$^{13}C\{1H\}$ NMR (75 MHz, $CDCl_3$)

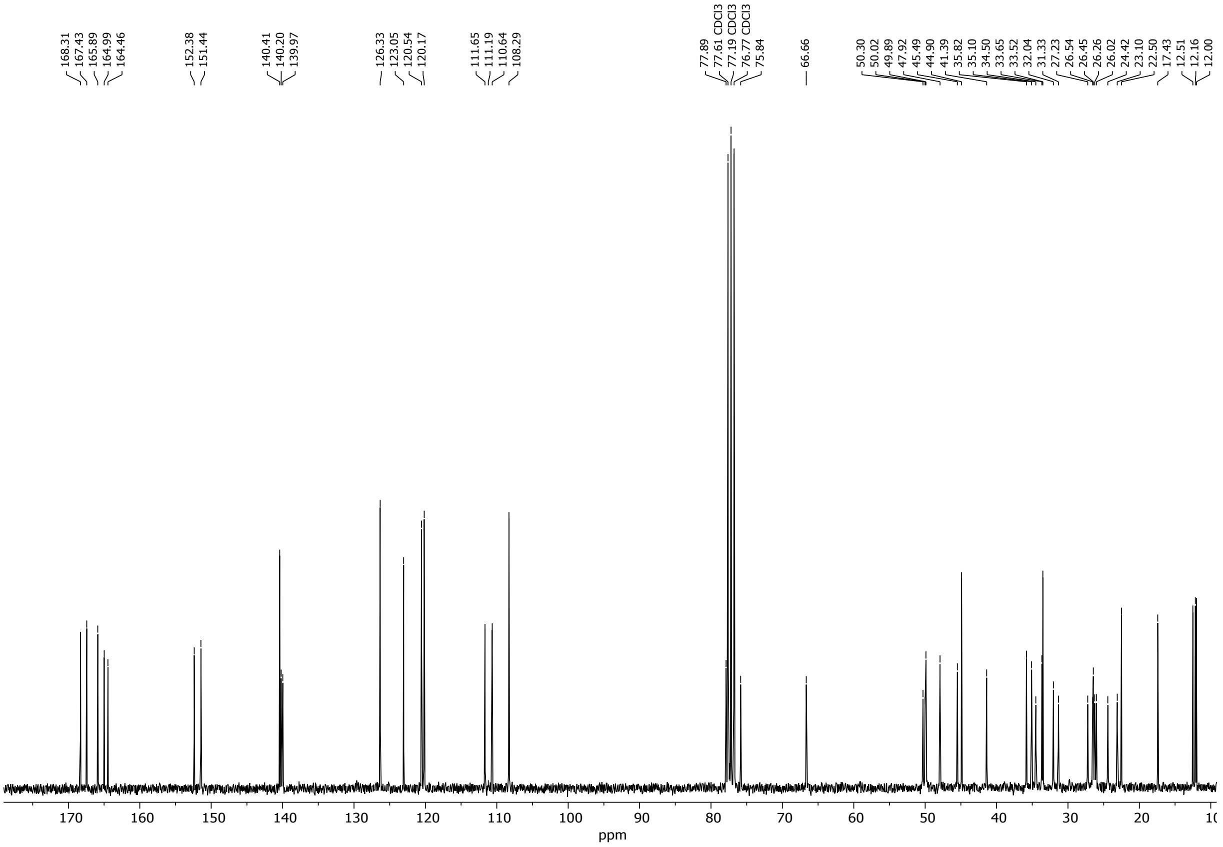

$^{13}C\{1H\}$ DEPT-135 (75 MHz, $CDCl_3$)

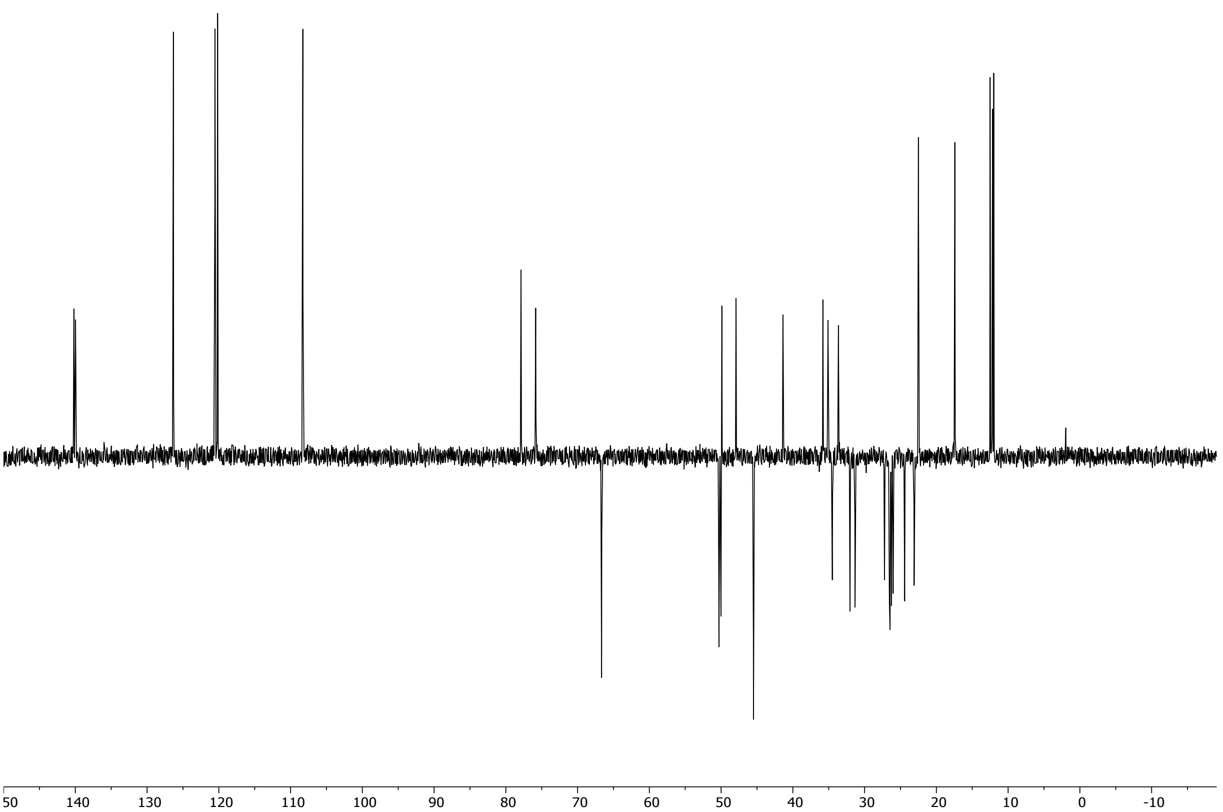

^{1}H-^{13}C HSQC ($CDCl_3$)

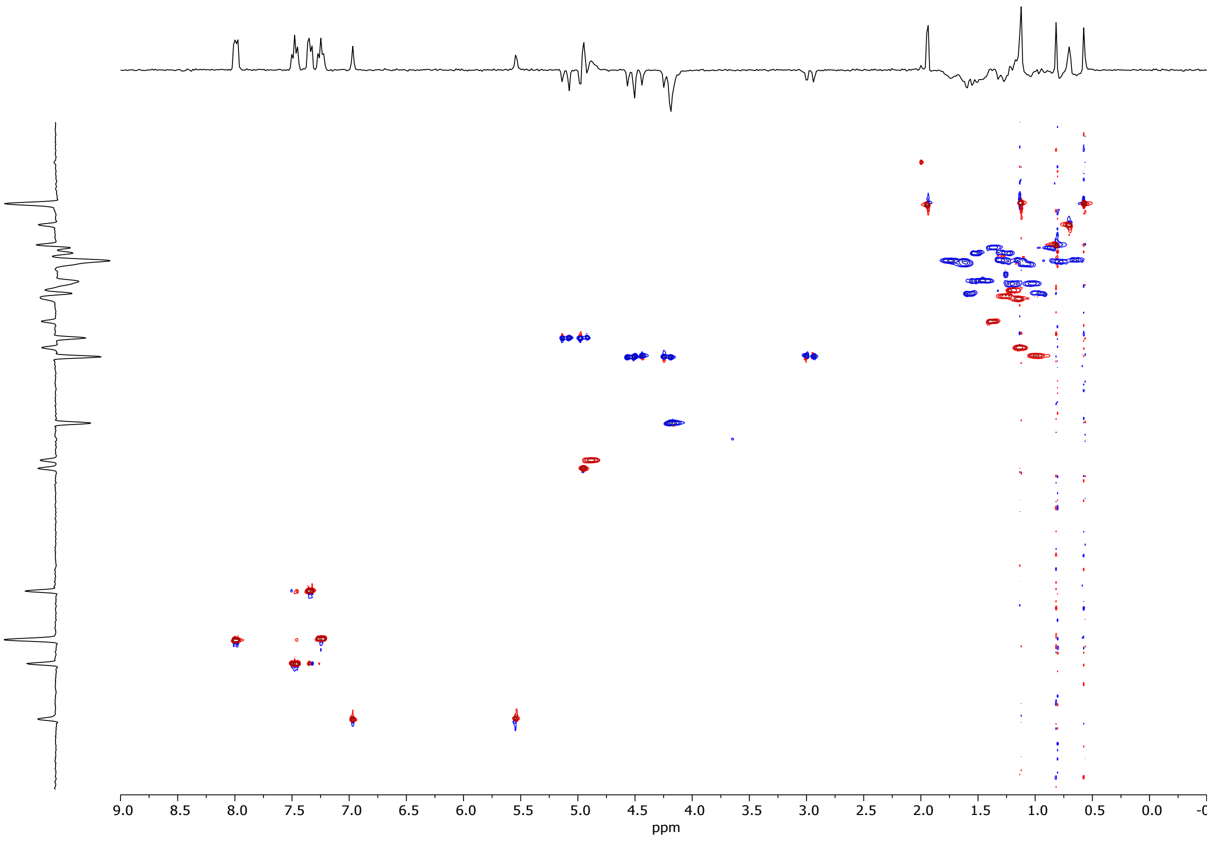

S2.7. ^{1}H, ^{13}C{1H}, ^{13}C{1H} DEPT-135, ^{1}H-^{13}C HSQC, ^{1}H-^{1}H COSY and ^{1}H-^{1}H NOESY NMR of **7**

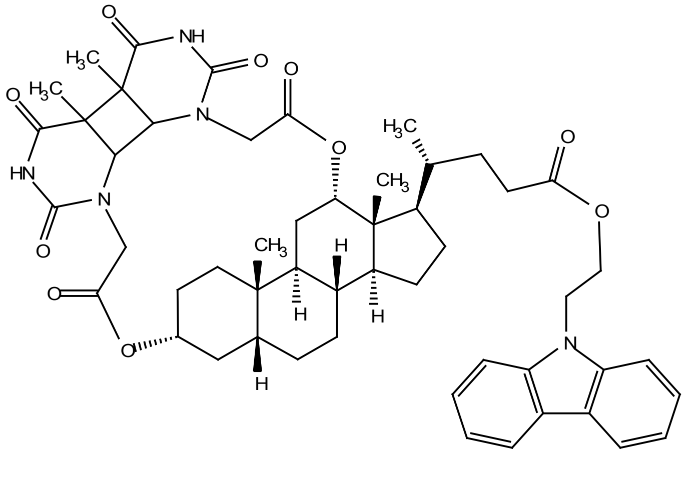

^{1}H NMR (400 MHz, C_5D_5N)

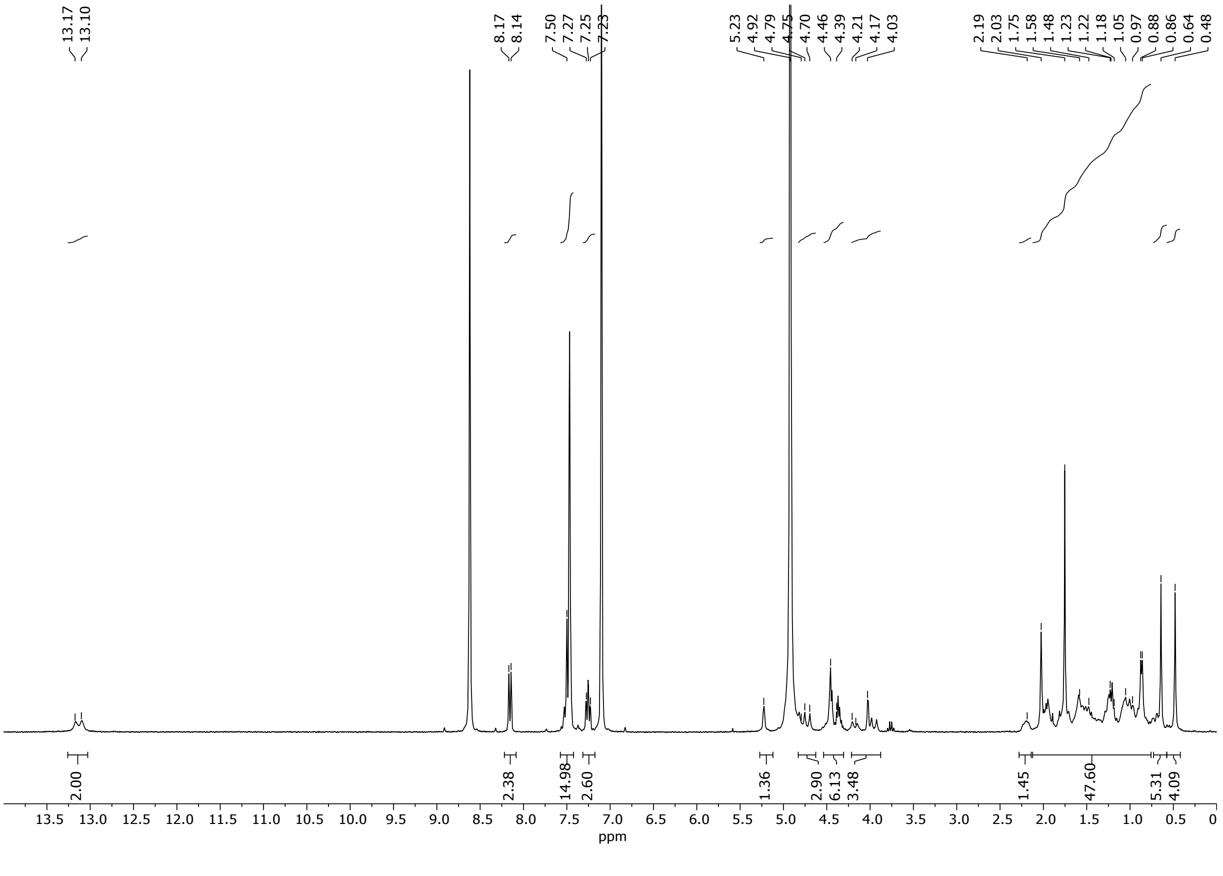

$^{13}C\{1H\}$ NMR (100 MHz, C_5D_5N)

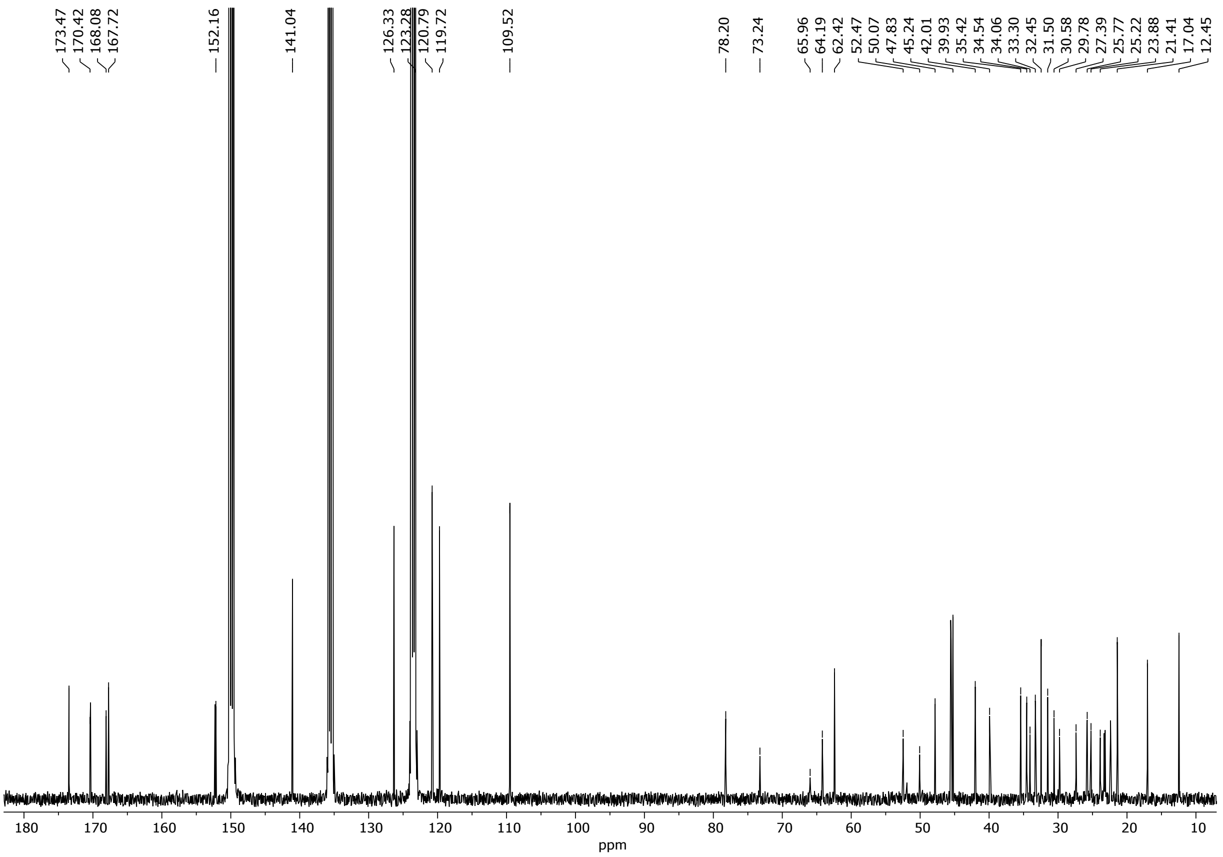

$^{13}C\{1H\}$ DEPT-135 (100 MHz, C_5D_5N)

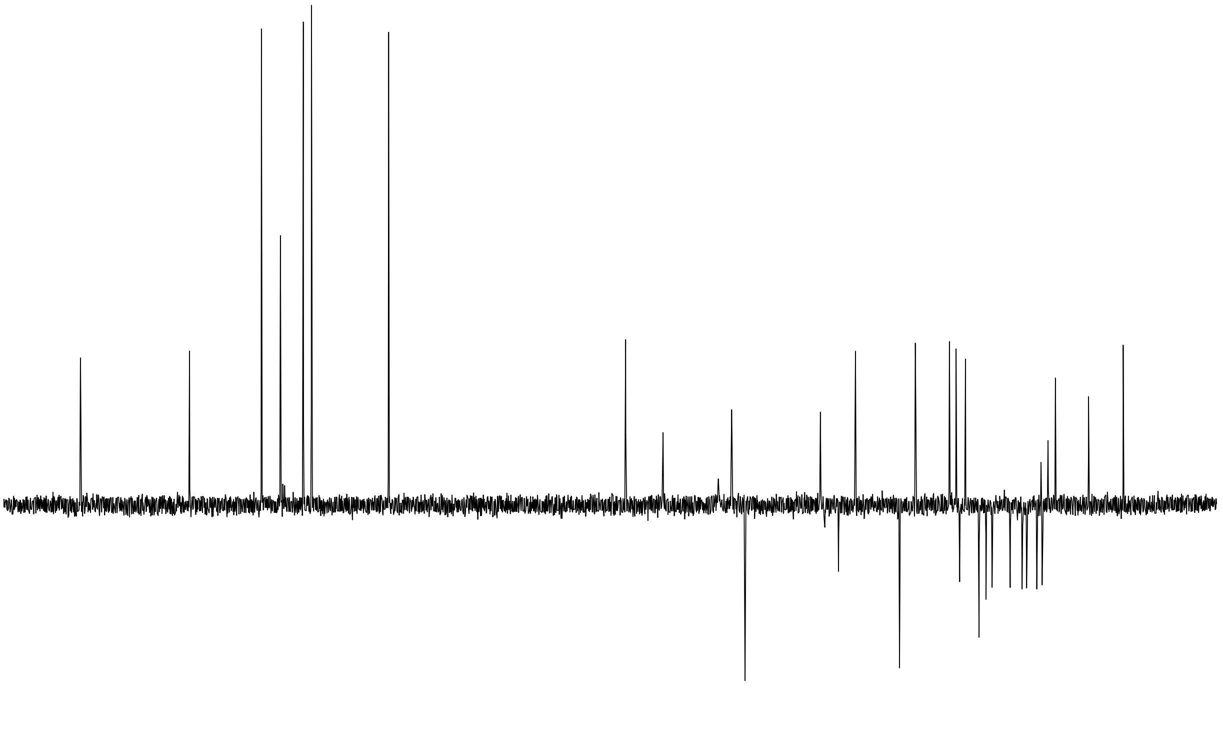

^{1}H-^{13}C HSQC (C_5D_5N)

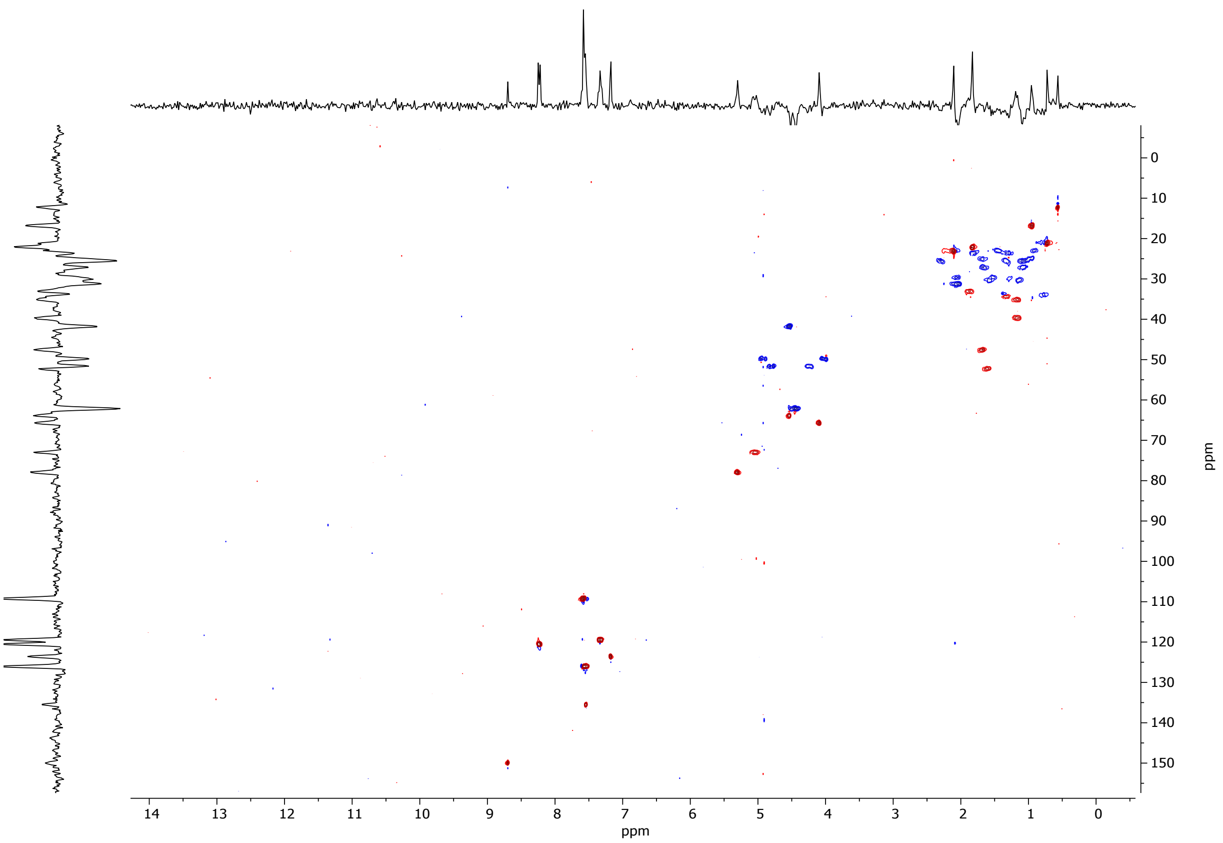

^{1}H-^{1}H COSY (C_5D_5N)

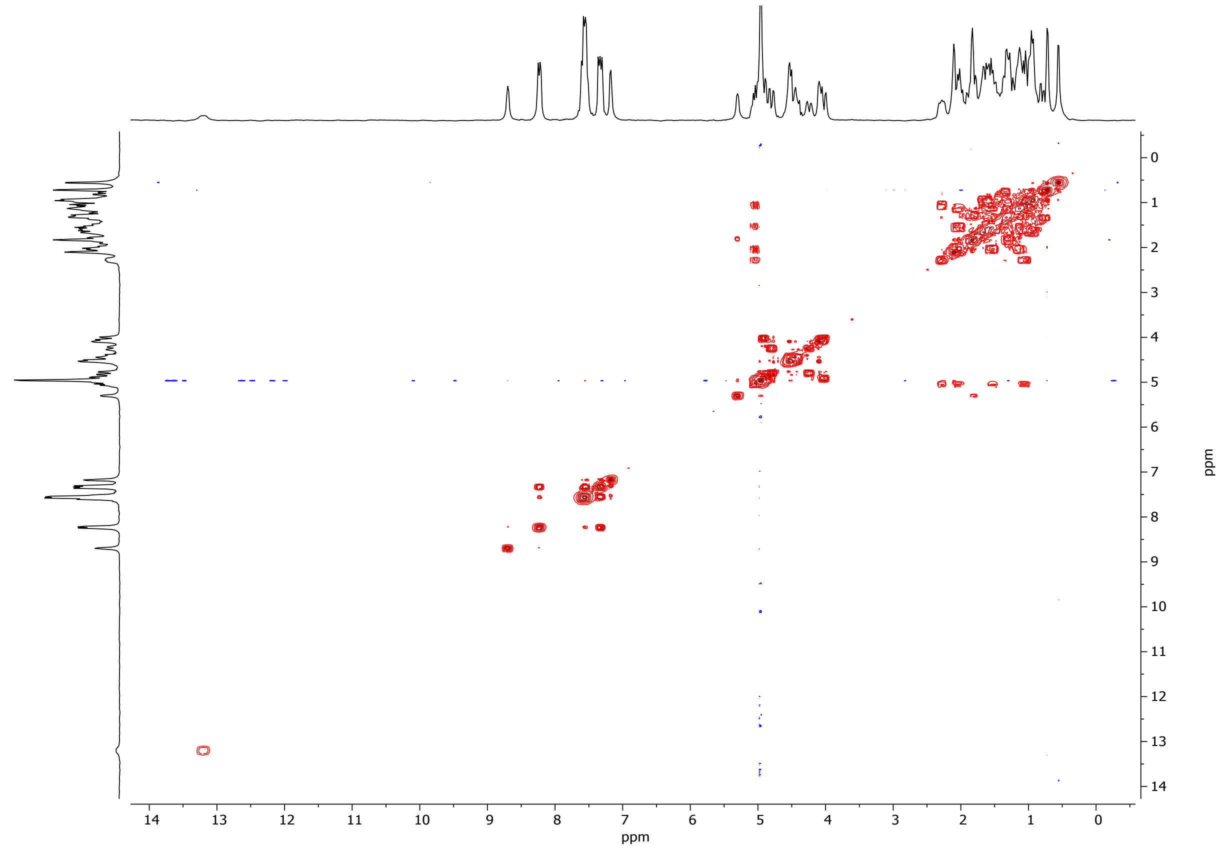

^{1}H-^{1}H NOESY (C_5D_5N)

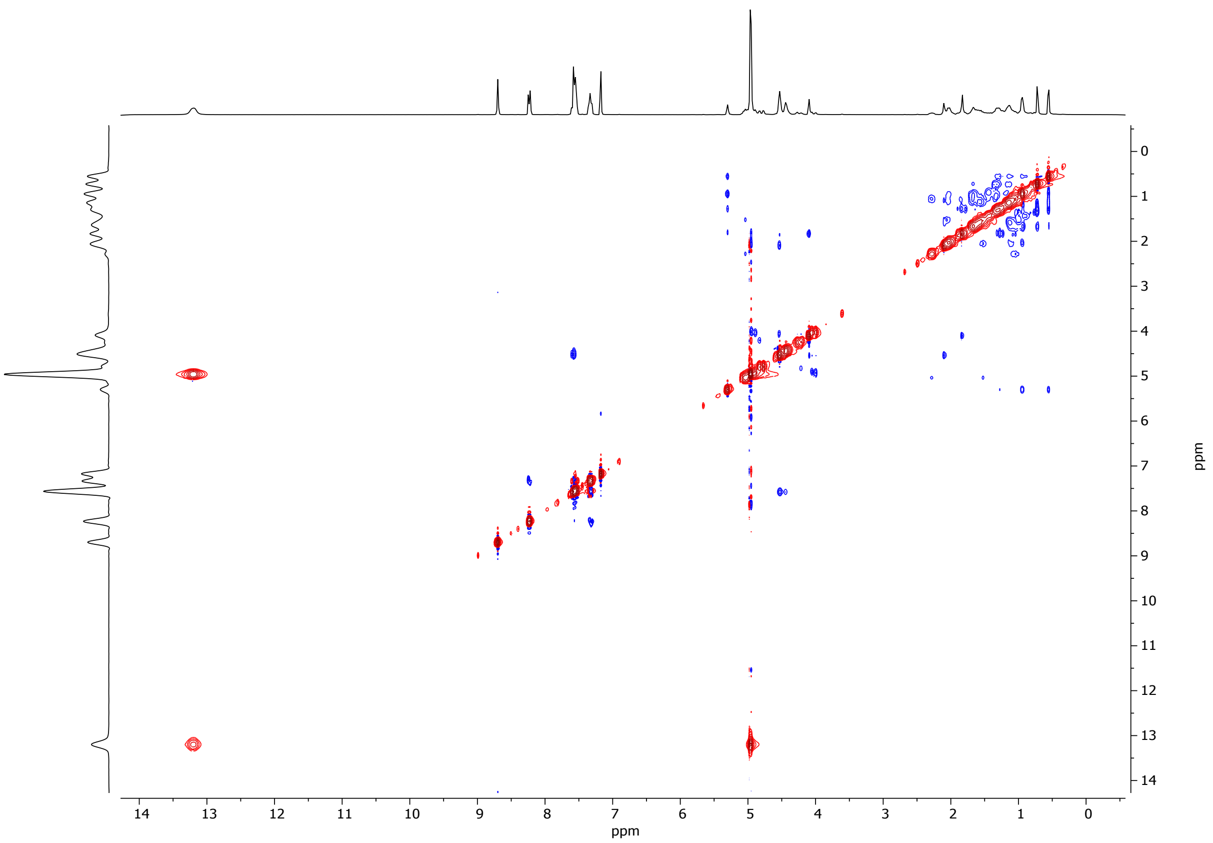

S2.8. ^{1}H, ^{13}C{1H}, ^{13}C{1H} DEPT-135, ^{1}H-^{13}C HSQC, ^{1}H-^{1}H COSY, ^{1}H-^{1}H NOESY NMR and molecular structure of **8**

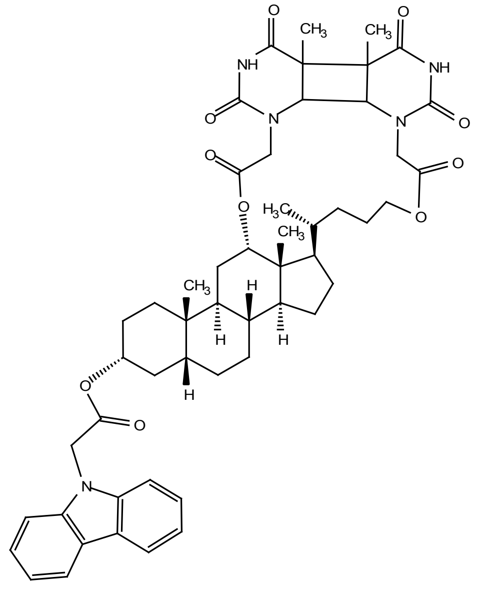

^{1}H NMR (300 MHz, CDCl$_3$)

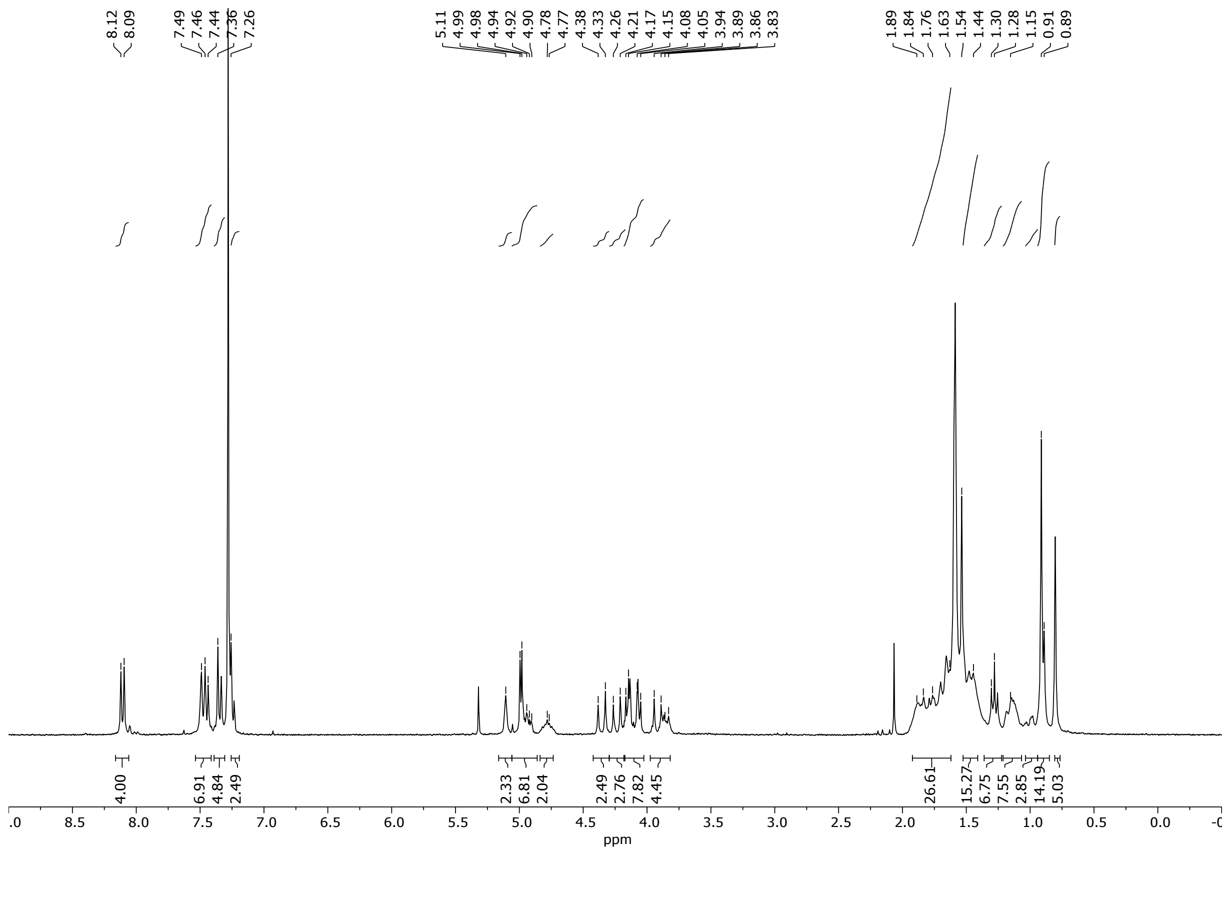

$^{13}C\{1H\}$ NMR (75 MHz, $CDCl_3$)

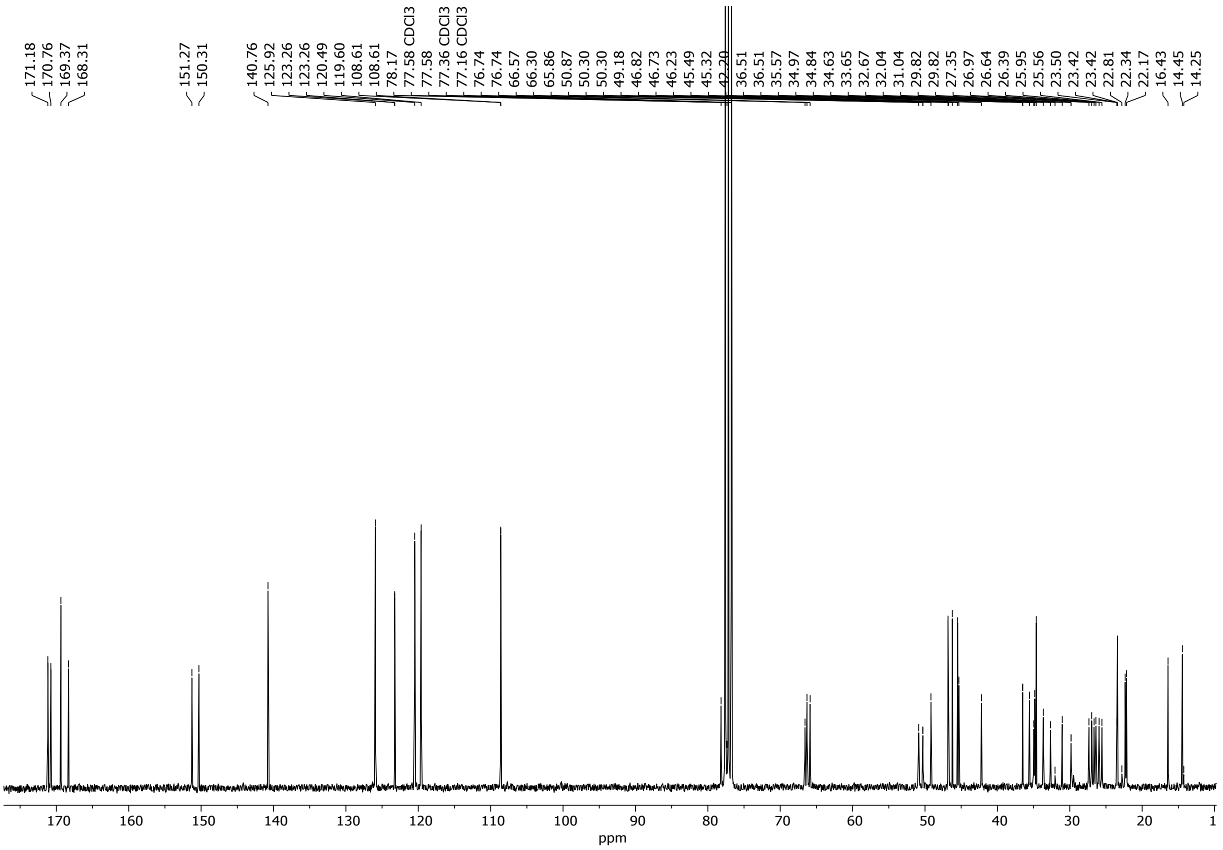

$^{13}C\{1H\}$ DEPT-135 (75 MHz, $CDCl_3$)

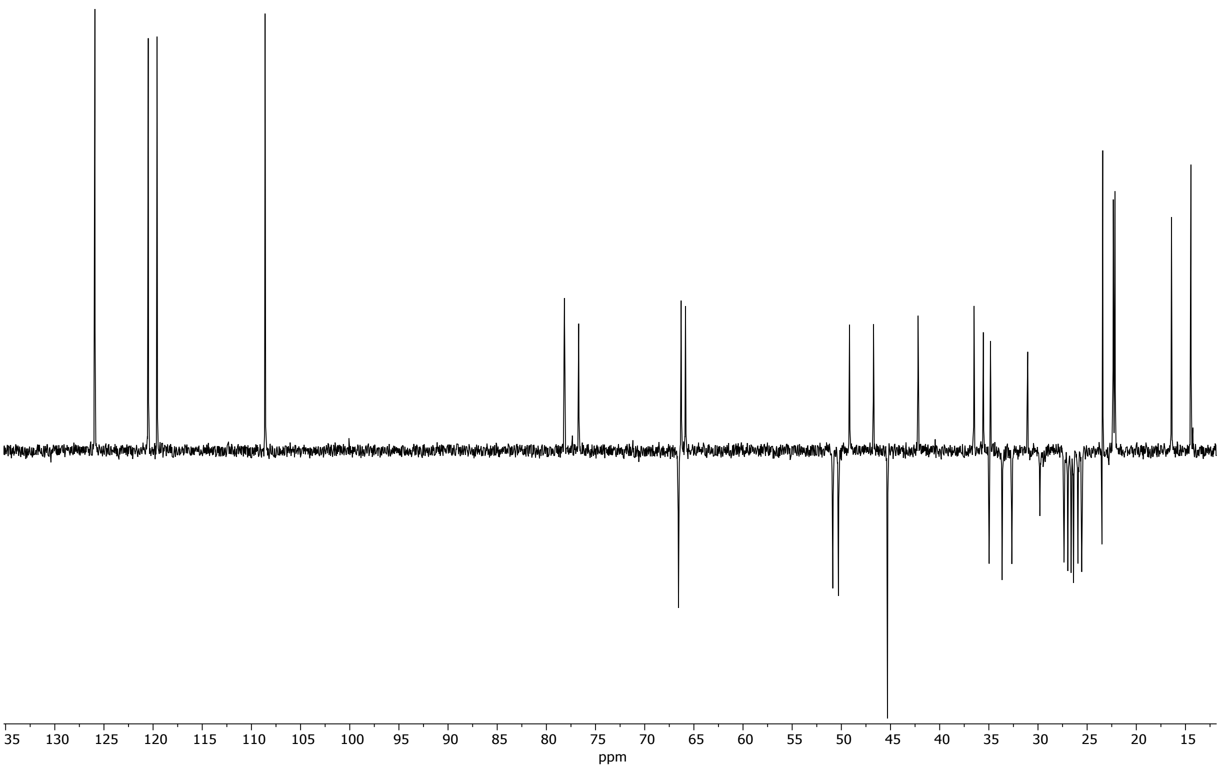

^{1}H-^{13}C HSQC ($CDCl_3$)

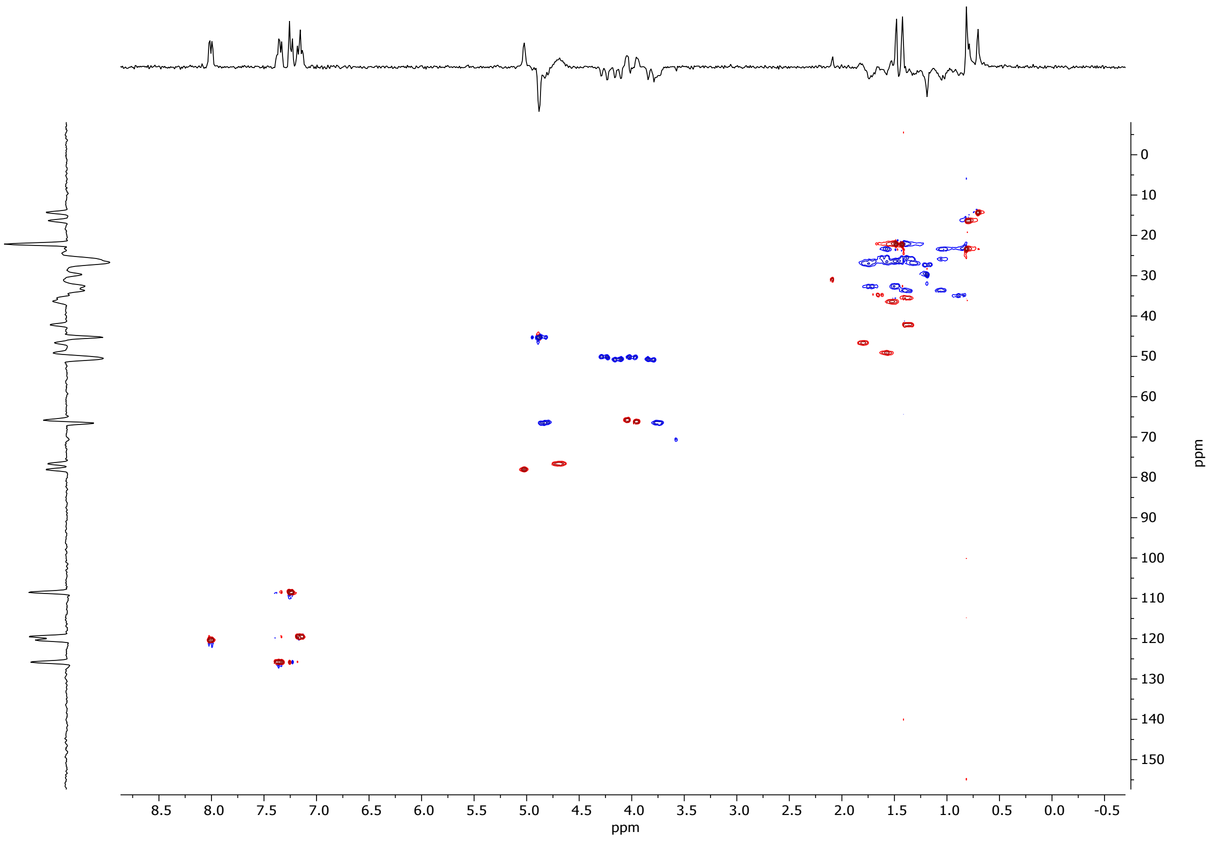

^{1}H-^{1}H COSY ($CDCl_3$)

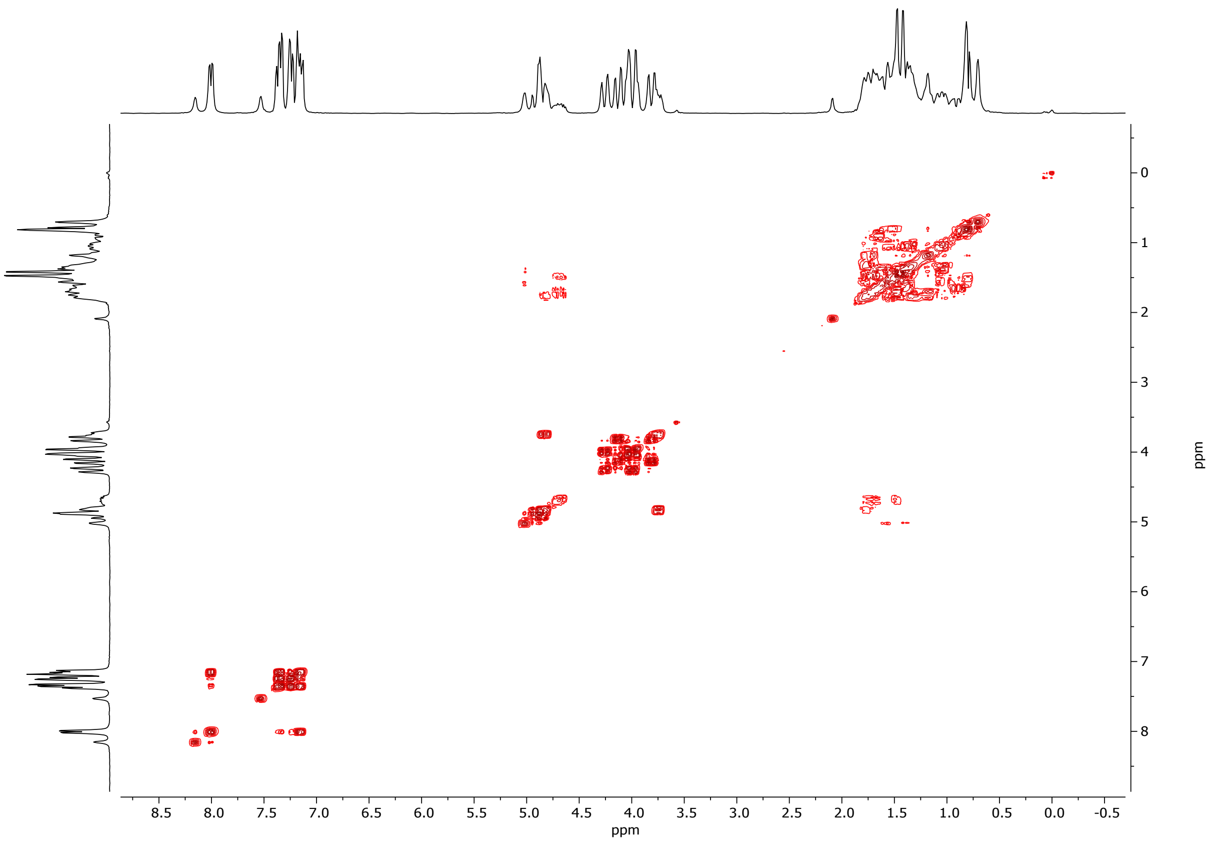

^1H-^1H NOESY ($CDCl_3$)

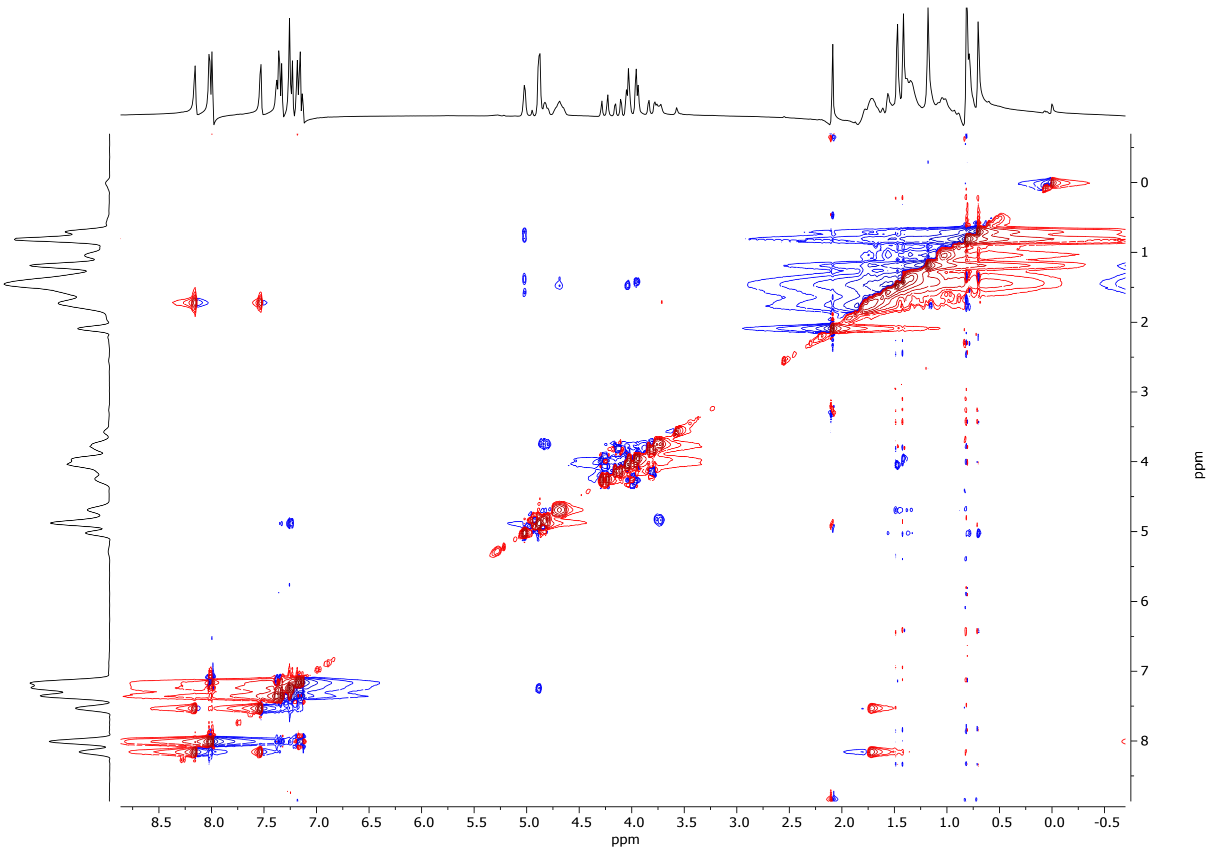

S3. X Rays

S3.1. Experimental

The cristal was prepared dissolving 4.2 mg of compound **8** in a mixture 4:1 acetonitrile: distilled water. The sample was kept in a closed vial in the dark at room temperature for 40 days until the cristal grew up.

Single cristal diffraction data were collected at 100(2) K with monochromated Cu Kα radiation (λ = 1.54178 Å) using narrow frame (0.3°) omega and fi scans on a Bruker D8 VENTURE diffractometer. Measured intensities were integrated and corrected for absorption effects with SAINT[1] and SADABS[2] programs, included in APEX4 package.[3] The structure was solved by direct methods with SHELXS progam[4] and refined by full-matrix least-squares refinement on F2 with SHELXL program,[5] included in WingX package.[6] Hydrogen atoms were included at the calculated positions, riding on their carrier atoms. The absolute configuration was determined on the basis of previously known internal reference, and this assignment was confirmed using the Flack parameter.[7] At the last steps of the refinement, there were large solvent accesible voids in the structure. Some solvent was found to be highly disordered, and it cannot be properly modelled. Therefore, SQUEEZE corrections have been applied.[8]

S3.2. References

1. SAINT+, version 6.01: Area-Detector Integration Software, Bruker AXS, Madison **2001**.
2. (a) SADABS (Version 2016/02), Bruker AXS, Madison **2016**. (b) L. Krause, R. Herbst-Irmer, G.M. Sheldrick, D. Stalke, *J. Appl. Crystallogr.* **2015**, *48*, 3–10.
3. Bruker APEX4. APEX4 V2021.4-1, Bruker-AXS, Madison, WI, USA, 2021.
4. G. M. Sheldrick, Acta Crystallogr., Sect. A: Found. Crystallogr., **1990**, *46*, 467–473; (b) G. M. Sheldrick, Acta Crystallogr., Sect. A: Found. Crystallogr., **2008**, *64*, 112–122.
5. G. M. Sheldrick, Acta Crystallogr., Sect. C: Struct. Chem., **2015**, *71*, 3–8.
6. L. J. Farrugia, J. Appl. Crystallogr., **2012**, *45*, 849–854.
7. H.D Flack, Acta Crystallogr., Sect. A: Found. Crystallogr., **1983**, *39*, 876–881.
8. P.V. D. Sluis, A.L. Spek, Acta Crystallogr., Sect. A: Found. Crystallogr., **1990**, *46*, 194-201.

S3.3. Characterization

Crystal data compound **8** (Figure S3.1). $C_{52}H_{63}3N_5O_{10}\cdot 3(C_2H_3N)\cdot C_6H_{14}\cdot H_2O$, M = 672.41; colourless block 0.115 × 0.180 × 0.270 mm3; monoclinic $P2_1^-$, a = 14.2544(6), b = 27.7891(12), c = 15.4421(7) Å, β = 90.963(2)°, V = 6116.0(5) Å^3; Z = 4; D_c = 1.233 g cm^{-3}; μ = 0.689 mm^{-1}; T_{min}/T_{max}: 0.6092/0.7453; reflections measured/unique 217745 / 25812 (Rint = 0.0477), number of data/restraint/parameters 25812 / 4 / 1385, $R_1(F^2)$ = 0.0606 (25366 reflections, I > 2σ(I)) and wR(F2) = 0.1714 (all data), final GoF = 1.046, largest difference peak: 0.325 e Å^{-3}. Flack parameter: 0.11(4). CCDC 2159900 contains the supplementary crystallographic data for this structure.

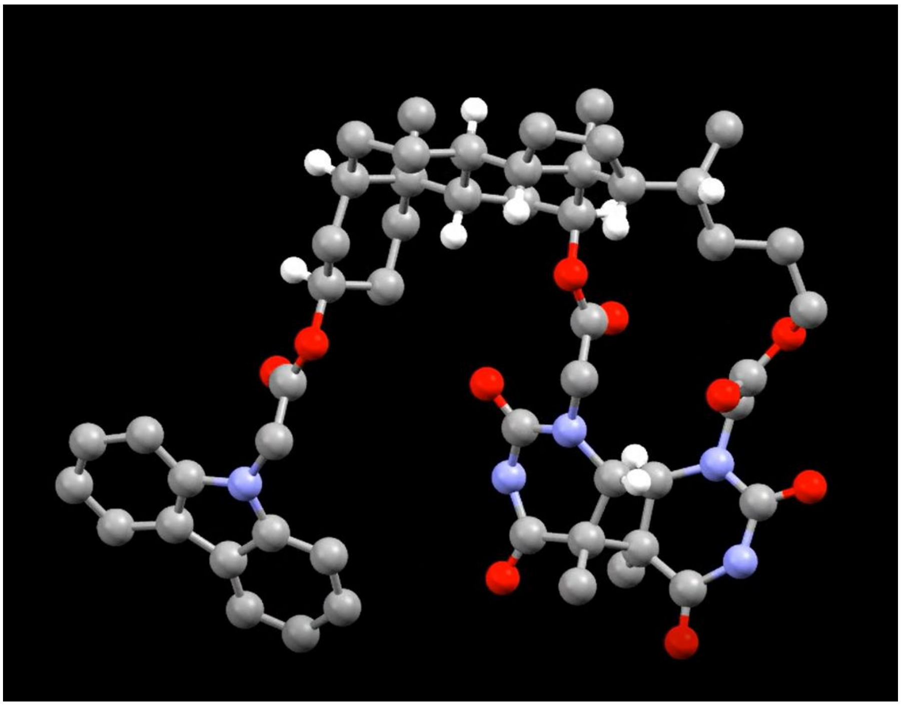

Figure S3.1. Molecular structure of **8**.

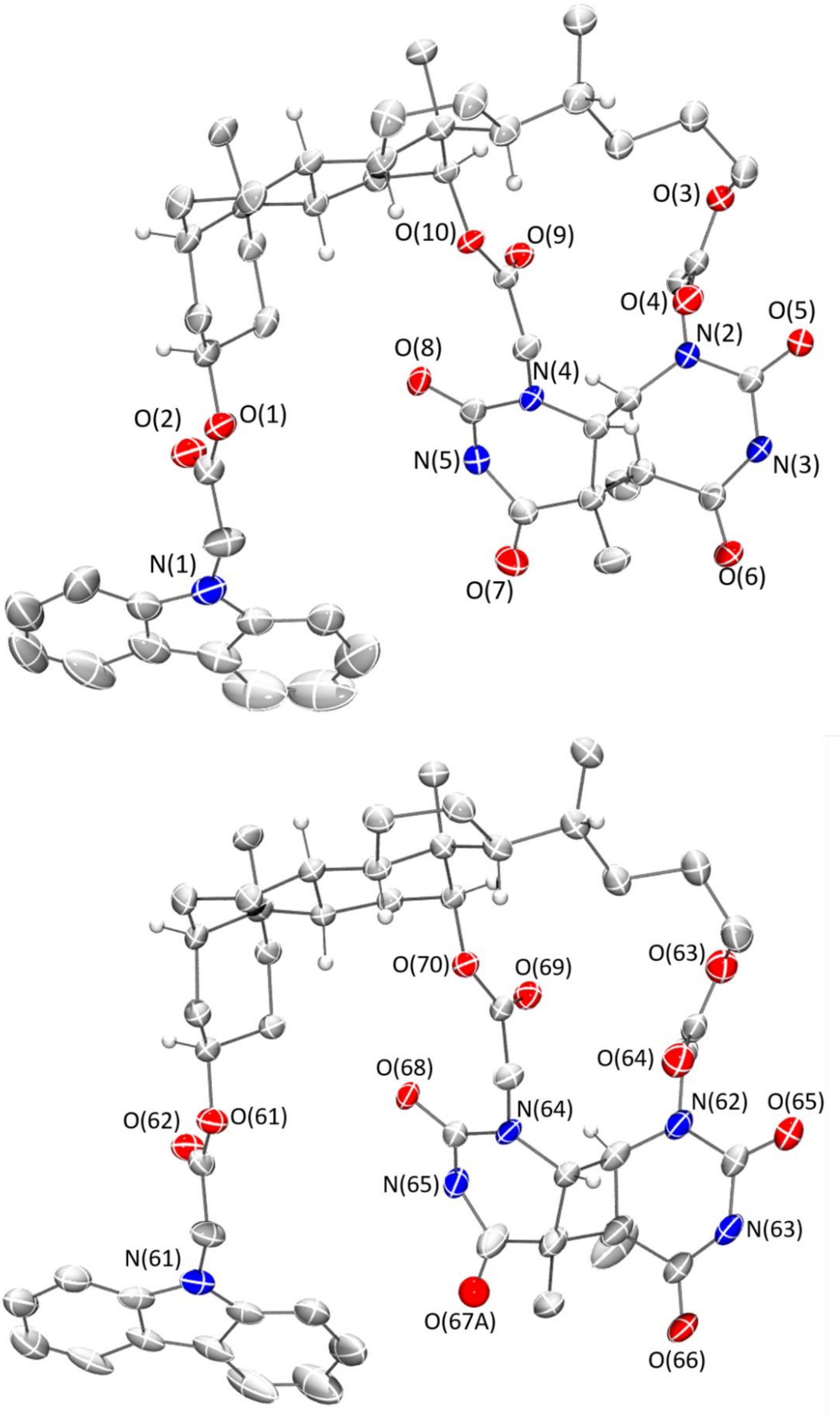

Figure S3.2. Molecular structure and heteroatoms labelling scheme of both independent molecules of compound **8**. Thermal ellipsoids drawn at the 50% probability level. For clarity, most of the hydrogen atoms have been supressed.

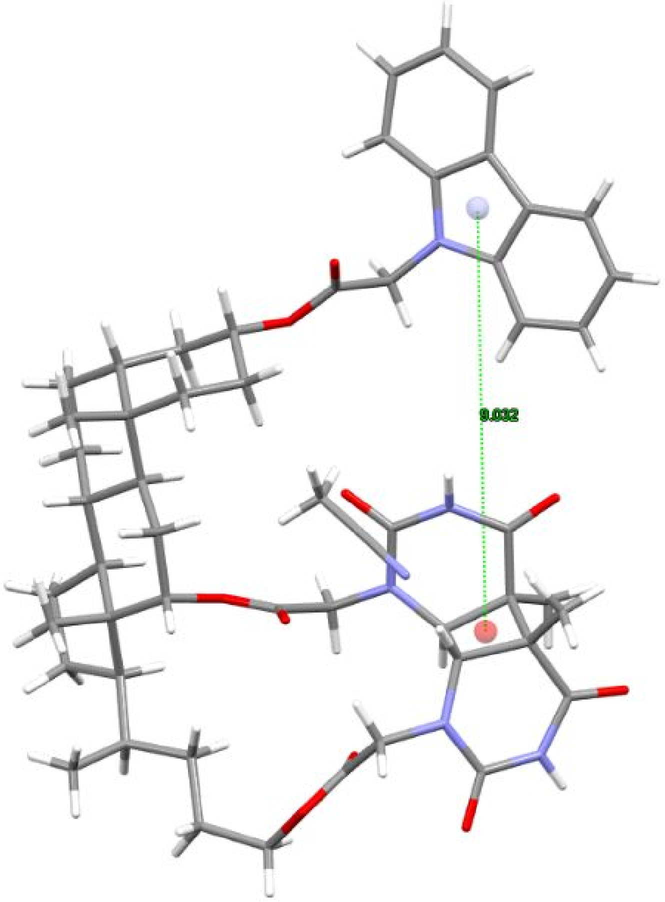

Figure S3.3. Molecular structure of **8** with detailed distance carbazol-Thy<>Thy.

S4. Synthesis

S4.1. Synthesis and characterization of 9

Benzyl alcohol (0.006 mL, 0.6 mmol) and titanium (IV) isopropoxide (0.017 mL, 0.057 mmol) were added to a solution of ester **7** (0.052 g, 0.057 mmol) in toluene (0.3 mL). The resulting mixture was heated at 120°C within a metallic block on the stirring plate until the starting material completely disappeared. After cooling to rt, the crude product was directly purified by flash chromatography (EtOAc:hexane, 3.5:1.5), affording ester **9** (0.029 g, 0.035 mmol, 62%, Scheme S4.1). ^{1}H NMR (400 MHz, $CDCl_3$) δ (ppm) 8.58 (s, 1H, Thy-NH), 8.40 (s, 1H, Thy-NH), 7.24-7.38 (m, 5H, arom), 5.12 (br s, 3H, CH_2+12β-H), 4.92 (m, 1H, 3β-H), 4.35-4.56 (m, 2H, Thy-CH_2), 4.16 (br s, 1H, Thy<>Thy-CH), 3.60 (br s, 1H, Thy<>Thy-CH), 3.40-3.56 (m, 2H, Thy-CH_2), 1.68 (s, 3H, Thy-CH_3), 1.59 (s, 3H, Thy-CH_3), 0.88 (s, 3H, CH_3), 0.83 (d, J = 4.5 Hz, 3H, 21-CH_3), 0.75-2.00 (complex signal, 26H), 0.70 (s, 3H, CH_3); ^{13}C{1H} NMR (100 MHz, $CDCl_3$) δ (ppm) 174.3 (C), 168.4 (C), 168.2 (C), 166.6 (2xC), 150.5 (C), 150.4 (C), 136.0 (C), 128.7 (2xCH), 128.5 (2xCH), 128.3 (CH), 79.0 (CH), 74.1 (CH), 66.5 (CH2), 63.6 (CH), 52.5 (CH), 51.3 (CH2), 49.4 (CH_2), 46.7 (CH), 45.3 (2xC), 45.0 (C), 39.9 (CH), 34.9 (CH), 34.5 (CH), 34.0 (CH_2), 33.2 (CH), 32.4 (C), 31.0 (CH), 30.6 (CH_2), 30.2 (CH_2), 29.6 (CH_2), 27.4 (CH_2), 25.7 (CH_2), 25.6 (CH_2), 25.1 (CH_2), 23.4 (CH_2), 23.1 (CH_2), 22.8 (CH_3), 22.4 (CH_3), 21.5 (CH_3), 17.1 (CH_3), 12.5 (CH_3); HRMS (ESI-TOF) m/z: [M + H]+ Calcd for $C_{45}H_{59}N_4O_{10}$ 815.4232; Found 815.4212.

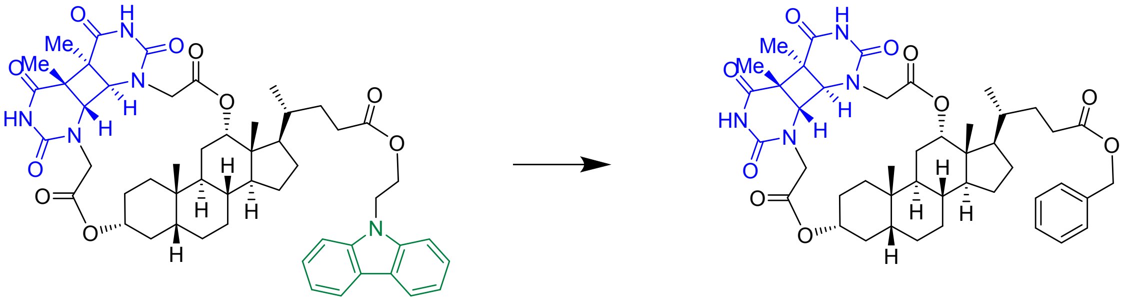

7 **9**

Scheme S4.1. Transesterification of **7** to yield **9**.

S4.2. ^{1}H, $^{13}C\{1H\}$, $^{13}C\{1H\}$ DEPT-135, ^{1}H-^{13}C HSQC, ^{1}H-^{1}H COSY and ^{1}H-^{1}H NOESY NMR of **9**

^{1}H NMR (400 MHz, $CDCl_3$)

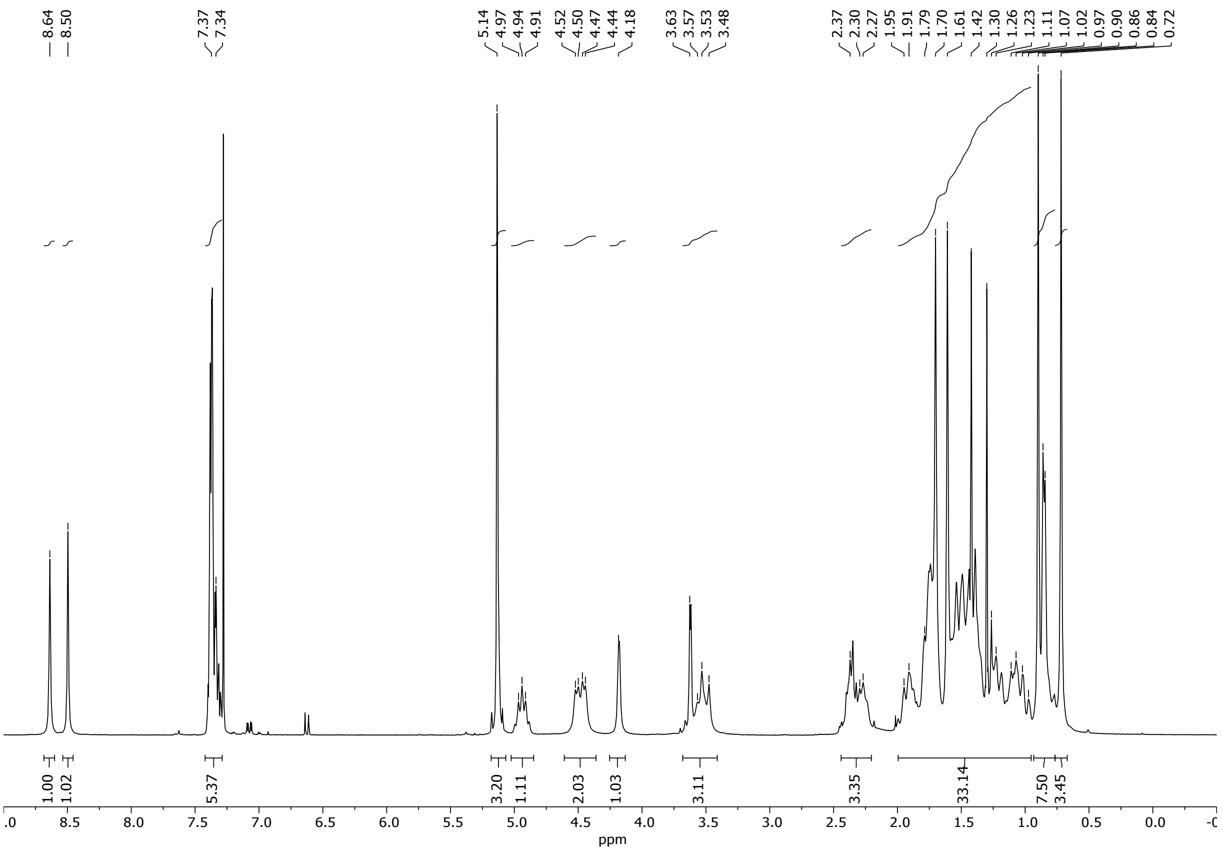

$^{13}C\{1H\}$ NMR (100 MHz, $CDCl_3$)

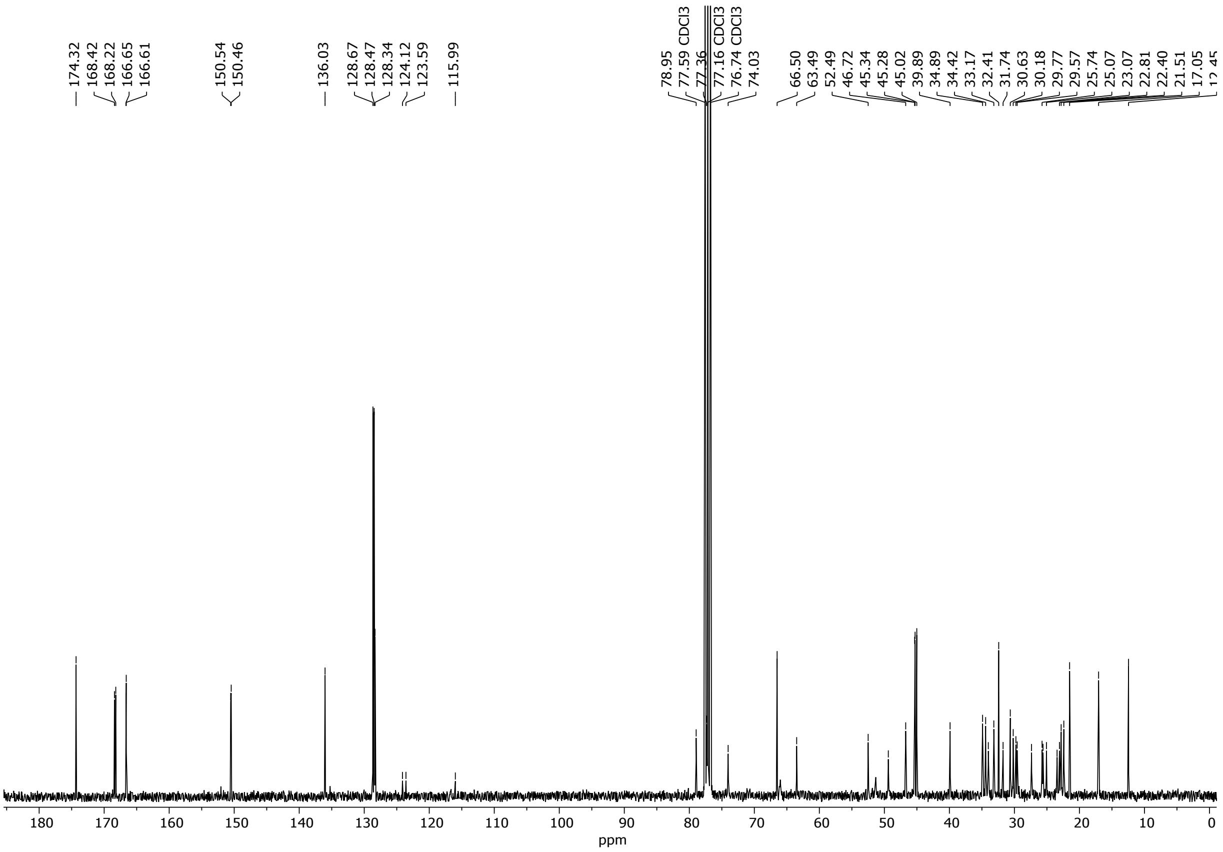

$^{13}C\{1H\}$ DEPT-135 (100 MHz, $CDCl_3$)

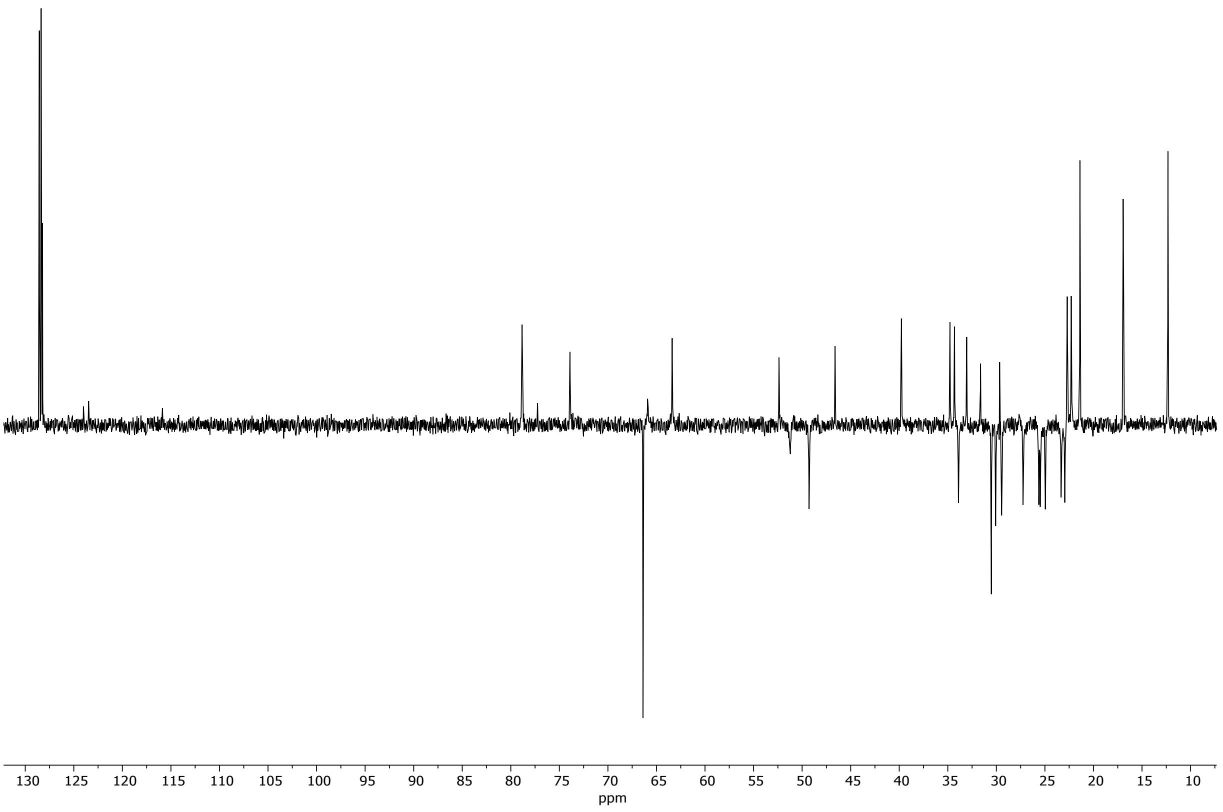

^{1}H-^{13}C HSQC ($CDCl_3$)

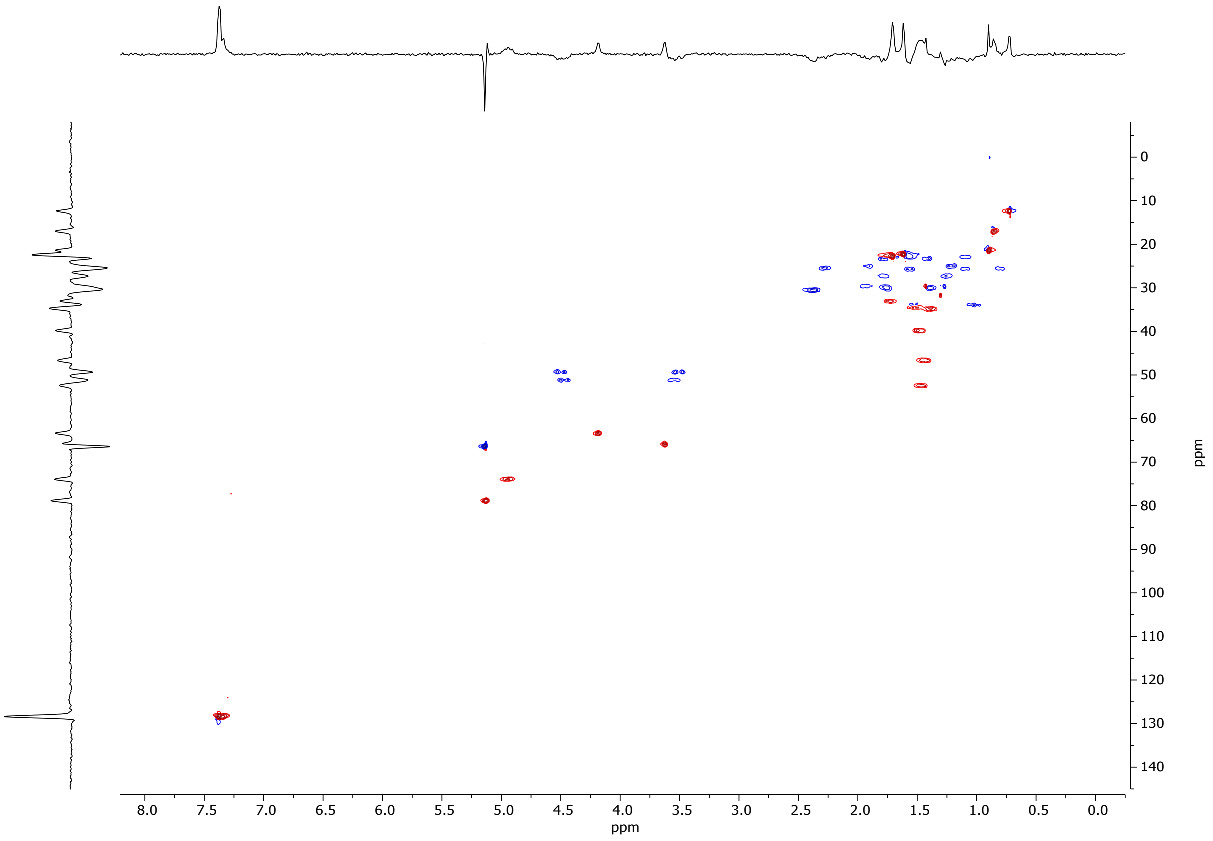

^{1}H-^{1}H COSY ($CDCl_3$)

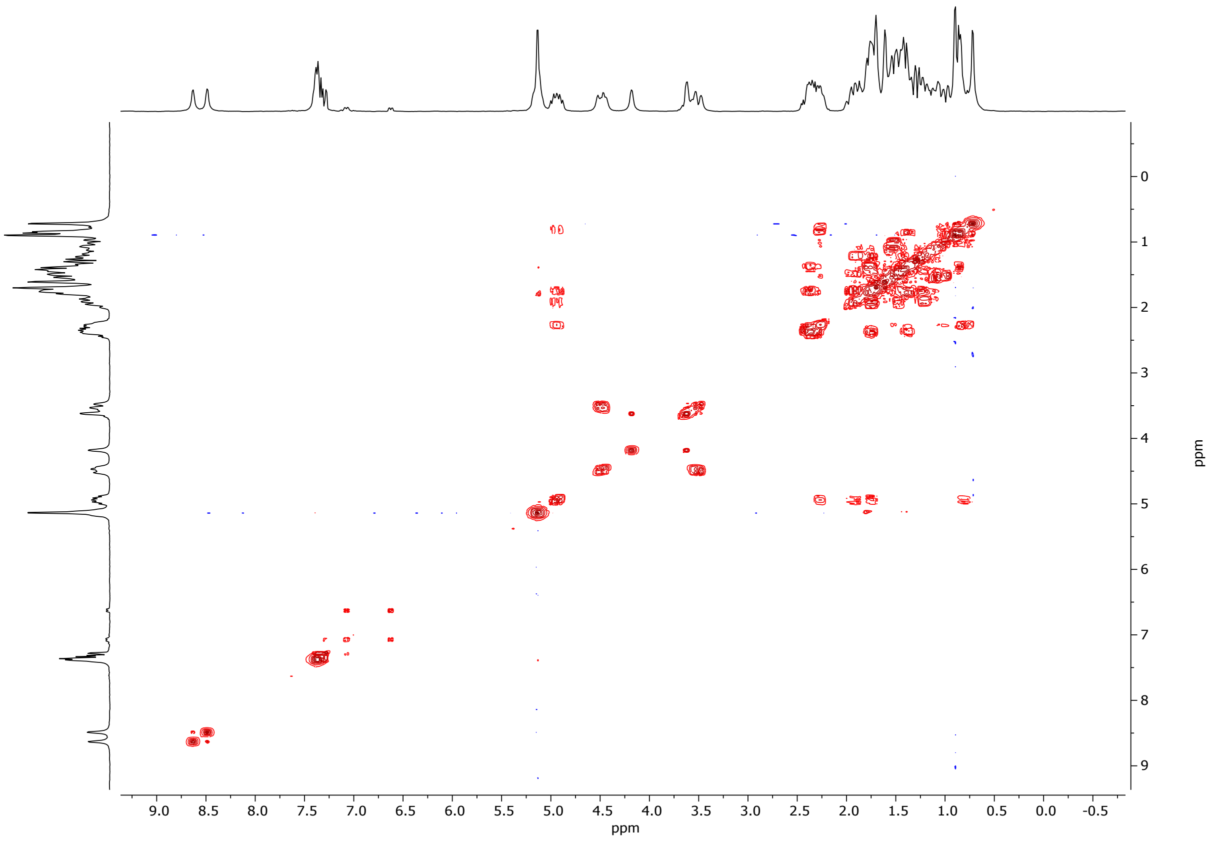

^{1}H-^{1}H NOESY ($CDCl_3$)

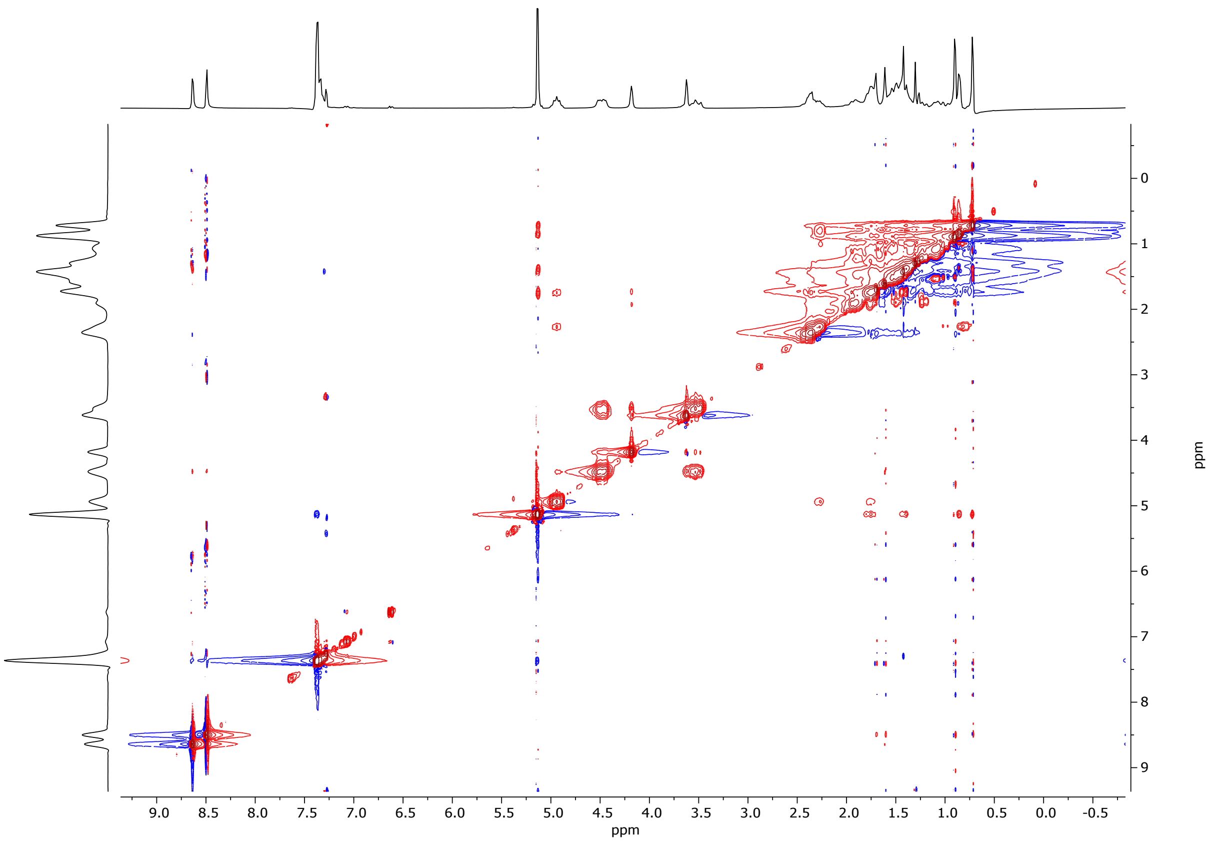

S4.3. Synthesis and characterization of **10**

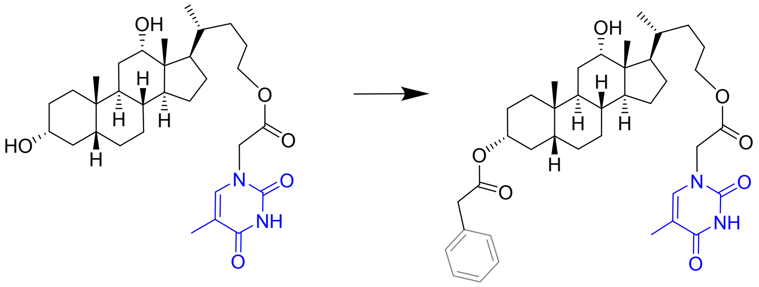

4 **10**

To a stirred solution of **4** (0.40 g, 0.73 mmol) and TBTU (0.35 g, 1.10 mmol) in anhydrous DMF (3 mL), phenylacetic acid (0.13 g, 0.96 mmol) in anhydrous DMF (2 mL) followed by DIEA (0.47 mL, 2.72 mmol) were added dropwise and then the reaction mixture was allowed to react at room temperature for 7 h. Afterwards, it was poured into brine and extracted with CH_2Cl_2; the combined organic layers were washed with brine, dried over $MgSO_4$ and concentrated under vacuum. Purification by column chromatography (SiO_2, EtOAc:Hexane, 50:50) gave **10** as a yellow solid (0.24 g, 49%); ^{1}H NMR (300 MHz, $CDCl_3$) δ (ppm) 8.51 (s, 1H, Thy-NH),7.19-7.33 (m, 5H, arom), 6.91 (br s, 1H, Thy-CH), 4.70 (m, 1H, 3β-H), 4.40 (s, 2H, Thy-CH_2), 4.13 (m, 2H, CH_2), 3.97 (br s, 1H, 12β-H), 3.55 (s, 2H, Ph-CH_2), 1.91 (d, J = 1.2 Hz, 3H, Thy-CH_3), 0.95 (d, J = 6.6 Hz, 3H, 21-CH_3), 0.88 (s, 3H, CH_3), 0.79-1.95 (complex signal, 26H), 0.65 (s, 3H, CH_3); ^{13}C{1H} NMR (75 MHz, $CDCl_3$): δ (ppm) 171.3 (C), 167.6 (C), 163.9 (C), 150.7 (C), 140.3 (CH), 134.5 (C), 129.3 (2xCH), 128.7 (2xCH), 127.1 (CH), 111.4 (C), 74.9 (CH), 73.3 (CH), 66.8 (CH_2), 48.8 (CH_2), 48.4 (CH), 47.6 (CH), 46.6 (C), 42.0 (CH), 41.9 (CH_2), 36.1 (CH), 35.2 (CH), 35.0 (CH_2), 34.3 (C), 33.8 (CH), 32.3 (CH_2), 31.9 (CH_2), 28.9 (CH_2), 27.7 (CH_2), 27.1 (CH_2), 26.7 (CH_2), 26.2 (CH_2), 25.2 (CH_2), 23.8 (CH_2), 23.3 (CH_3), 17.7 (CH_3), 12.9 (CH_3), 12.5 (CH_3); HRMS (ESI-TOF) m/z: [M + H]+ Calcd for $C_{39}H_{55}N_2O_7$ 663.4009; Found 663.4017.

S4.4. ^{1}H and $^{13}C\{1H\}$ NMR of **10**

^{1}H NMR (300 MHz, $CDCl_3$)

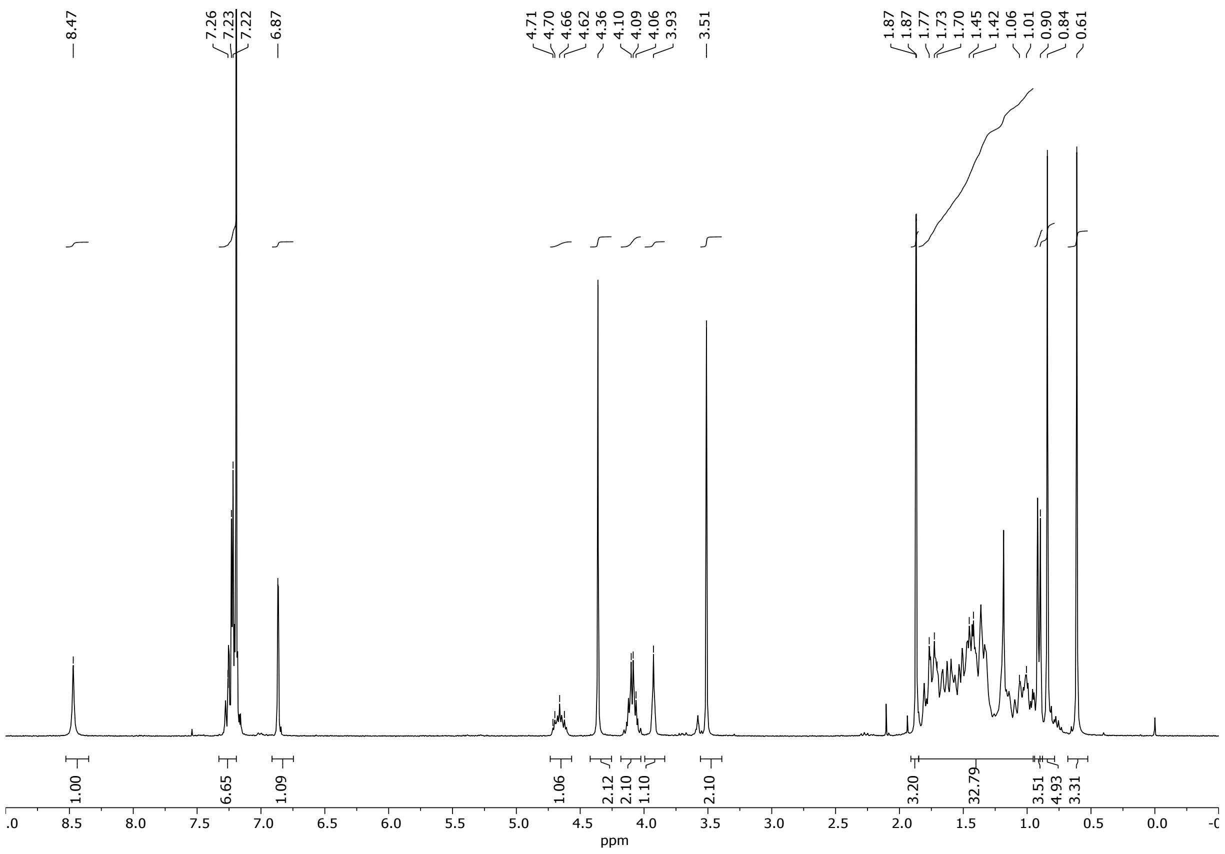

$^{13}C\{1H\}$ NMR (75 MHz, $CDCl_3$)

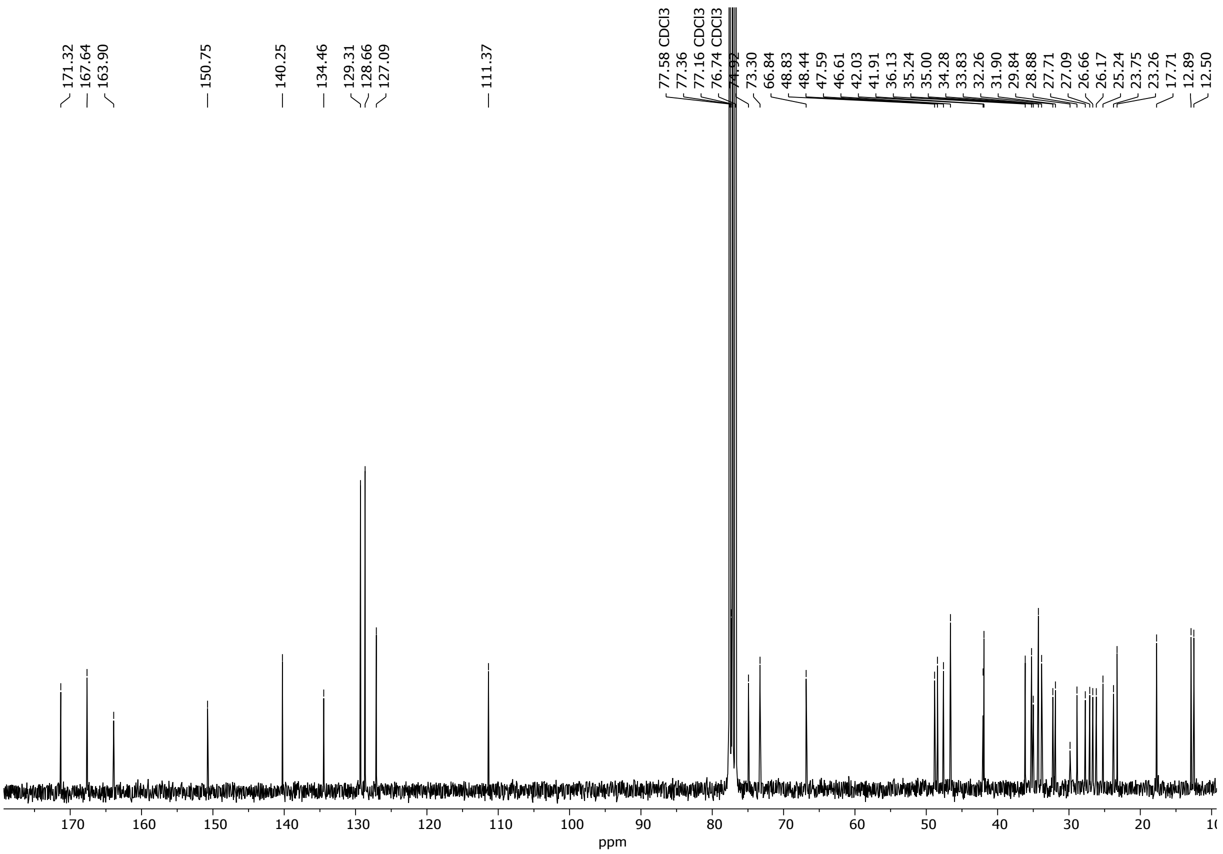

S4.5. Synthesis and characterization of **11**

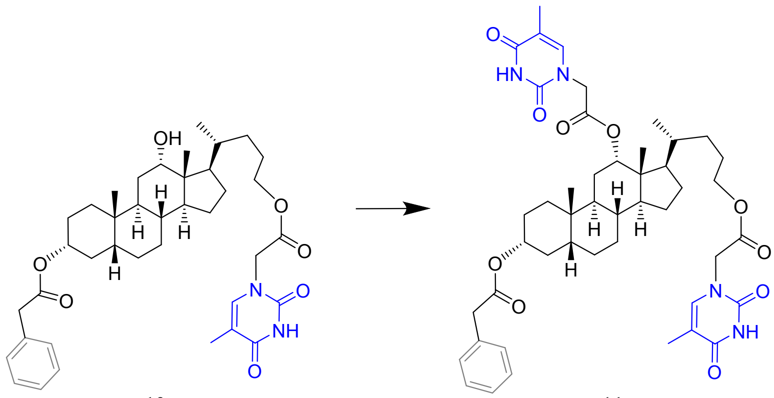

10 **11**

A stirred suspension of Thy-CH_2CO_2H (0.18 g, 0.95 mmol) and Et_3N (0.27 mL) in anhydrous THF (5 mL) was treated with 2,4,6-trichlorobenzoyl chloride (0.18 mL, 1.14 mmol). The resulting suspension was allowed to react for 1.5 h; after that, a solution of 4-DMAP (0.047 g, 0.39 mmol) and **10** (0.21 g, 0.32 mmol) in anhydrous THF (6.5 mL) was added and the reaction mixture was allowed to stir at room temperature overnight. Then, it was poured into brine and extracted with CH_2Cl_2. The combined organic extracts were washed with brine, dried over $MgSO_4$ and concentrated under vacuum. Purification by column chromatography (SiO_2, EtOAc:Hexane, 60:40) gave **11** as a pale yellow solid, 0.16 g, 61%); ^1H NMR (300 MHz, $CDCl_3$) δ (ppm) 11.05 (s, 1H, Thy-NH), 10.85 (s, 1H, Thy-NH), 7.17-7.34 (m, 5H, arom), 6.99 (br s, 1H, Thy-CH), 6.56 (br s, 1H, Thy-CH), 5.12 (br s, 1H, 12β-H), 4.78 (m, 1H, 3β-H), 4.69 (d, J = 17.4 Hz, 1H, Thy-CH_2), 4.55 (d, J = 17.1 Hz, 1H, Thy-CH_2), 4.23 (d, J = 17.1 Hz, 1H, Thy-CH_2), 4.22 (m, 2H, CH_2), 3.80 (d, J = 17.4 Hz, 1H, Thy-CH_2), 3.68 (d, J = 15.0 Hz, 1H, Ph-CH_2), 3.61 (d, J = 15.0 Hz, 1H, Ph-CH_2), 1.96 (d, J = 0.9 Hz, 3H, Thy-CH_3), 1.91 (d, J = 0.9 Hz, 3H, Thy-CH_3), 0.89 (s, 3H, CH_3), 0.78-2.05 (complex signal, 26H), 0.78 (d, J = 6.0 Hz, 3H, 21-CH_3), 0.67 (s, 3H, CH_3); ^{13}C {1H} NMR (75 MHz, $CDCl_3$) δ (ppm) 170.9 (C), 167.4 (C), 166.1 (C), 165.1 (C), 164.5 (C), 152.2 (C), 151.5 (C), 140.5 (CH), 140.2 (CH), 134.6 (C), 129.2 (2xCH), 128.8 (2xCH), 127.2 (CH), 111.7 (C), 111.0 (C), 78.2 (CH), 74.7 (CH), 66.7 (CH2), 50.4 (CH_2), 50.2 (CH_2), 50.1 (CH), 48.0 (CH), 45.1 (C), 42.2 (CH_2), 41.6 (CH), 35.8 (CH), 35.4 (CH), 34.7 (CH_2), 34.1 (CH), 33.8 (C), 32.1 (CH_2), 31.7 (CH_2), 27.4 (CH_2), 26.7 (CH_2), 26.5 (CH_2), 26.3 (CH_2), 26.2 (CH_2), 25.0 (CH_2), 23.3 (CH_2), 22.8 (CH_3), 17.6 (CH_3), 12.6 (CH_3), 12.5 (CH_3), 12.4 (CH_3); HRMS (ESI-TOF) m/z: [M + H]+ Calcd for $C_{46}H_{61}N_4O_{10}$ 829.4388; Found 829.4363.

S4.6. ^{1}H and ^{13}C{1H} NMR of **11**

^{1}H NMR (300 MHz, CDCl$_3$)

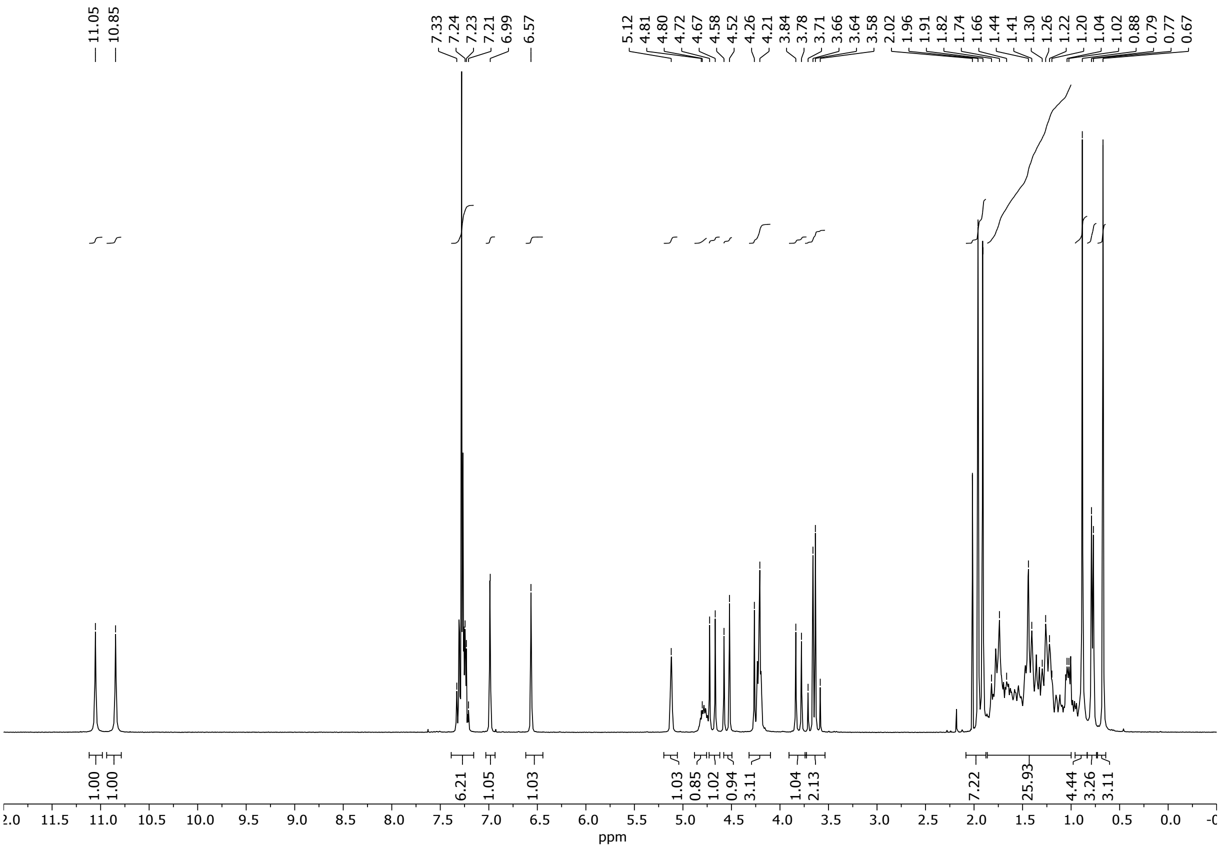

$^{13}C\{^1H\}$ NMR (75 MHz, $CDCl_3$)

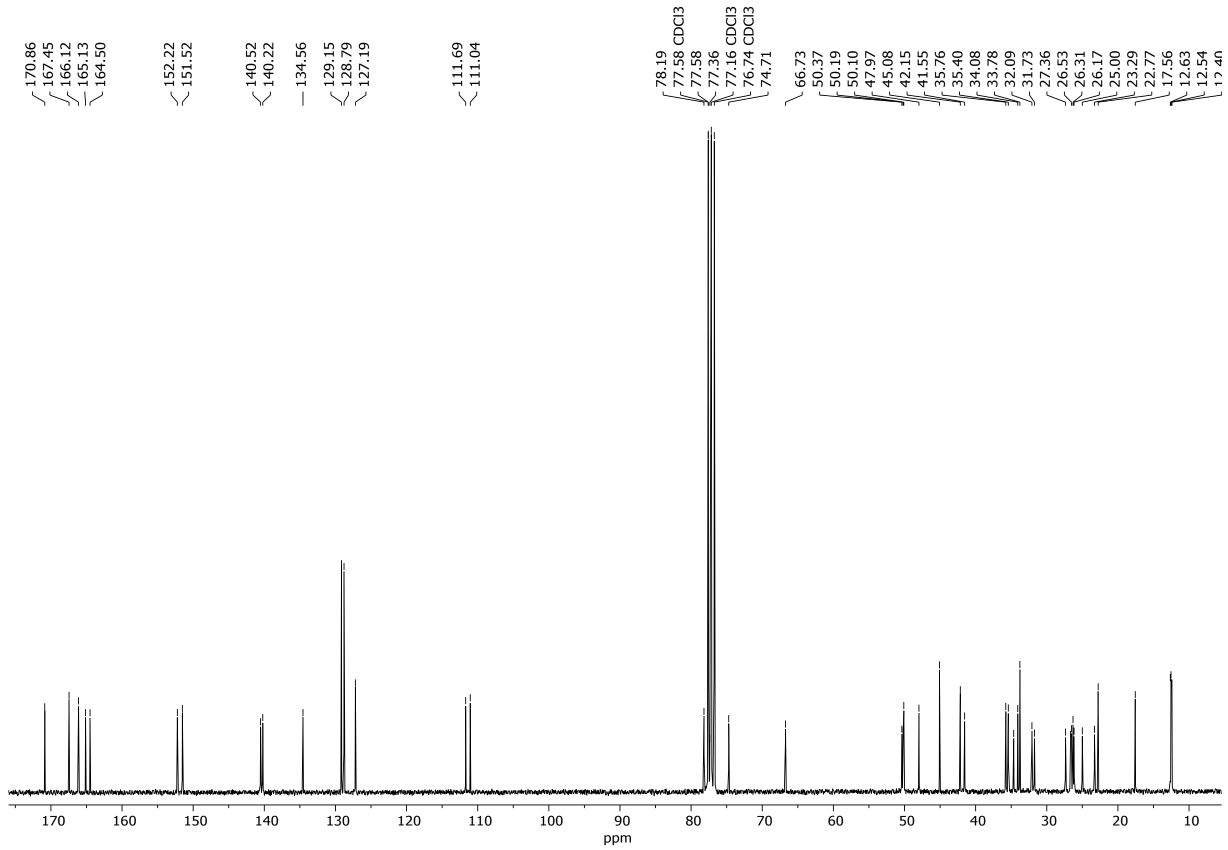

S5. Control experiments in the photosensitized Thy<>Thy dimers formation.

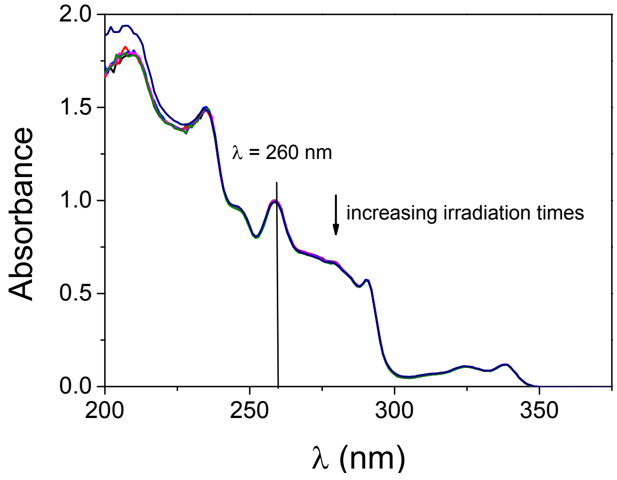

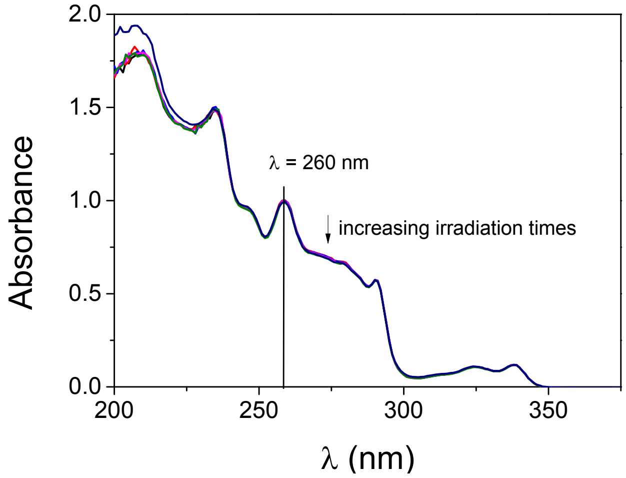

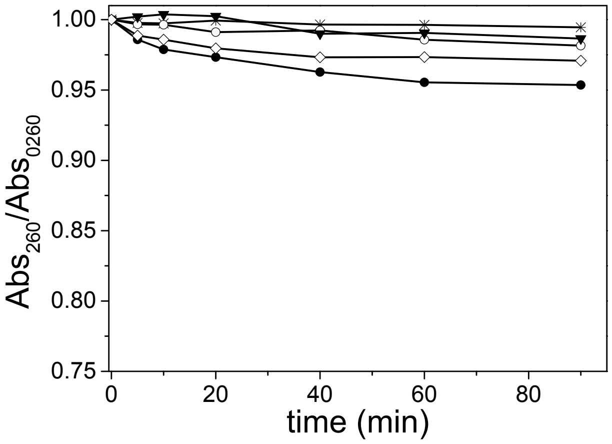

Figure S5.1. Top: UV-Vis spectra of **2** recorded at different irradiation times. **Middle:** UV-Vis spectra of **6** recorded at different irradiation times. **Bottom:** Photoreaction kinetics of Thy (as $ThyCH_2CO_2H$) (▼), Cbz (as $Cbz\text{-}CH_2CH_2OH$) (*), the intermolecular 2Thy:1Cbz mixture (○) and the intramolecular systems **2** (●) and **6** (◊). All reactions were performed upon irradiation at 350 nm, in areated $4CH_3CN{:}1H_2O$.

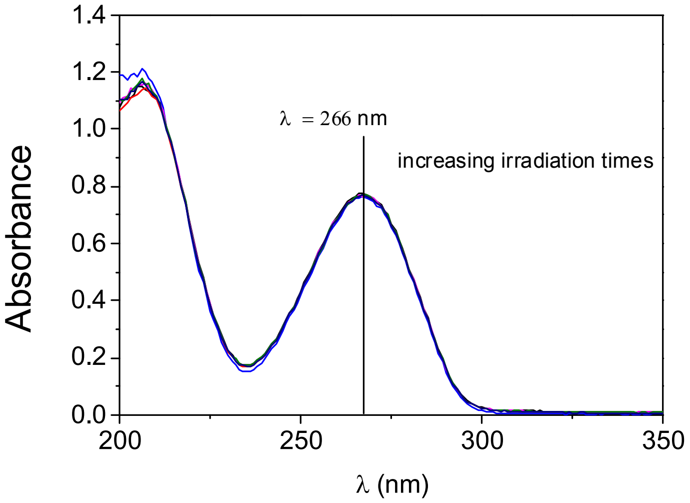

Figure S5.2. UV-Vis spectra of **11** recorded at prolonged irradiation times (λ_{max} = 350 nm), under anaerobic atmosphere.

S6. Control experiments in the photosensitized Thy<>Thy repair

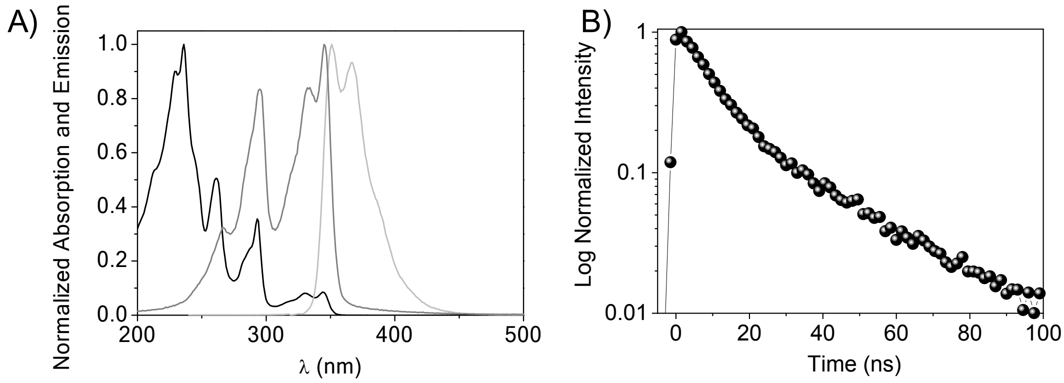

Figure S6.1. Normalized absorption (black), excitation (grey) and emission spectra (light gray) (A) and time-resolved fluorescence (B) of $CbzCH_2CH_2OH$ in CH_3CN: H_2O (4 : 1) mixture solution.

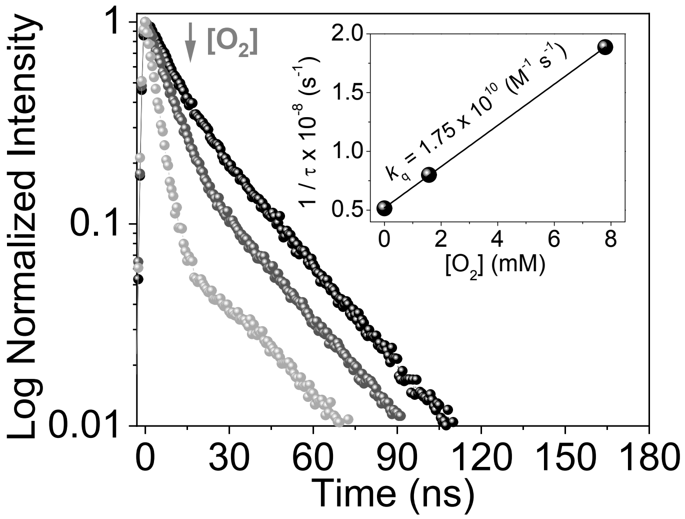

Figure S6.2. Time-resolved emission decay traces for $CbzCH_2CH_2OH$ upon increasing O_2 concentrations (λ_{exc} = 340 nm). Inset: corresponding Stern-Volmer plot.

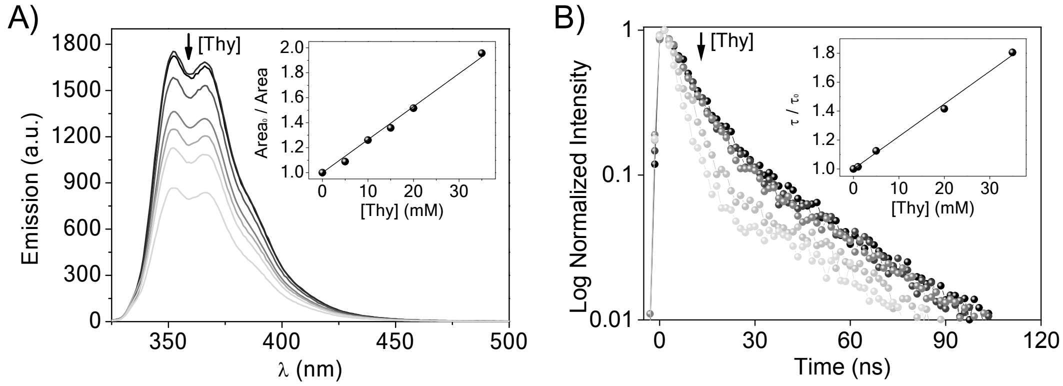

Figure S6.3. Changes in the emission spectra (left) and emission decay traces (right), λ_{exc} = 340 nm, of $CbzCH_2CH_2OH$ (50 μM) upon addition of increasing concentrations of $ThyCH_2CO_2H$ (up to 35 mM) recorded in aerated $4CH_3CN{:}1H_2O$. Insets: corresponding Stern−Volmer plots. A value of k_{qS} = 2.1 x 10^9 $M^{-1}s^{-1}$ was determined from the time-resolved experiments.

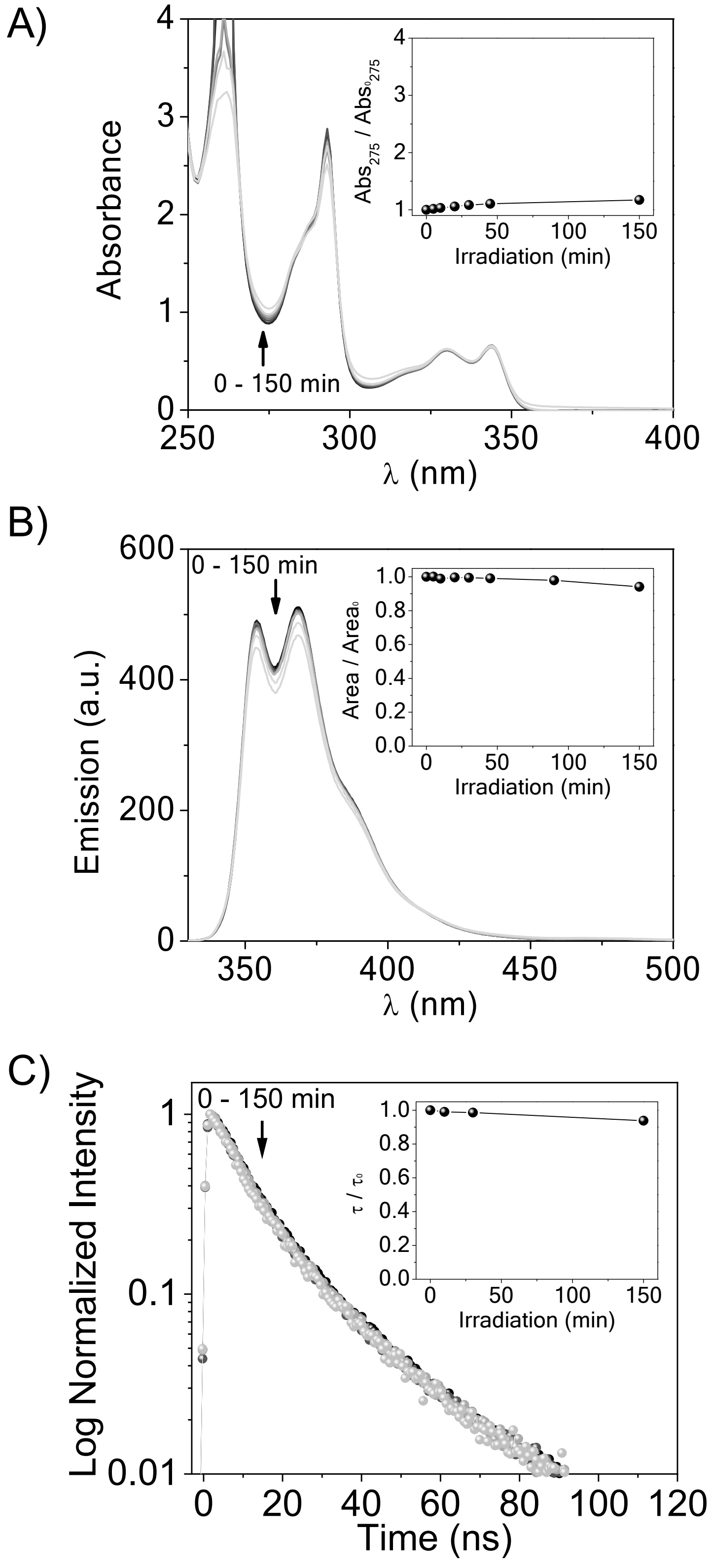

Figure S6.4. Kinetics of the evolution of commercial $CbzCH_2CH_2OH$ upon increasing irradiation times (λ_{exc} = 350 nm), at 0.2 mM in aerated $4CH_3CN{:}1H_2O$. A) Changes in the absorbance spectra (Inset: relative absorbance changes); B) Changes in the steady-state emission spectra (Inset: relative emission changes), λ_{exc} = 340 nm; C) Changes in the time-resolved emission (Inset: relative lifetime changes), λ_{exc} = 340 nm.

S7. Computational methodology

Molecular dynamics were performed using the force field by Oie et al[1] for the intramolecular bonds, angles and dihedrals. Van der Waals interactions between atom pairs in the system, excluding bonded atoms and 1-3 bonded, have been approximated by a Lennard-Jones 12-6 potential according to the UFF (Universal Force-field) parameterisation[2].

The DL_POLY[3] software (version 2.20) has been used, able to run in parallel for the evaluation of the energy and first derivatives using MPI, based on a replicated data algorithm. The molecular dynamic simulations were carried out including full flexibility for all the atoms of the system as well as periodic boundary conditions with a cubic box of 50 A containing one molecule of either **2**, **6**, **7** or **8**, plus the corresponding number of solvent molecules with the condition that the acetonitrile/water ratio is 4, with an average density of the solvent of 0.7975 g/cm^3 at 298 K[4]. The number of water molecules is 328, and the number of acetonitrile molecules is 1290, inside the cubix box of 50 Å. The temperature chosen is 298 K within the NVE ensemble. We employed the Verlet-leapfrog integration algorithm and the Evans thermostat, with a timestep of 1 fs. Each run comprised an equilibration stage of 5×10^4 steps followed by the necessary production stage so as to ensure sufficiently good statistics for the analysis of 2.95×10^6 steps (2.95 ns). The cutoff for the non-bonding forces was set to 9 Å, and the Ewald summation was employed for the Coulombic interactions. The configurations were saved every 10 time-steps (0.01 ps), giving a number of 3×10^5 configurations, which is a large number allowing to obtain good statistics and a smooth visualisation of the dynamics.

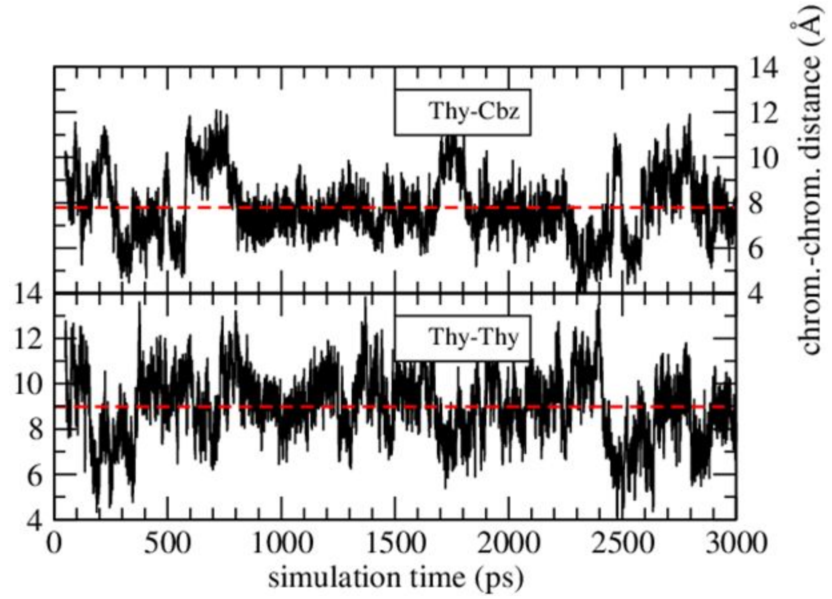

Figure S7.1. Plot of chromophore-chromophore distances of **2** along 3 ns obtained from molecular dynamics during 3 ns at 298 K in 4:1 acetonitrile:water solvent. The horizontal red dotted lines indicate the average distance.

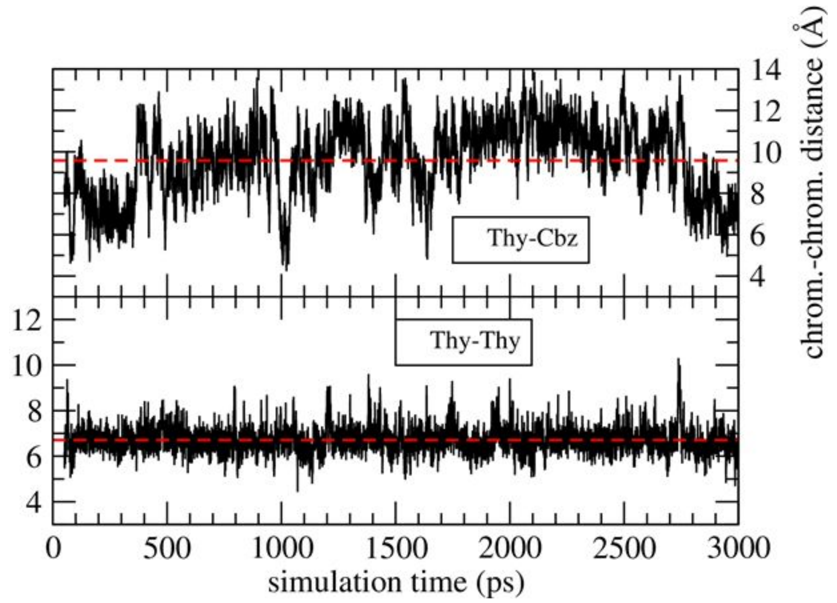

Figure S7.2. Plot of chromophore-chromophore distances of **6** along 3 ns obtained from molecular dynamics during 3 ns at 298 K in 4:1 acetonitrile:water solvent. The horizontal red dotted lines indicate the average distance.

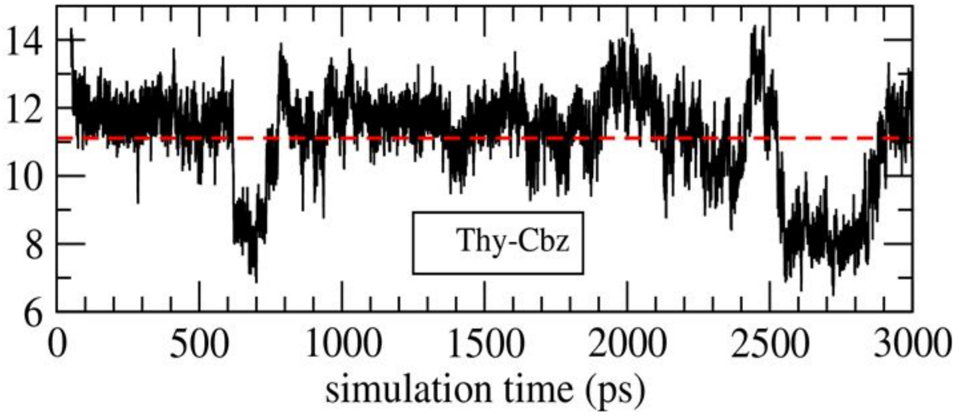

Figure S7.3. Plot of chromophore-chromophore distance of **7** along 3 ns obtained from molecular dynamics during 3 ns at 298 K in 4:1 acetonitrile:water solvent. The horizontal red dotted line indicates the average distance.

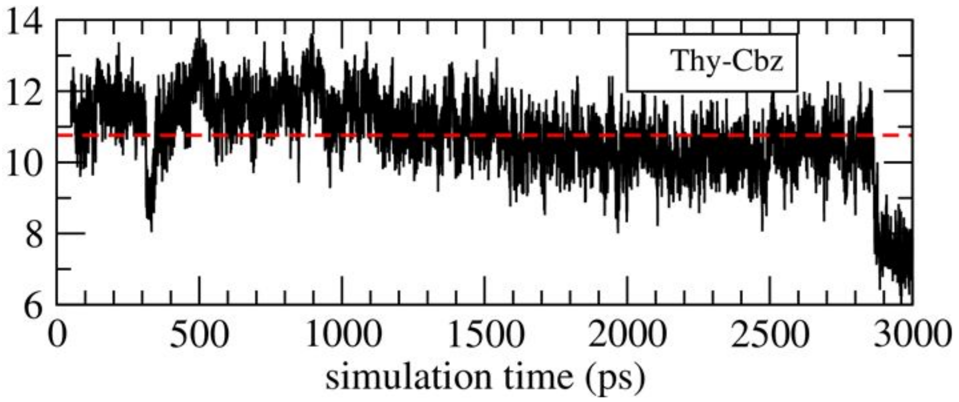

Figure S7.4. Plot of chromophore-chromophore distance of **8** along 3 ns obtained from molecular dynamics during 3 ns at 298 K in 4:1 acetonitrile:water solvent. The horizontal red dotted line indicates the average distance.

S7.1. References

1. T. Oie, T.M. Maggiora, R.E. Christoffersen and D.J. Duchamp, "Development of a Flexible Intra- and Intermolecular Empirical Potential Function for Large Molecular Systems", *Int. J. Quantum Chem. Quantum Biol. Symp.* **1981**, 8, 1-47.
2. A. K. Rappe, C. J. Casewit, K. S. Colwell, W. A. Goddard and W. M. Skiff, "UFF, a FullPeriodic Table Force Field for Molecular Mechanics and Molecular Dynamics Simulations", *J. Am. Chem. Soc.* **1992**, 114, 10024-10035.
3. W. Smith and T. R. Forester, "DL POLY 2.0: A general-purpose parallel molecular dynamics simulation package", *J. Mol. Graphics 1996*, 14, 136.
4. C. Moreau, G. Douheret, Thermodynamic Behaviour of Water-Acetonitrile mixtures. Excess Volumes and Viscosities, *Thermochim. Acta* **1975**, 13, 385-392.
